# Acetylides for the Preparation of Phosphorescent Iridium(III) Complexes: Iridaoxazoles and Their Transformation into Hydroxycarbenes and *N,C(sp^3^),C(sp^2^),O*-Tetradentate Ligands

**DOI:** 10.1021/acs.inorgchem.2c03522

**Published:** 2022-11-23

**Authors:** María Benítez, María L. Buil, Miguel A. Esteruelas, Susana Izquierdo, Enrique Oñate, Jui-Yi Tsai

**Affiliations:** † Departamento de Química Inorgánica, Instituto de Síntesis Química y Catálisis Homogénea (ISQCH), Centro de Innovación en Química Avanzada (ORFEO-CINQA), Universidad de Zaragoza−CSIC, 50009 Zaragoza, Spain; ‡ Universal Display Corporation, Ewing, New Jersey 08618, United States

## Abstract

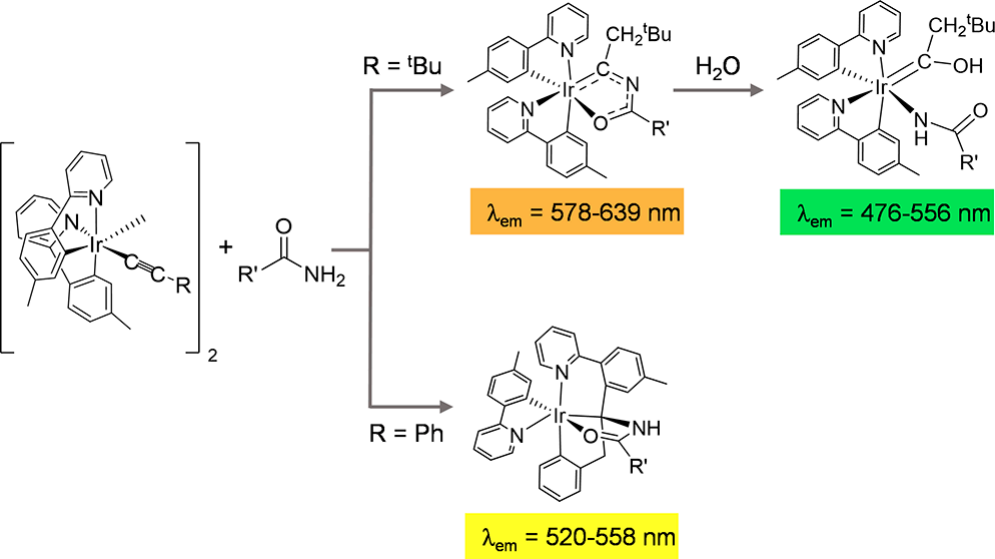

The preparation of three families of phosphorescent iridium(III) emitters, including iridaoxazole derivatives, hydroxycarbene compounds, and *N,C(sp^3^),C(sp^2^),O*-tetradentate containing complexes, has been performed starting from dimers *cis*-[Ir(μ^2^-η^2^-C≡CR){κ^2^-*C,N*-(MeC_6_H_3_-py)}_2_]_2_ (R = ^t^Bu (**1a**), Ph (**1b**)). Reactions of **1a** with benzamide, acetamide, phenylacetamide, and trifluoroacetamide lead to the iridaoxazole derivatives Ir{κ^2^-*C,O*-[C(CH_2_^t^Bu)NC(*R*)O]}{κ^2^-*C,N*-(MeC_6_H_3_-py)}_2_ (R = Ph (**2**), Me (**3**), CH_2_Ph (**4**), CF_3_ (**5**)) with a *fac* disposition of carbons and heteroatoms around the metal center. In 2-methyltetrahydrofuran and dichloromethane, water promotes the C–N rupture of the IrC–N bond of the iridaoxazole ring of **3–5** to form amidate–iridium(III)–hydroxycarbene derivatives Ir{κ^1^-*N-*[NHC(*R*)O]}{κ^2^-*C,N*-(MeC_6_H_3_-py)}_2_{=C(CH_2_^t^Bu)OH} (R = Me (**6**), CH_2_Ph (**7**), CF_3_ (**8**)). In contrast to **1a**, dimer **1b** reacts with benzamide and acetamide to give Ir{κ^4^-*N,C,C*′*,O*-[py-MeC_6_H_3_-C(CH_2_-C_6_H_4_)NHC(*R*)O]}{κ^2^-*C,N*-(MeC_6_H_3_-py)}(R = Ph (**9**), Me (**10**)), which bear a *N,C(sp^3^),C(sp^2^),O*-tetradentate ligand resulting from a triple coupling (an alkynyl ligand, an amide, and a coordinated aryl group) and a C–H bond activation at the metal coordination sphere. Complexes **2–4** and **6–10** are emissive upon photoexcitation, in orange (**2–4**), green (**6–8**), and yellow (**9** and **10**) regions, with quantum yields between low and moderate (0.01–0.50) and short lifetimes (0.2–9.0 μs).

## Abstract 2

Dimers *cis*-[Ir(μ^2^-η^2^-C≡CR){κ^2^-*C,N*-(MeC_6_H_3_-py)}_2_]_2_ allow to develop original synthetic procedures, which leads to iridium(III) phosphorescent emitters different to those previously known, including iridaoxazole compounds, hydroxycarbene derivatives, and complexes containing 6e-donor *N,C(sp^3^),C(sp^2^),O*-tetradentate ligands.

## 1 Introduction

The development of phosphorescent emitters, including those of iridium(III), mainly focuses on organic synthesis. Interesting chromophores and ancillary ligands are prepared by purely organic methods. Subsequently, they are coordinated to an appropriate 5d metal center by conventional procedures involving the activation of some of their σ bonds or directly.^1^ Once located in the coordination sphere of the metal, they can be modified by subsequent selective functionalization; a noticeable employed procedure involves C–H bromination of one or more ligands followed by a palladium-promoted Suzuki–Miyaura cross-coupling.^2^ A common feature of such emitters is their structural monotony. In the most classical complexes of this class, such as the iridium(III) species having two orthometalated arylpyridine groups, such monotony is clearly evident in the systematic mutually *trans* arrangement of the heterocycles,^3^ with a few rare exceptions.^4^

**Scheme 1:**
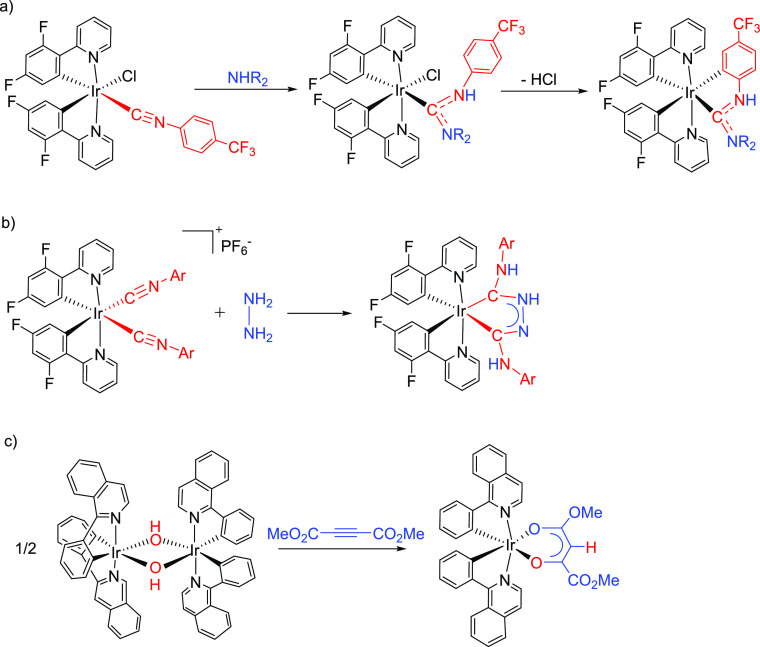
Previous Procedures to Build Ancillary Ligands of Iridium(III) Emitters at the Metal Coordination Sphere

Alternative procedures have been scarcely investigated (Scheme 1). Teets’ research group^5^ and in less extension Kinzhalov, Luzyanin, and coworkers^6^ have built ancillary ligands at the metal coordination sphere of bis(cyclometalated arylpyridine)iridium(III) complexes, using coordinated arylisocyanides as building blocks. Their reactions with amines have generated a variety of acyclic aryldiaminocarbene auxiliary ligands by the nucleophilic addition of the amine to the C(sp) carbon atom.^5,6^ In some cases, the aryl substituent of the resulting monodentate diaminecarbene was subsequently cyclometalated (a in Scheme 1) to afford interesting heterolytic tris-cyclometalated iridium(III) blue-green emitters of class [3b + 3b + 3b′] (3b = 3e donor bidentate ligand).^5^ Reactions of bis(arylisocyanide) precursors with hydrazine directly afforded related emitters where the 3b′ ligand is a *Chugaev-type* carbene (b in Scheme 1).^7^ Inspired by previous work about osmium chemistry,^8^ we have recently described a methodology of synthesis that, applied to the preparation of phosphorescent complexes, allows to generate emitters of the class [3b + 3b + 3b′] with an asymmetrical β-diketonate ligand. The procedure involves the *anti*-addition of the O–H bond of a dihydroxo-bridged dimer [Ir(μ-OH)(3b)_2_]_2_ to the C–C triple bond of activated alkynes and the C–C double bond of α,β-unsaturated ketones (c in Scheme 1).^9^

**Scheme 2:**
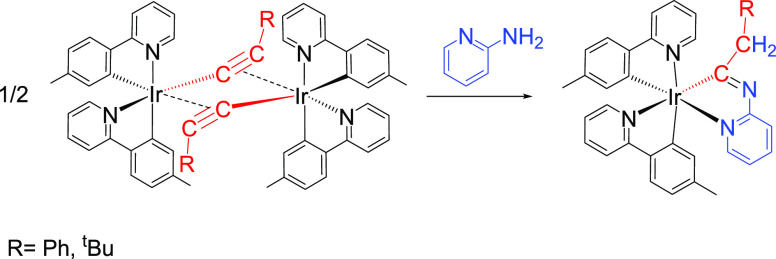
Synthesis of Iridaimidazo[1,2-*a*]pyridine Emitters

Synthetic procedures summarized in Scheme 1 have certainly allowed to generate novel ancillary ligands. However, the emitters maintain the mutually *trans* arrangement of the heterocycles of the chromophores. A recent review about some advances in synthesis of molecular heteroleptic osmium and iridium phosphorescent emitters has pointed out that improvements in the field would come over as a consequence of the development of new procedures of organometallic synthesis,^10^ for which the handling of alternative starting complexes is crucial. Dimers *trans*-[Ir(μ-Cl)(3b)_2_]_2_ have been traditionally the usual starting point for the preparation of heteroleptic emitters of classes [3b + 3b + 3b′]^11^ and [3b + 3b + 2m + 1m′]^12^ (2m = 2e donor monodentate, 1m = 1e donor monodentate). A handicap of these dimers, which appears to be responsible for the lack of structural diversity between the resulting emitters, is the retention of the stereochemistry of their mononuclear half during the emitter preparation process. In the search for starting materials to the synthesis of [3b + 3b + 3b′] emitters with a *cis* arrangement of the heterocycles of the 3b ligands, we recently replaced the chloride bridges of dimers *trans*-[Ir(μ-Cl)(3b)_2_]_2_ by acetylides. The action provided us two synthetic improvements: the mononuclear fragments of the new dimers *trans*-[Ir(μ^2^-η^2^-C≡CR)(3b)_2_]_2_ change the relative positions of the donor atoms of one of the 3b chelates to afford counterparts *cis*-[Ir(μ^2^-η^2^-C≡CR)(3b)_2_]_2_ with *cis*-heterocycles, in contrast to the chloride dimers as was desired, whereas the acetylide modifies and enhances the reactivity of the carbon atoms of the triple bond to be converted into an interesting building block, which generates new types of ligands. We thus prepared iridaimidazo[1,2-*a*]pyridine emitters of an octahedral structure with a *fac* disposition of carbon and nitrogen atoms (Scheme 2).^13^

**Scheme 3:**
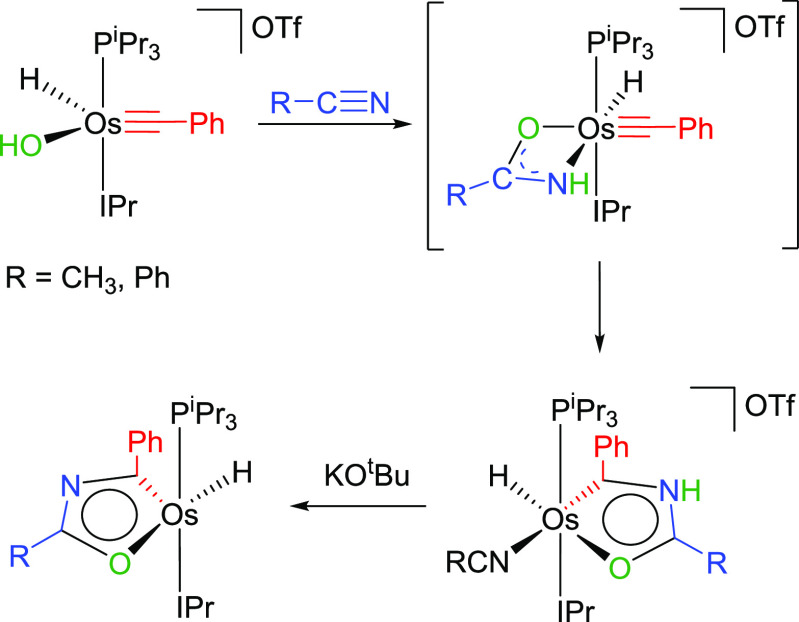
Preparation of Osmaoxazole Derivatives

The number of heteroaromatic organic molecules that might be in principle employed as a part of the chromophores or ancillary ligands of the emitters is extremely large.^14^ The formal replacement of a CH unit at a molecule of this type by an isolobal metal fragment, formed by a transition metal and its associated ligands, generates metalaheteroaromatic derivatives. Such compounds have a tremendous conceptual interest, since the metal fragment adds metal properties and organometallic reactivity to the aromatic organic heterocycle.^15^ Although the iridium–pyridine bond prevents the full aromaticity of the bicycle, the iridaimidazo[1,2-*a*]pyridine emitters shown in Scheme 2 are examples of this class of organometallic molecules. Previously, a few phosphorescent aromatic iridacarbocyclic derivatives had been reported.^16^ Metalaheteroaromatic compounds are mono- and polycyclic species bearing a main-group heteroatom. The first monocycles containing two main-group heteroatoms, osmaoxazole derivatives, were reported a few months ago. They were prepared by deprotonation of hydrideosmaoxazolium salts, which were generated via amidate intermediates. Such transient species resulted from the addition of the hydroxide group of the cation [OsH(OH)(≡CPh)(IPr)(P^i^Pr_3_)]^+^ (IPr = 1,3-bis(2,6-diisopropylphenyl)imidazolylidene) to an external nitrile or directly through displacement of the hydroxide group by an amidate anion. Once the amidate is generated, it cyclizes with the alkylidyne ligand to form the five-membered ring (Scheme 3).^17^

The formation of the osmaoxazoles exhibited in Scheme 3 resembles the cyclization shown in Scheme 2. In both cases, a nucleophilic nitrogen atom of a doubly deprotonated NH_2_ molecule adds to the α-atom of a C-donor ligand. Such similarity prompted us to investigate the addition of amides to the dimers *cis*-[Ir(μ^2^-η^2^-C≡CR){κ^2^-C*,N*-(MeC_6_H_3_-py)}_2_]_2_ (R = ^t^Bu, Ph), in the search for novel families of iridium(III) emitters. This paper reports about the synthesis and photophysical properties of complexes of three different unprecedented families of phosphorescent iridium(III) emitters of classes [3b + 3b + 3b′], [3b + 3b + 2 m + 1 m′], and [6tt + 3b] (6tt = 6e donor tetradentate), including the first iridaoxazole derivatives, hydroxycarbene compounds, and *N,C,C*′*,O*-tetradentate containing complexes. The described preparations illustrate alternative synthetic procedures in the generation of phosphorescent emitters and highlight again the utility of alkynyl ligands as building blocks in organometallic synthesis.

## 2 Results and Discussion

### 2.1 Iridaoxazole Derivatives

**Scheme 4:**
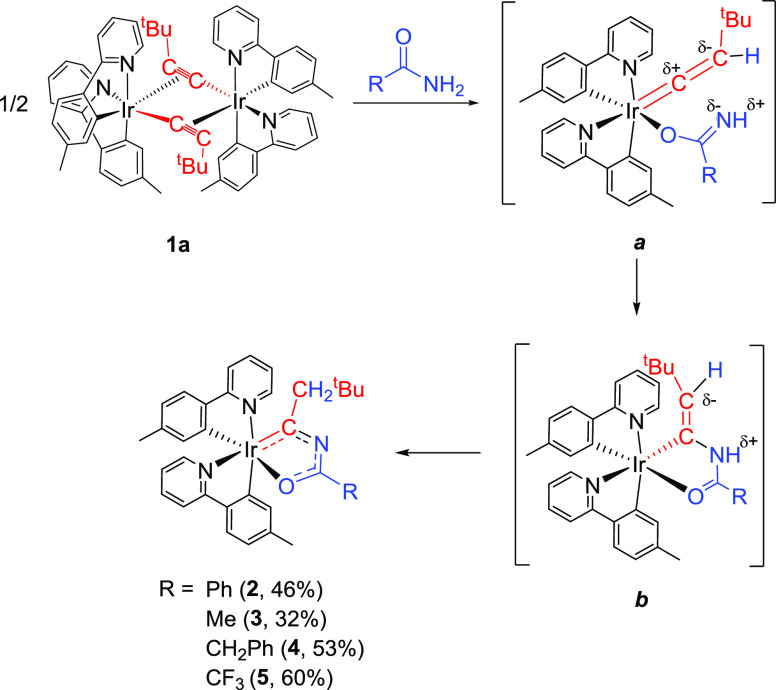
Formation of Iridaoxazole Derivatives

Treatment of suspensions of the *tert*-butylacetylide dimer *cis*-[Ir(μ^2^-η^2^-C≡C^t^Bu){κ^2^-*C,N*-(MeC_6_H_3_-py)}_2_]_2_ (**1a**), in toluene, with 2–3 equiv of benzamide, acetamide, phenylacetamide, and trifluoroacetamide, at 120 °C leads to the respective iridaoxazole derivatives Ir{κ^2^-*C,O*-[C(CH_2_^t^Bu)NC(*R*)O]}{κ^2^-*C,N*-(MeC_6_H_3_-py)}_2_ (R = Ph (**2**), Me (**3**), CH_2_Ph (**4**), CF_3_ (**5**)). Their formation is the result of the cleavage of the acetylide bridges of **1a** and the addition of the NH_2_ group of the amide to the C–C triple bond of the alkynyl ligand. The addition most probably occurs by stages (Scheme 4). As is well known, the coordination of an alkynyl group to a late transition metal produces the nucleophilicity transfer from C_α_ to C_β_ atoms of the triple bond, in such a way that the NH_2_ group of the amide could be deprotonated by the nucleophilic C_β_ atom of the alkyne to initially afford (κ^1^-*O*-amidate)-iridium-vinylidene intermediates ***a***.^18^ Thus, the subsequent attack of the nucleophilic NH group of the amidate to the electrophilic C_α_ atom of the vinylidene would lead to ***b***. The latter should experience a 1,3-hydrogen shift from the nitrogen atom of the five-membered ring to the exocyclic C–C double bond to finally form the iridaoxazole derivatives.

**Figure 1:**
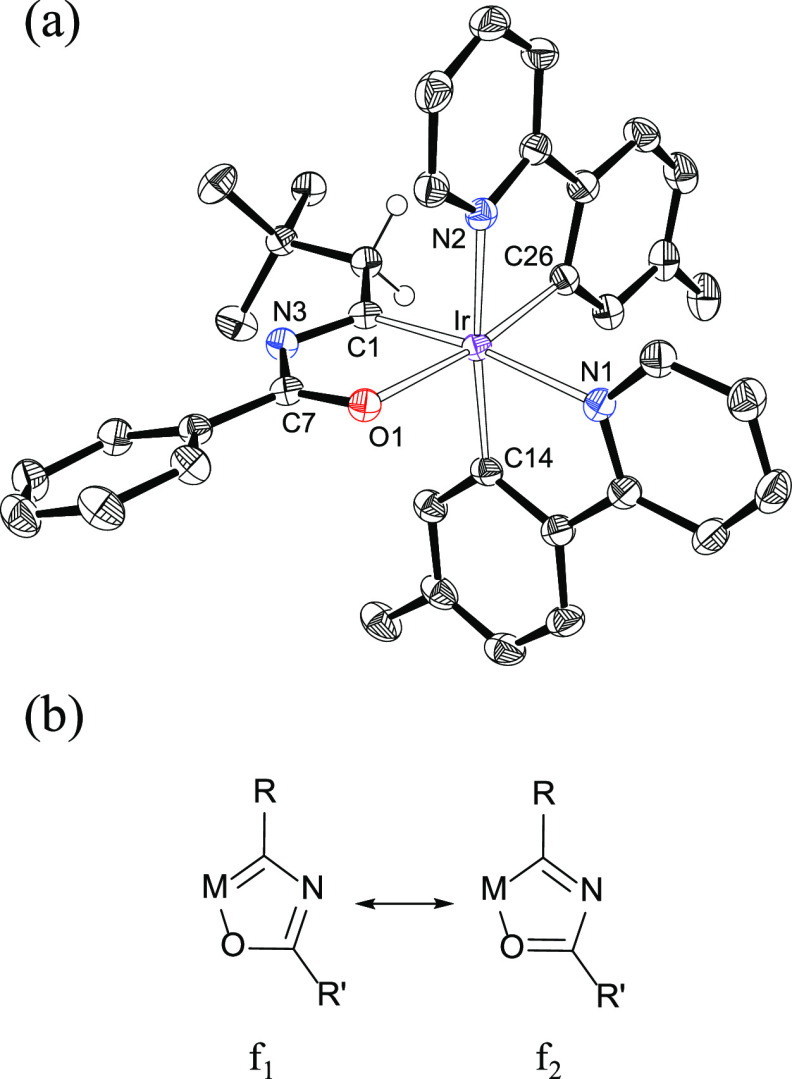
(a) ORTEP diagram of complex **2**. Only significant hydrogen atoms are shown for clarity. Selected bond lengths (Å) and angles (deg): Ir–N(1) = 2.1466(18), Ir–N(2) = 2.1131(18), Ir–O(1) = 2.1616(15), Ir–C(1) = 1.942(2), Ir–C(14) = 2.017(2), Ir–C(26) = 2.002(2), N(3)–C(1) = 1.343(3), N(3)–C(7) = 1.360(3), O(1)–C(7) = 1.263(3), C(1)–Ir–N(1) = 171.86(8), C(26)–Ir–O(1) = 171.80(7), C(14)–Ir–N(2) = 173.45(8). (b) Canonical form that describe the metalacycle bonding situation.

Complexes **2**–**5** were isolated as orange-red solids in 30–60% yield after 24 h and the corresponding purification of the reaction crude by column chromatography. The formation of the metaladiheterocycle was confirmed through the X-ray diffraction analysis structure of **2** (Figure 1a). The addition of the amide to the metal-acetylide moiety occurs with retention of the stereochemistry around each iridium center. Thus, its coordination polyhedron can be described as an octahedron formed by three (carbon, heteroatom)-chelating groups with a *fac* dispositions of carbons and heteroatoms. The iridaoxazole ring is planar. The maximum deviation from the best plane through the atoms Ir, C(1), N(3), C(7), and O(1) is 0.0275(11) Å and involves C(1). The bond lengths in the sequence C(1)–N(3)–C(7)–O(1) of 1.343(3), 1.360(3), and 1.263(3) Å are intermediate between those expected for single and double bonds, as expected for the contribution of both resonance forms **f_1_** and **f_2_** to the structure (Figure 1b) and compare well with the analogous ones in the osmaoxazole derivatives OsX{κ^2^-*C,O*-[C(Ph)NC(R)O]}(IPr)(P^i^Pr_3_) (X = H, R = CH_2_Ph; X = C≡CPh, R = Me).^17^ In spite of its planarity and the bond length values, the iridaoxazole ring is scarcely aromatic, as revealed by the poorly negative value of the nuclear independent chemical shifts (NICS_zz_) computed at the center of the ring and out of plane at 1 Å above and below of +17.8, −3.0, and – 3.2 ppm. The low aromaticity of these iridaoxazoles was furthermore confirmed by a NICS scan (Figure S1) and the anisotropy of the induced current density (ACID) method, which clearly shows the lack of a diatropic current within the ring (Figure S2). The main difference between the iridaoxazoles here reported and the osmaoxazoles previously described appears to be in the M–C bond of the metaladiheteroring. This bond appears to have an M-to-C back bonding component weaker in the formers than in the second ones. Consistent with this, comparative NBO7 analysis of the osmium derivative OsH{κ^2^-*C,O-*[C(Ph)NC(CH_3_)O]}(IPr)(P^i^Pr_3_) and the iridium counterpart **3** (Figure S3a**)** revealed that the Wiberg bond index of the M–C bond of the five-membered ring is 1.28 for osmaoxazole, while it has a value of only 0.84 for iridaoxazole. In agreement with this, π NBO orbitals of the five-membered metalaoxazole rings (Figure S3b) indicate that the resonance form **f_1_** is the major contribution to the osmaoxazole structure, while **f_2_** is the most relevant for the iridaoxazole structure. As a consequence of the weak back bonding, the metalated carbon atom of the iridaoxazole ring of **2–5** seems to undergo a noticeable electron deficiency in comparison with the analogous atom of the osmaoxazoles. In this context, it should be pointed out that the resonance corresponding to the metalated carbon atom of the metaladiheterocycle in the ^13^C{^1^H} NMR spectra of **2–5** appears in the 267–280 ppm range, shifted about 40 ppm to lower field with regard to the osmaoxazole derivatives.

### 2.2 Hydroxycarbene Compounds

**Scheme 5:**
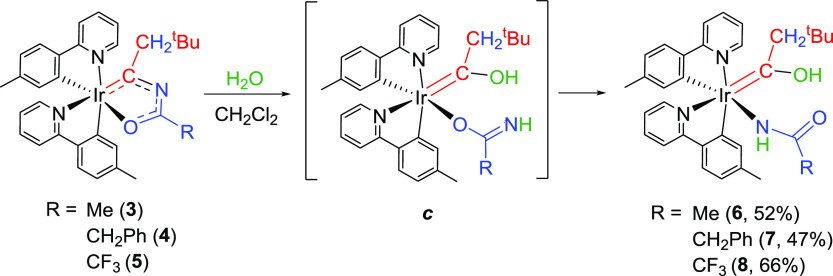
Formation of Hydroxycarbene Derivatives

The electron deficiency at the metalated carbon atom of iridaoxazole ring (C_α_) was confirmed by the hydrolysis of the Ir–C_α_ bond of **3–5** in relatively polar solvents, such as 2-methyltetrahydrofuran (2-MeTHF) and dichloromethane, at room temperature. The hydrolysis occurs with small amounts of water (>10 equiv) and generates a hydroxycarbene ligand and an amidate. The latter would initially coordinate in a κ^1^-*O*-fashion, forming intermediates ***c***. These species should exchange the donor atom of the anion to afford the hydrolysis products, complexes Ir{κ^1^-*N-*[NHC(*R*)O]}{κ^2^-*C,N*-(MeC_6_H_3_-py)}_2_{=C(CH_2_^t^Bu)OH} (R = Me (**6**), CH_2_Ph (**7**), CF_3_ (**8**)), probably through a dissociation–coordination process or alternatively via slippage of the metal center by an O–C–N path. In spite of the usual low stability of the hydroxycarbene groups, which normally undergo deprotonation to afford acyl derivatives,^19^ complexes **6–8** are surprisingly stable and were isolated as yellow solids in 40–70% yields after 24 h of reaction (Scheme 5).

**Figure 2:**
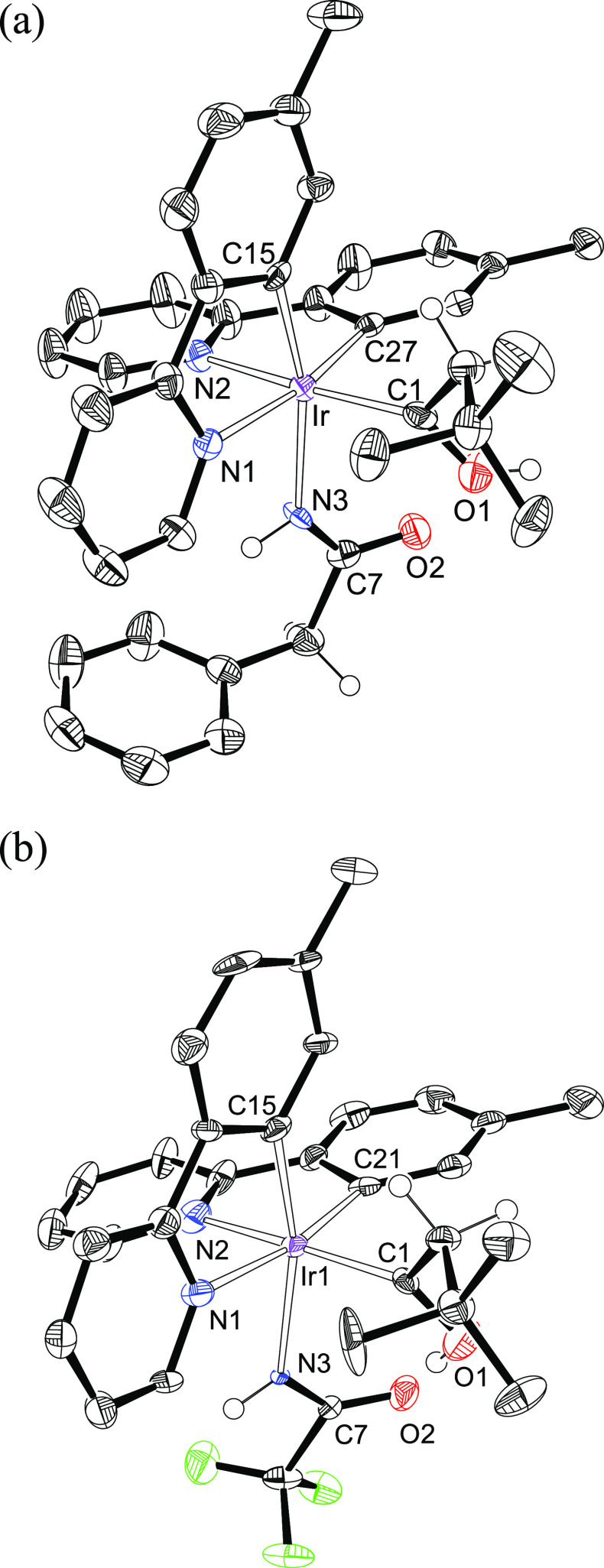
(a) ORTEP diagram of complex **7**. Only significant hydrogen atoms are shown for clarity. Selected bond lengths (Å) and angles (deg): Ir–N(1) = 2.129(4), Ir–N(2) = 2.127(4), Ir–N(3) = 2.171(3), Ir–C(1) = 1.984(5), Ir–C(15) = 1.994(4), Ir–C(27) = 2.016(4), N(3)–C(7) = 1.265(6), O(1)–C(1) = 1.323(6), O(2)–C(7) = 1.238(6), C(1)–Ir–N(2) = 169.03(17), C(27)–Ir–N(1) = 169.89(15), C(15)–Ir–N(3) = 164.99(16), O(1)–C(1)–Ir = 118.1(3), C(2)–C(1)–Ir = 128.8(3). (b) ORTEP diagram of complex **8**. Only significant hydrogen atoms are shown for clarity. Selected bond lengths (Å) and angles (deg): Ir(1)–N(1) = 2.137(8), 2.119(8); Ir(1)–N(2) = 2.121(8), 2.141(7); Ir(1)–N(3) = 2.212(5), 2.198(5); Ir(1)–C(1) = 1.971(8), 1.979(7); Ir(1)–C(15) = 2.002(6), 2.024(9); Ir(1)–C(21) = 2.020(8), 1.987(7); N(3)–C(7) = 1.267(8), 1.287(6); O(1)–C(1) = 1.302(10), 1.339(9); O(2)–C(7) = 1.199(8), 1.212(10); C(1)–Ir(1)–N(2) = 170.1(3), 171.7(3); C(21)–Ir(1)–N(1) = 170.1(3), 170.8(3); C(15)–Ir(1)–N(3) = 165.3(4), 179.0(3); O(1)–C(1)–Ir(1) = 119.8(5), 116.3(5); C(2)–C(1)–Ir(1) = 127.3(5), 129.3(5).

The formation of these unusual species was confirmed by means of the X-ray diffraction analysis structure of **7** (Figure 2a) and **8** (Figure 2b).^20^ In both cases, the hydrolysis occurs keeping the stereochemistry of the metal center. Thus, in a ligand arrangement resembling the octahedral disposition described for **2**, the hydroxycarbene ligand is disposed *trans* to the pyridyl group of a tolylpyridine chelate (C(1)–Ir–N(2) = 169.03(17)° (**7**), 170.1(3)° and 171.7(3)° (**8**)), whereas the amidate anion lies *trans* to the metalated tolyl group of the other one (N(3)–Ir–C(15) = 164.99(16)° (**7**). 165.3(4)° and 179.0(3)° (**8**)). In agreement with the hydroxycarbene nature of the monodentate C-donor ligand, angles around the C(1) atom are in the range 112–129°. The presence of a hydroxycarbene ligand in **6–8** is also strongly supported by the ^13^C{^1^H} NMR spectra of these compounds, in dichloromethane-*d*_2_, at room temperature, which show a singlet at about 230 ppm due to the C(sp^2^) atom.

**Figure 3:**
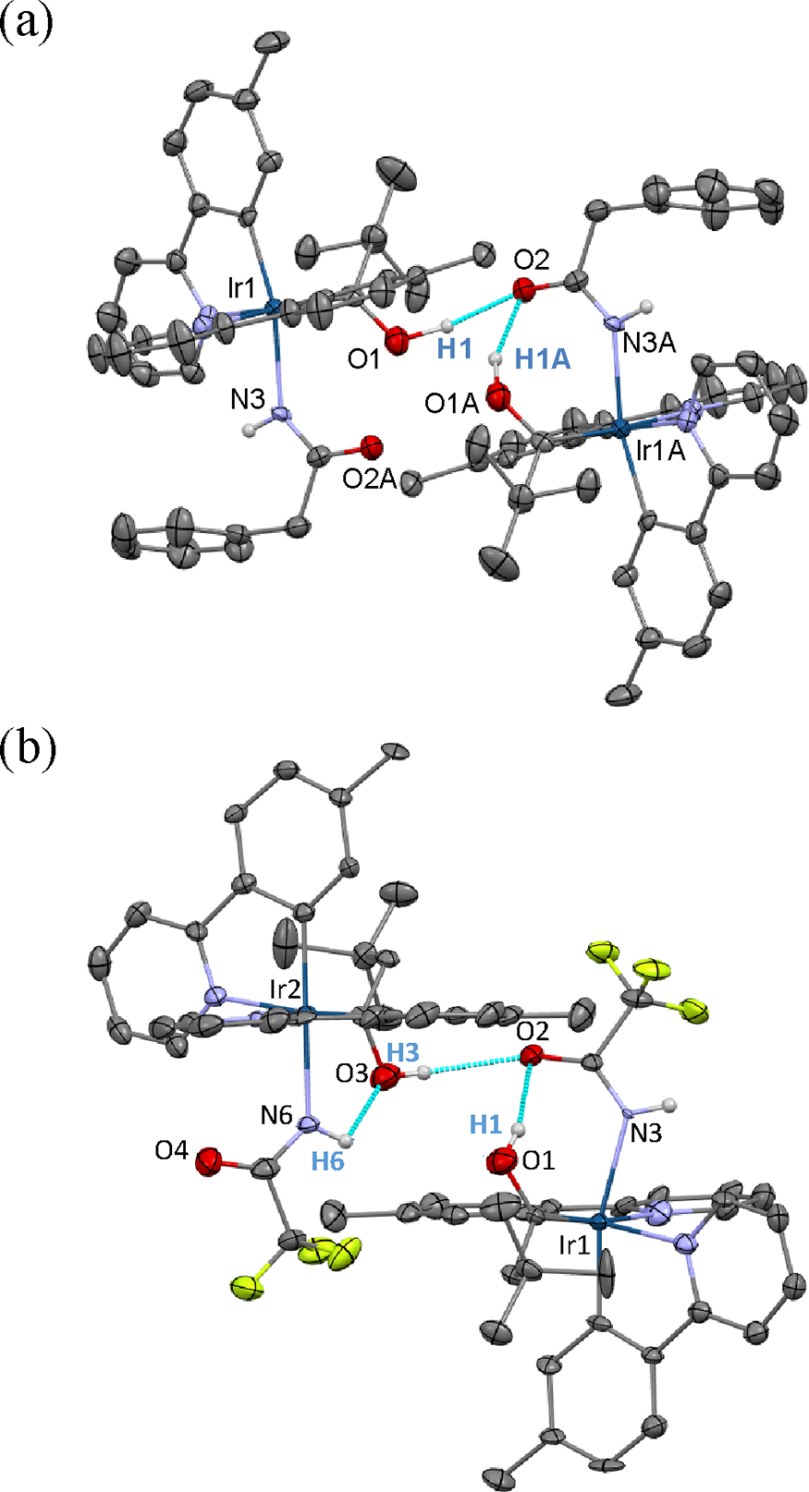
Extended view of structures of complexes **7** (a) and **8** (b).

An extended view of the structures (Figure 3) reveals that two molecules of both compounds are associated by means of hydrogen bonds to form dimers. This intermolecular interaction involves the hydrogen atom of the hydroxycarbene of one of them and the oxygen atom of the amidate group of the other. The association takes place between identical molecules in the case of **7**, while two different conformers associate in **8**. They result from the rotation of the amidate group around the Ir–N axis. In addition to this intermolecular hydrogen bond, intramolecular oxygen–hydrogen interactions are also observed, although there are significant differences between the complexes. For **7** (Figure 3a), It is only observed for the hydrogen acceptor molecule and implies to the hydrogen atom of the hydroxycarbene ligand and the oxygen atom of the amidate group. Complex **8** further displays a second intramolecular interaction, which occurs in the hydrogen donor molecule and associates the hydrogen atom of the amidate group and the oxygen atom of the hydroxycarbene ligand (Figure 3b). This second interaction is a consequence of the disposition of the NH hydrogen atom in the involved conformer, which points out the oxygen atom of the hydroxycarbene ligand. As a consequence of the interactions, the separations between the involved atoms lie in the range 2.092–2.196 Å, which are significantly shorter than the sum of the van der Waals radii of hydrogen and oxygen (r_vdw_(H) = 1.20 Å, r_vdw_(O) = 1.52 Å),^21^ whereas the angles O–H–O are close to the linearity with values between 146° and 163°.

The association is broken in dichloromethane-*d*_2_ at room temperature as is supported by ^1^H-DOSY experiments. Pulse field gradient (PFG) NMR method allows to measure diffusion rate of molecules in solution, which depends on the molecular size and the hydrodynamic volume.^22^ At 303 K, the diffusion coefficients obtained from the solutions of **7** and **8** in dichloromethane-*d*_2_ are 1.48 × 10^–9^ m^2^ s^–1^and 1.06 × 10^–10^ m^2^ s^–1^, respectively. These values allow to calculate hydrodynamic radius of 5.32 and 3.81 Å, which agrees well with those obtained from the X-ray diffraction analysis structures for the monomers, 5.81 and 3.72 Å, respectively.

The substituent at the carbon atom situated between the heteroatoms of the iridaoxazole cycle has a marked influence on the stability of the five-membered ring toward the hydrolysis. In contrast to alkyl groups, a phenyl substituent prevents the reaction with water, most probably as consequence of its hyperconjugation capacity.^23^ Thus, complex **2** does not undergo hydrolysis in opposition to compounds **3–5**.

### 2.3 ***N,C(sp^3^),C(sp^2^),O***-Tetradentate Containing Complexes

The substituent of the acetylide bridges of dimers *cis*-[Ir(μ^2^-η^2^-C≡CR){κ^2^-*C,N*-(MeC_6_H_3_-py)}_2_]_2_ (**1**) has a paramount relevance in the synthetic performance of the carbon atoms of the triple bond as building block. Treatment of the phenylacetylide derivative *cis*-[Ir(μ^2^-η^2^-C≡CPh){κ^2^-*C,N*-(MeC_6_H_3_-py)}_2_]_2_ (**1b**), in toluene, with benzamide and acetamide, under the same conditions as those previously mentioned for the formation of **2–5** leads to complexes Ir{κ^4^-*N,C,C*′*,O*-[py-MeC_6_H_3_-C(CH_2_-C_6_H_4_)NHC(*R*)O]}{κ^2^-*C,N*-(MeC_6_H_3_-py)}(R = Ph (**9**), Me (**10**)), in contrast to the *tert*-butylacetylide counterpart **1a (**Scheme 4).

**Scheme 6:**
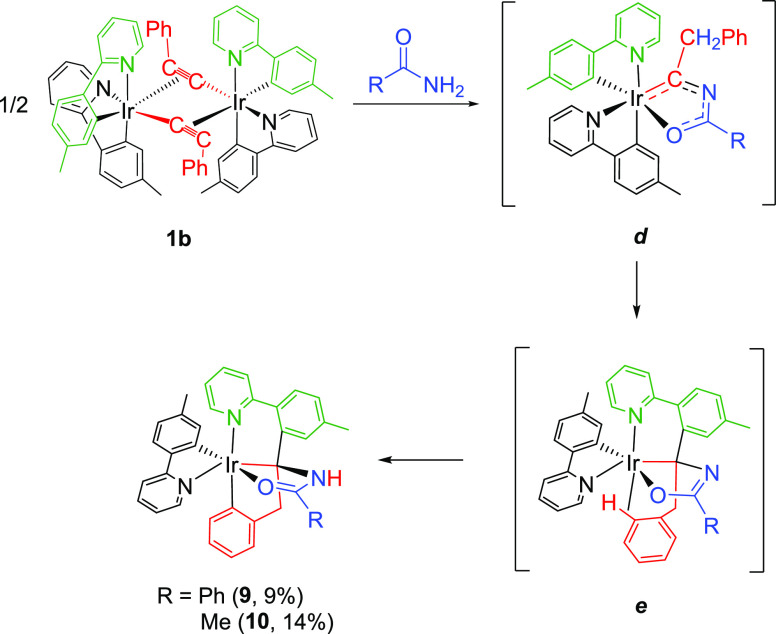
Formation of Complexes **9** and **10**

The phenylacetylide bridge of **1b** undergoes a three-component coupling involving the nitrogen atom of the amide, the C_α_-atom of the bridge, and the metalated carbon atom of one of the cyclometalated tolylpyridines (Scheme 6). The coupling can be viewed as the migratory insertion of an electron deficient Ir–C double bond of a transient iridaoxazole intermediate ***d*** into one of the *cis*-disposed cyclometalated aryl groups of the chromophores; i.e., the smaller bulky and less electron donor ability of the phenyl group in comparison to *tert*-butyl one appears to destabilize the iridaoxazole, favoring the migration of one of the metalated tolyl groups from the metal to the carbon atom of the iridaoxazole Ir–C bond. The triple coupling to give ***e***, along with the metalation of such phenyl substituent, generates an asymmetrical 6e-donor *N,C(sp^3^),C(sp^2^),O*-tetradentate ligand, which defines two different five-membered rings and other of six members. The metalation of the agostic phenyl substituent of the iridaoxazole ring of ***e***, to afford **9** and **10**, involves a hydrogen transfer from the coordinated C_*ortho*_-atom to the azole N-atom. The process could be rationalized as an intermolecular heterolytic C–H bond activation promoted by an external iridaoxazole base.

Iridium(III) emitters with nonplanar tetradentate ligands are uncommon,^24^ particularly those bearing different bidentate moieties,^25^ and in special when the donor atoms of such moieties are different in identity and nature, as is occurring in **9** and **10**. Furthermore, in contrast to our new compounds, such ligands are generated from the coordination of organic molecules previously prepared.

**Figure 4:**
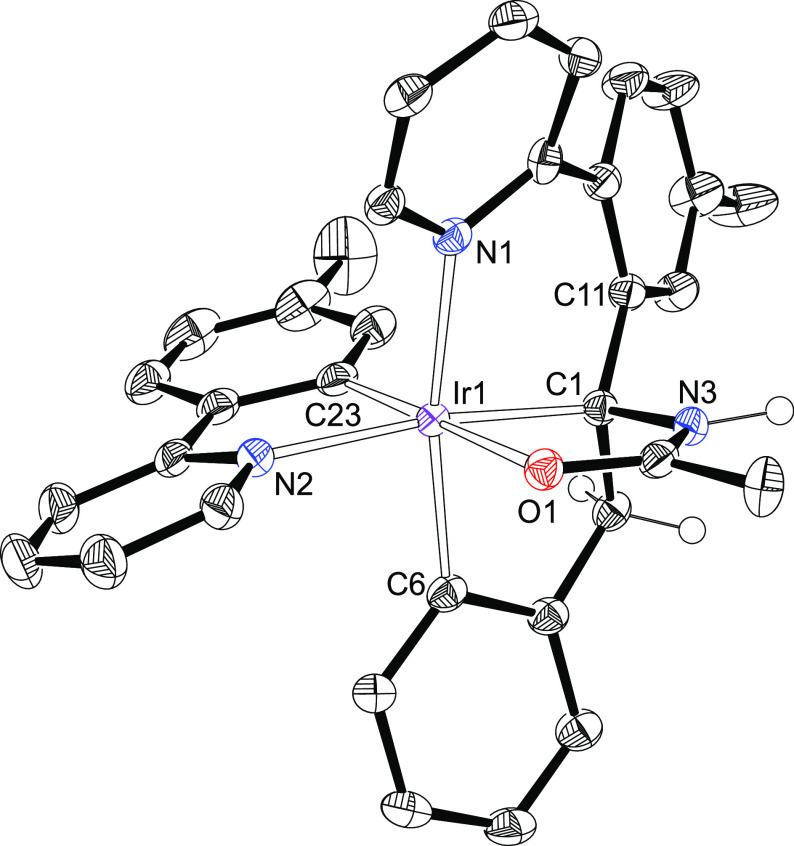
ORTEP diagram of complex **10**. Only significant hydrogen atoms are shown for clarity. Selected bond lengths (Å) and angles (deg): Ir–N(1) = 2.149(3), Ir–N(2) = 2.115(3), Ir–O(1) = 2.218(3), Ir–C(1) = 2.064(4), Ir–C(6) = 2.020(4), Ir–C(23) = 2.004(4), O(1)–C(2) = 1.264(5), N(3)–C(2) = 1.324(5), N(3)–C(1) = 1.502(5), C(1)–Ir–N(2) = 172.19(14), C(6)–Ir–N(1) = 167.47(14), C(23)–Ir–O(1) = 178.46(15).

Complexes **9** and **10** might be described as *pseudo*-tris(heterolepic) species, since bearing three different 3e donor bidentate units. Iridium(III) emitters of class [3b + 3b′ + 3b″] are certainly the most challenging because of allowing a better tuning of designed photophysical properties and because they are the most difficult of preparing.^26^ Complexes **9** and **10** were isolated as analytically pure yellow solids, in low yield (9–14%), after the corresponding purification of the reaction crude, which also contained several unidentified species, by column chromatography. The formation of their novel tetradentate moiety was confirmed by the X-ray diffraction analysis structure of **10**. Figure 4 shows a view of the molecule. The coordination around the iridium center can be idealized as an octahedron with the pyridyl and metalated phenyl groups of the tetradentate ligand mutually arranged *trans* (N(1)–Ir–C(6) = 167.47(14)°). The perpendicular plane is defined by the five-membered ring, resulting from the transient iridaoxazole ***d***, and the metalated tolylpyridine. The junction C(sp^3^) atom of the tetradentate ligand is disposed *trans* to the pyridyl group (C(1)–Ir–N(2) = 172.18(14)°), whereas the oxygen atom locates *trans* to the metalated carbon atom of the tolyl group (O(1)–Ir–C(23) = 178.46(15)°). Noticeable NMR features of **9** and **10**, in dichloromethane-*d*_2_, at room temperature are two doublets (^2^J ≈ 15.4 Hz) at about 4.1 and 3.2 ppm in the ^1^H, due to the CH_2_ group of the benzyl moiety of the tetradentate ligand, and a singlet around 54 ppm in the ^13^C{^1^H}, corresponding to the junction C(sp^3^) atom.

### 2.4 Photophysical and Electrochemical Properties of the Generated Emitters

Figures S4–S11 provide UV–vis spectra of 10^–5^ M solutions of the new complexes **2–4** and **6–10**, in 2-MeTHF or toluene, at room temperature, whereas Table 1 offers a summary of characteristic absorptions. Spectra display the typical pattern for iridium(III) species, showing the usual three energy regions: <300, 350–450, and >450 nm. According to time-dependent DFT (TD-DFT) calculations (B3LYP-D3//SDD(f)/6-31G**) in THF, the higher energy (<300 nm) bands correspond to ^1^π–π* intra- and interligand transitions, while spin-allowed charge transfers from metal-to-ligand combined with ligand-to-ligand or intraligand appear in the region of intermediate energy (350–450 nm). Formally spin-forbidden transitions, generated by a large spin–orbit coupling as a consequence of the iridium presence, are also evident after 450 nm. They are mainly HOMO-to-LUMO for **2–8** and HOMO-to-LUMO combined with HOMO-to-LUMO + 1 (≈ 60% : 30%) for **9** and **10**. In this context, it should be pointed out that the marked contribution of the iridaoxazole ring to the LUMO of **2–5**, which increases in the sequence **3** < **4** < **2** ≈ **5**, as the methyl substituent of **3** changes to CH_2_Ph, Ph, and CF_3_ in **4**, **2**, and **5**. At the time, the HOMO–LUMO gap diminishes; while this gap is about 3.9 eV for **3** (Me) and **4** (CH_2_Ph), it lies in the range 3.4–3.5 eV for **2** (Ph) and **5** (CF_3_). The HOMO–LUMO gap for the [3b + 3b + 2 m + 1 m′] complexes **6**–**8** is similar to that of **3** and **4** (Table 2). Figures S9–S17 give views of the frontier orbitals.

**Table 1:** Selected Calculated (TD-DFT in THF) and Experimental UV–Vis Absorptions for **3–4** (in toluene) and **2, 6–10** (in 2-MeTHF) and Their Major Contributions

λ exp (nm)	ε (M^–1^ cm^–1^)	exc. energy (nm)	oscilator strength (f)	transition	character of the transition
complex **2**					
250	125400	250	0.0311	HOMO – 5 → LUMO + 4 (81%)	(3b + 3b’ → 3b)
362	52200	364	0.1503	HOMO – 1 → LUMO + 2 (65%)	(Ir + 3b → 3b)
432	11700	457 (S_1_)	0.0181	HOMO – 1 → LUMO (93%)	(Ir + 3b → 3b’)
485	3000	500 (*T*_1_)	0	HOMO → LUMO (85%)	(Ir + 3b → 3b’)
complex **3**					
293	133500	296	0.0970	HOMO – 3 → LUMO + 1 (64%)	(3b + 3b’ → 3b)
372	42000	367	0.0818	HOMO – 1 → LUMO + 1 (61%)	(Ir + 3b → 3b)
400	30000	405 (S_1_)	0.0320	HOMO → LUMO (55%)	(Ir + 3b → 3b + 3b’)
				HOMO → LUMO + 2 (29%)	
470	2000	464 (*T*_1_)	0	HOMO → LUMO (64%)	(Ir + 3b → 3b)
complex **4**					
285	183000	287	0.0599	HOMO – 5 → LUMO + 2 (75%)	(3b + 3b’ → 3b)
372	54000	368	0.0841	HOMO – 1 → LUMO + 2 (85%)	(Ir + 3b → 3b)
402	34800	402 (S_1_)	0.0377	HOMO → LUMO + 1 (65%)	(Ir + 3b → 3b + 3b’)
				HOMO → LUMO (34%)	
463	6900	461 (*T*_1_)	0	HOMO → LUMO (52%)	(Ir + 3b → 3b + 3b’)
complex **6**					
278	173000	273	0.0601	HOMO – 4 → LUMO + 2 (76%)	(3b → 3b)
375	30500	384	0.0669	HOMO – 1 → LUMO (97%)	(Ir + 3b → 3b)
399	22000	394 (S_1_)	0.0316	HOMO → LUMO + 1 (92%)	(Ir + 3b → 3b)
457	7500	455 (*T*_1_)	0	HOMO → LUMO + 1 (71%)	(Ir + 3b → 3b)
complex **7**					
275	121500	274	0.0500	HOMO – 4 → LUMO + 2 (74%)	(3b → 3b)
376	23500	385	0.0631	HOMO – 1 → LUMO (96%)	(Ir + 3b → 3b)
407	14200	392 (S_1_)	0.0298	HOMO → LUMO + 1 (91%)	(Ir + 3b → 3b)
456	2600	453 (*T*_1_)	0	HOMO → LUMO + 1 (72%)	(Ir + 3b → 3b)
complex **8**					
273	182300	270	0.0302	HOMO – 2 → LUMO + 3 (58%)	(3b → 3b + 2 m)
359	38700	359	0.0430	HOMO – 1 → LUMO (89%)	(Ir + 3b → 3b)
400	22300	385 (S_1_)	0.0231	HOMO → LUMO (72%)	(Ir + 3b → 3b)
449	2800	447 (*T*_1_)	0	HOMO → LUMO + 1 (62%)	(Ir + 3b → 3b)
complex **9**					
277	1448000	273	0.0373	HOMO – 3 → LUMO + 4 (66%)	(3b + 6tt’ → 6tt’ + 3b)
395	27900	400	0.0488	HOMO → LUMO + 2 (92%)	(Ir + 3b + 6tt’ → 6tt’)
459	15900	457 (S_1_)	0.0596	HOMO → LUMO (87%)	(Ir + 3b + 6tt’ → 6tt’)
504	8500	494 (*T*_1_)	0	HOMO → LUMO (59%)	(Ir + 3b + 6tt’ → 3b + 6tt’)
				HOMO → LUMO + 1 (27%)	
complex **10**					
277	172700	272	0.1766	HOMO – 3 → LUMO + 3 (57%)	(3b + 6tt’ → 3b + 6tt’)
429	17800	431	0.0410	HOMO → LUMO + 1 (84%)	(Ir + 3b + 6tt’ → 3b)
456	15800	457 (S_1_)	0.0564	HOMO → LUMO (86%)	(Ir + 3b + 6tt’ → 6tt’)
502	9500	494 (*T*_1_)	0	HOMO → LUMO (56%)	(Ir + 3b + 6tt’ → 3b + 6tt’)
				HOMO → LUMO + 1 (33%)	

**Table 2:** Electrochemical and DFT Molecular Orbitals Energy Data for **2–10**

		obs (eV)	calcd (eV)		
complex	*E*_1/2_^ox^ vs Fc/Fc^+^ (V)	HOMO^a^	HOMO	LUMO	HLG^b^
**2**	0.44, 1.05	–5.24	–5.18	–1.75	3.43
**3**			–5.18	–1.30	3.88
**4**			–5.18	–1.36	3.82
**5**			–5.36	–1.82	3.54
**6**	0.37, 0.86	–5.17	–5.08	–1.24	3.84
**7**	0.37, 0.87	–5.17	–5.08	–1.23	3.85
**8**	0.50, 0.83	–5.30	–5.29	–1.34	3.95
**9**	0.01, 0.79	–4.81	–4.65	–1.26	3.39
**10**	0.00, 0.73	–4.80	–4.64	–1.25	3.39

a HOMO = −[*E*_1/2_^ox^ vs Fc/Fc^+^ + 4.8] eV.

b HGL = LUMO – HOMO.

The HOMO energy levels DFT-calculated for **2** and **6–10** nicely agree with those experimentally obtained from the electrochemical study of these compounds. Figure S22 shows the voltammograms, which were measured in dichloromethane, under argon, using [Bu_4_N]PF_6_ as supporting electrolyte (0.1 M). All compounds display reversible oxidations from Ir(III) to Ir(IV) and from Ir(IV) to Ir(V) between 0.00 and 1.05 V (Table 2). Reductions were not detected between −1.5 and 1.5 V.

**Figure 5:**
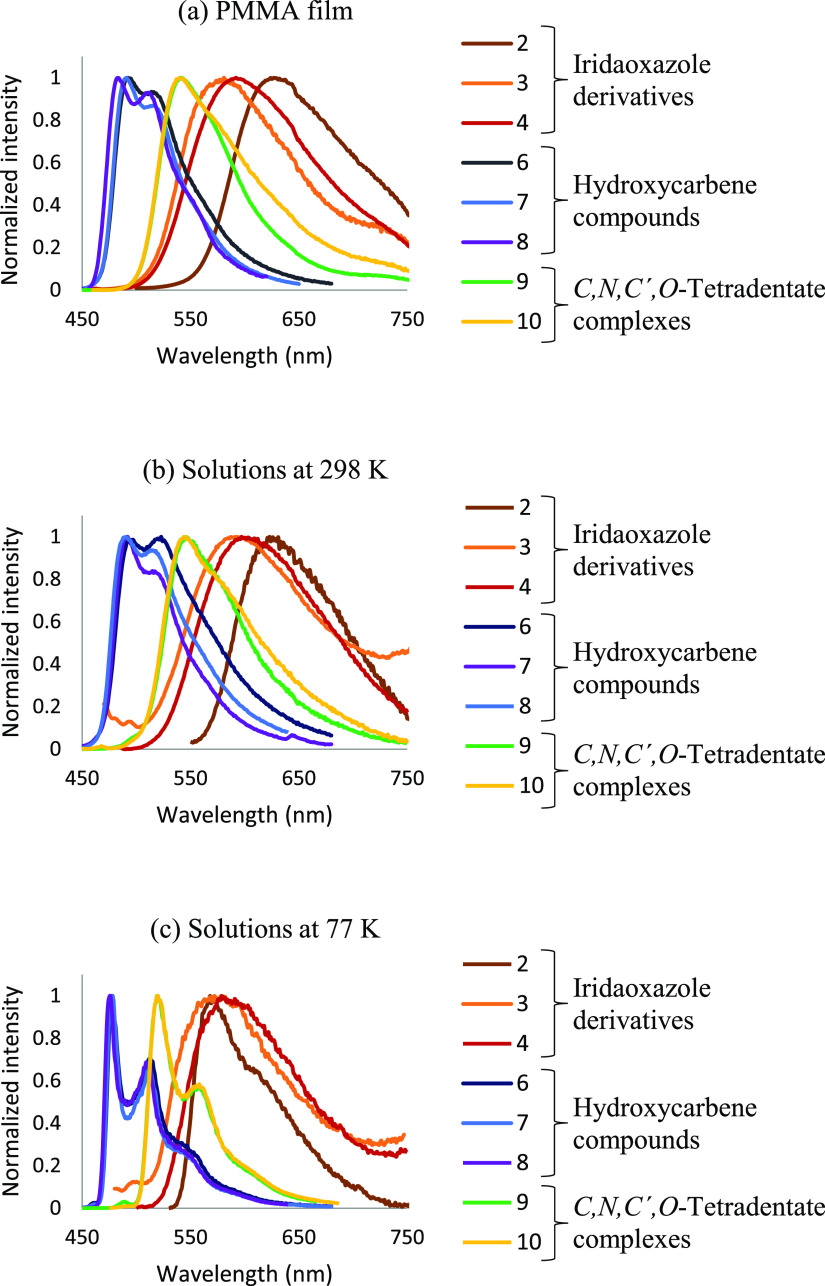
(a) Emission spectra of **2–4** and **6–10** in 5 wt % PMMA films at 298 K. (b) Emission spectra of **2**, **6–10** in 2-MeTHF and **3–4** in toluene at 298 K. (c) Emission spectra of **2**, **6–10** in 2-MeTHF and **3–4** in toluene at 77 K.

Table 3 summarizes features of emissions, upon photoexcitation, of iridaoxazole complexes **2–4**, hydroxycarbene compounds **6–8**, and tetradentate derivatives **9** and **10**. The measurements were carried out in a doped poly-(methyl methacrylate) (PMMA) film at 5 wt %, at room temperature, and 2-MeTHF (**2** and **6–10**) or toluene (**3** and **4**) at room temperature and at 77 K. Figure 5 collects the spectra of the three classes of emitters recorded under the above mentioned experimental conditions. Emissions take place from the respective *T*_1_ excited states, as is supported by the experimental wavelength values, which are consistent with the difference in energy, calculated in THF, between the optimized triplet states *T*_1_ and the singlet states *S*_0_.

**Table 3:** Photophysical Data of Complexes **2–4** and **6–10**

calcd λ_em_ (nm)	media (T/K)	λ_em_ (nm)	τ (μs)	Φ	*k*_*r*_^a^ (s^–1^)	*k*_*nr*_^a^ (s^–1^)	*k*_*r*_/*k*_*nr*_
complex **2**							
627	PMMA (298)	627	2.5	0.04	1.6 × 10^4^	3.8 × 10^5^	0.04
	2-MeTHF (298)	628	0.9	0.01	1.1 × 10^4^	1.1 × 10^6^	0.01
	2-MeTHF (77)	600, 639	1.5				
complex **3**							
536	PMMA (298)	581	1.4	0.08	5.7 × 10^4^	6.6 × 10^5^	0.09
	Toluene (298)	590	0.4	0.07	1.8 × 10^5^	2.3 × 10^6^	0.08
	Toluene (77)	578	4.0				
complex **4**							
553	PMMA (298)	592	0.9	0.08	8.9 × 10^4^	1.0 × 10^6^	0.09
	Toluene (298)	600	0.4	0.06	1.5 × 10^5^	2.4 × 10^6^	0.06
	Toluene (77)	584	3.8				
complex **6**							
500	PMMA (298)	492, 516	1.4	0.29	2.1 × 10^5^	5.1 × 10^5^	0.41
	2-MeTHF (298)	497, 520	1.2	0.10	8.3 × 10^4^	7.5 × 10^5^	0.11
	2-MeTHF (77)	478, 513, 555	4.3				
complex **7**							
504	PMMA (298)	490, 515	1.5	0.44	2.9 × 10^5^	3.7 × 10^5^	0.78
	2-MeTHF (298)	492, 515	1.2	0.12	1.0 × 10^5^	7.3 × 10^5^	0.14
	2-MeTHF (77)	477, 512, 542	4.6				
complex **8**							
440	PMMA (298)	483, 511, 553	1.5	0.11	7.3 × 10^4^	5.9 × 10^5^	0.12
	2-MeTHF (298)	490, 515	1.8	0.07	3.9 × 10^4^	5.2 × 10^5^	0.08
	2-MeTHF (77)	476, 509, 548	5.0				
complex **9**							
542	PMMA (298)	540	0.7	0.50	7.1 × 10^5^	7.1 × 10^5^	1.00
	2-MeTHF (298)	544	0.2	0.04	2.0 × 10^5^	4.8 × 10^6^	0.04
	2-MeTHF (77)	520, 556	7.5				
complex **10**							
540	PMMA (298)	546	1.5	0.45	3.0 × 10^5^	3.7 × 10^5^	0.81
	2-MeTHF (298)	546	0.8	0.12	1.5 × 10^5^	1.1 × 10^6^	0.14
	2-MeTHF (77)	523, 558	9.0				

a Calculated according to *k_r_=* Φ/τ and *k_nr_=* (1 – Φ)/τ.

Iridaoxazoles complexes **2–4** are poor orange emitters (578–639 nm), which display low quantum yields (<10). Ring opening hydrolysis of iridaoxazole results in a blue shift of the emission and a significant increase of the quantum yields. Thus, the hydroxycarbene compounds **6–8** are green emitters (476–556 nm), which beam with moderated efficiency; particularly in the case of **7**. The quantum yields of latter reach values of 0.44 in PMMA and 0.17 in 2-MeTHF. By their part, the tetradentate derivatives **9** and **10** are yellow emitters (520–558 nm), which show quantum yields in PMMA higher than those of **6**–**8**, around 0.50. Like for the hydroxycarbene compounds, the efficiency of both drops in solution. This appears to be due to a significant rise of the nonradiative rate constants in solution, suggesting a strong energy dissipation through mechanical processes. The lifetimes are short with values in the range 0.2–9.0 μs.

## 3 Concluding Remarks

This study has revealed that the alkynyl ligands of dimers *cis*-[Ir(μ^2^-η^2^-C≡CR){κ^2^-*C,N*-(MeC_6_H_3_-py)}_2_]_2_ are building blocks to build iridaoxazole rings, hydroxycarbene moieties, and novel 6e-donor *N,C(sp^3^),C(sp^2^),O*-tetradentate ligands.

Only one class of monocyclic organometallic metalaheteroaromatic compounds, bearing two main-group heteroatoms in the ring, was known as far, osmaoxazoles; their formation starting from an amidate and an alkylidyne ligand was reported some months ago. A new family of metalaoxazoles, iridaoxazoles, has been now generated using an amide and an alkynyl ligand instead of an amidate and the alkylidyne unit. It is further demonstrated that the L_n_M fragment of the five-membered ring has a marked influence in the aromaticity degree of the cycle and its stability toward the hydrolysis. The results reported here point out that L_n_M fragments with a low back bonding ability create a significant electron deficiency in the carbon atom of the M–C bond that reduces the aromaticity of the five-membered ring and polarizes the adjacent C–N bond. The increased difference in charge between the atoms of such bond enhances their affinity by the water molecule, which promotes the C–N rupture to form amidate–iridium(III)–hydroxycarbene derivatives. The substituents at the carbon atoms of the iridaoxazole have also a paramount importance in the stability of the five-membered ring. In contrast to alkyl groups, a phenyl substituent situated at the carbon atom between the heteroatoms of cycle prevents the hydrolysis.

The iridaoxazole ring is the starting point not only of the hydroxycarbene moieties but also of *N,C(sp^3^),C(sp^2^),O*-tetradentate ligands. A significant bulkiness reduction in the CH_2_R group, generated in the process of the five-membered ring built, unprotects the metalated carbon atom toward the attack of one of the tolyl groups coordinated to the iridium centers in the starting dimers. The migration is the seed for the tetradentate ligands when the R is a phenyl group, since the latter is able to undergo a subsequent *ortho*-CH bond activation. These tetradentate ligands are therefore the result of a triple coupling (an alkynyl ligand, an amide, and a coordinated aryl group) and a C–H bond activation at the metal coordination sphere. Furthermore, they point out the decisive role of the akynyl substituent of the starting dimers in the nature of the C–C triple bond as building block.

The compounds prepared by these novel procedures represent novel families of heteroleptic iridium(III) phosphorescent emitters in the orange-green region of the emission spectrum, which display quantum yields between low and moderate and short lifetimes.

In summary, the use of dimers *cis*-[Ir(μ^2^-η^2^-C≡CR){κ^2^-*C,N*-(MeC_6_H_3_-py)}_2_]_2_ as starting materials allows to develop original synthetic procedures, which leads to classes of iridium(III) phosphorescent emitters different to those previously known.

## 4 Experimental Section

### 4.1 General Information

All reactions were carried out under argon with dried solvents and using Schlenk tube techniques. Instrumental methods are given in the Supporting Information. In the NMR spectra, chemical shifts (expressed in ppm) are referenced to residual solvent peaks and coupling constants (*J*) are given in hertz. Signals were assigned using also bidimensional NMR spectra (^1^H–^1^H COSY, ^1^H–^13^C{^1^H} HSQC and ^1^H–^13^C{^1^H} HMBC).

#### 4.1.1 Preparation of Ir{κ^2^-*C,O*-[C(CH_2_^t^Bu)NC(*Ph*)O]}{κ^2^-*C,N*-(MeC_6_H_3_-py)}_2_ (**2**)

To a suspension of **1a** (300 mg, 0.246 mmol) in toluene (20 mL) placed in a Schlenk flask equipped with a PTFE stopcock, benzamide (60 mg, 0.492 mmol) was added. The mixture was hold during 24 h at 120 °C. The red solution was cooled at room temperature and evaporated to dryness. The crude was purified by silica column chromatography (deactivated with NEt_3_) using toluene as eluent to get a red solid, which was washed with pentane (3 × 5 mL) and dried to vacuum (164 mg, 46%). Anal. Calcd. for C_37_H_36_IrN_3_O: C, 60.80; H, 4.96; N, 5.75. Found: C, 60.41; H, 4.81; N, 5.54. HRMS (electrospray, *m/z*): Calcd. for C_37_H_37_IrN_3_O [M + H]^+^: 732.2562, found: 732.2587. IR (cm^–1^): *v*(CO) 1600 (m), *v*(C=N) 1585 (m). ^1^H NMR (400 MHz, CD_2_Cl_2_, 298 K): δ 8.46 (dd, ^3^*J*_H-H_ = 8.2, ^4^*J*_H-H_ = 1.2, 2H, CH Ph), 8.13 (ddd, ^3^*J*_H-H_*=* 5.5, ^4^*J*_H-H_ = 1.7, ^5^*J*_H-H_ = 0.9, 1H, CH py), 7.90 (d, ^3^*J*_H-H_ = 8.3, 1H, CH py), 7.82 (d, ^3^*J*_H-H_ = 8.3, 1H, CH py), 7.76 (ddd, ^3^*J*_H-H_ = 8.3; 7.3, ^4^*J*_H-H_ = 1.7, 1H, CH py), 7.62–7.50 (m, 4H, 2H CH MeC_6_H_3_-py + CH py + CH Ph), 7.49–7.41 (m, 2H, CH Ph), 7.19–7.08 (m, 2H, CH py), 6.92 (s, 1H, CH MeC_6_H_3_-py), 6.88–6.80 (m, 2H, CH MeC_6_H_3_-py + CH py), 6.72 (dd, ^3^*J*_H-H_ = 8.2, ^4^*J*_H-H_ = 1.2, 1H, CH MeC_6_H_3_-py), 6.65 (s, 1H, CH MeC_6_H_3_-py), 2.89 (d, ^2^*J*_H-H_ = 14.6, 1H, CH_2_-^t^Bu), 2.43 (d, ^2^*J*_H-H_ = 14.6, 1H, CH_2_-^t^Bu), 2.29, 2.10 (both s, 3H each, CH_3_ MeC_6_H_3_-py), 0.75 (s, 9H, ^t^Bu). ^13^C{^1^H}-APT NMR (101 MHz, CD_2_Cl_2_, 253 K): δ 267.1 (s, Ir–C=N), 190.3 (s, Ir–O=C), 166.0 (s, N–C py), 165.0 (s, N–C py), 158.3 (s, C MeC_6_H_3_-py), 151.8 (s, C MeC_6_H_3_-py), 149.1 (s, CH py), 146.6 (s, CH py), 141.6 (s, C MeC_6_H_3_-py), 141.2 (s, C MeC_6_H_3_-py), 140.8 (s, C MeC_6_H_3_-py), 140.0 (s, C MeC_6_H_3_-py), 139.1 (s, CH MeC_6_H_3_-py), 137.8 (s, py), 137.6 (s, CH MeC_6_H_3_-py), 137.3 (s, CH py), 134.1 (s, C Ph), 132.8 (s, CH Ph), 131.5 (s, 2C, CH Ph), 128.6 (s, 2C, CH Ph), 124.2 (s, CH MeC_6_H_3_-py tol), 124.0 (s, CH MeC_6_H_3_-py), 122.6 (s, CH MeC_6_H_3_-py), 122.4 (s, CH py), 122.2 (s, CH MeC_6_H_3_-py), 121.8 (s, CH py), 119.0 (s, CH py), 118.7 (s, CH py), 62.1 (s, CH_2_), 32.9 (s, C_q_-^t^Bu), 30.3 (s, CH_3_^t^Bu), 21.9, 21.8 (both s, CH_3_ MeC_6_H_3_-py).

#### 4.1.2 Preparation of Ir{κ^2^-*C,O*-[C(CH_2_^t^Bu)NC(CH_3_)O]}{κ^2^-*C,N*-(MeC_6_H_3_-py)}_2_ (**3**)

To a suspension of **1a** (300 mg, 0.246 mmol) in toluene (15 mL) placed in a Schlenk flask equipped with a PTFE stopcock, acetamide (44 mg, 0.745 mmol) was added. The mixture was hold during 24 h at 120 °C. After that time, the solution was cooled at room temperature, filtered through Celite, and evaporated to dryness. The crude was purified by column chromatography (basic Al_2_O_3_, activity grade V) using toluene as eluent to eliminate an impurity and then using acetonitrile to get an orange solid (105 mg, 32%). Anal. Calcd for C_32_H_34_IrN_3_O: C, 57.46; H, 5.12; N, 6.28. Found: C, 57.48; H, 5.46; N, 6.32. HRMS (electrospray, *m/z*): Calcd for C_32_H_35_IrN_3_O [M + H]: 670.2392; found: 670.2395. IR (cm^–1^): *v*(CO) 1600 (m), *v*(C=N) 1589 (m). ^1^H NMR (400 MHz, CD_2_Cl_2_, 253 K): δ 8.14 (d, ^3^*J*_H-H_*=* 5.5, 1H, CH py), 7.90 (d, ^3^*J*_H-H_*=* 8.3, 1H, CH py), 7.82–7.75 (m, 2H, CH py), 7.57 (d, ^3^*J*_H-H_*=* 8.3, 2H, CH py, CH MeC_6_H_3_-py), 7.50 (d, ^3^*J*_H-H_*=* 7.9, 1H, CH MeC_6_H_3_-py), 7.23 (ddd, ^3^*J*_H-H_*=* 7.0; 5.5, ^4^*J*_H-H_*=* 1.3, 1H, CH py), 7.02 (d, ^3^*J*_H-H_*=* 5.5, 1H, CH py), 6.88–6.86 (m, 2H, CH MeC_6_H_3_-py), 6.80 (ddd, ^3^*J*_H-H_*=* 7.0; 5.5, ^4^*J*_H-H_*=* 1.2, 1H, CH py), 6.69 (d, ^3^*J*_H-H_*=* 7.9, 1H, CH MeC_6_H_3_-py), 6.61 (s, 1H, CH MeC_6_H_3_-py), 3.00 (d, *^2^J*_H-H_ = 13.3, 1H, C*H*_2_-^t^Bu), 2.60 (s, 3H, CH_3_ acetamide), 2.33 (s, 3H, CH_3_ MeC_6_H_3_-py), 2.23 (d, *^2^J*_H-H_ = 13.3, 1H, C*H*_2_-^t^Bu) 2.08 (s, 3H, CH_3_ MeC_6_H_3_-py), 0.58 (s, 9H, ^t^Bu). ^13^C{^1^H} NMR (101 MHz, CD_2_Cl_2_, 253 K): δ 268.7 (s, Ir–C=N), 197.5 (s, Ir–O=C), 166.1 (s, N—C py), 164.9 (s, N—C py), 158.1 (s, C MeC_6_H_3_-py), 152.0 (s, C MeC_6_H_3_-py), 149.0 (s, CH py), 146.4 (s, CH py), 141.6 (s, C MeC_6_H_3_-py), 141.0 (s, C MeC_6_H_3_-py), 140.8 (s, C MeC_6_H_3_-py), 140.0 (s, CH MeC_6_H_3_-py), 139.0 (s, CH MeC_6_H_3_-py), 137.9 (s, CH py), 137.6 (s, CH MeC_6_H_3_-py), 137.2 (s, CH py), 124.2 (s, CH MeC_6_H_3_-py), 124.0 (s, CH MeC_6_H_3_-py), 122.6 (s, CH py), 122.4 (s, CH MeC_6_H_3_-py), 122.2 (s, CH MeC_6_H_3_-py), 121.8 (s, CH py), 119.1 (s, CH py), 118.7 (s, CH py), 61.2 (s, CH_2_), 32.9 (s, C_q_-^t^Bu), 30.2 (s, CH_3_^t^Bu), 23.7 (s, CH_3_ acetamide), 21.8, 21.7 (both s, CH_3_ MeC_6_H_3_-py).

#### 4.1.3 Preparation of Ir{κ^2^-*C,O*-[C(CH_2_^t^Bu)NC(CH_2_Ph)O]}{κ^2^-*C,N*-(MeC_6_H_3_-py)}_2_ (**4**)

To a suspension of **1a** (300 mg, 0.246 mmol) in toluene (15 mL) placed in a Schlenk flask equipped with a PTFE stopcock, phenylacetamide (66.5 mg, 0.492 mmol) was added. The mixture was hold during 24 h at 120 °C. The resulting orange solution was cooled at room temperature, filtered through Celite, and evaporated to dryness. The crude was purified by silica column chromatographic (deactivated with NEt_3_) using pentane:dichloromethane (1:2) as eluent to get an orange solid (196 mg, 53%). Anal. Calcd. for C_38_H_38_IrN_3_O: C, 61.27; H, 5.14; N, 5.64. Found: C, 60.90; H, 5.49; N, 5.75. HRMS (electrospray, *m/z*): Calcd. for C_38_H_39_IrN_3_O [M + H]^+^: 746.2719, found: 746.2736. IR (cm^–1^): *v*(CO) 1601 (m), *v*(C=N) 1589 (m). ^1^H NMR (400 MHz, CD_2_Cl_2_, 298 K): δ 7.83 (d, *^3^J*_H-H_*=* 8.2, 1H, CH py), 7.78 (d, ^3^*J*_H-H_*=* 8.2, 1H, CH py), 7.69 (dd, ^3^*J*_H-H_*=*^3^*J’*_H-H_ = 7.5, 1H, CH py), 7.62–7.51 (m, 3H, 2H CH py + CH MeC_6_H_3_-py), 7.46 (d, ^3^*J*_H-H_*=* 7.9, 1H, MeC_6_H_3_-py), 7.35 (d, ^3^*J*_H-H_*=* 7.1, 2H, CH Ph), 7.33–7.21 (m, 3H, CH Ph) 6.96 (d, ^3^*J*_H-H_*=* 5.4, 1H, CH py), 6.94–6.85 (m, 3H, 2H CH MeC_6_H_3_-py + CH py), 6.77 (dd, ^3^*J*_H-H_ = ^3^*J’*_H-H_ = 6.4, 1H, CH py), 6.68 (d, ^3^*J*_H-H_ = 7.9, 1H, CH MeC_6_H_3_-py), 6.62 (s, 1H, CH MeC_6_H_3_-py), 4.34 (d, ^2^*J*_H-H_ = 13.2, 1H, C*H*_2_Ph), 3.97 (d, ^2^*J*_H-H_ = 13.2, 1H, C*H*_2_Ph), 2.98 (d, ^2^*J*_H-H_ = 13.6, 1H, C*H*_2_-^t^Bu), 2.33 (s, 3H, CH_3_ MeC_6_H_3_-py), 2.25 (d, ^2^*J*_H-H_ = 13.6, 1H, C*H*_2_-^t^Bu), 2.07 (s, 3H, CH_3_ MeC_6_H_3_-py), 0.57 (s, 9H, ^t^Bu). ^13^C{^1^H}-APT NMR (101 MHz, CD_2_Cl_2_, 253 K): δ 270.3 (s, Ir—C=N), 198.3 (s, Ir—O=C), 165.9 (s, N—C py), 164.8 (s, N—C py), 157.9 (s, C MeC_6_H_3_-py), 151.6 (s, C MeC_6_H_3_-py), 148.8 (s, CH py), 146.5 (s, CH py), 141.6 (s, C MeC_6_H_3_-py), 141.0 (s, C MeC_6_H_3_-py), 140.8 (s, C MeC_6_H_3_-py), 140.0 (s, C MeC_6_H_3_-py), 139.3 (s, CH MeC_6_H_3_-py), 137.9 (s, C Ph), 137.7 (s, CH py), 137.6 (s, CH MeC_6_H_3_-py), 137.3 (s, CH py), 129.6 (s, 2C, CH Ph), 128.7 (s, 2C, CH Ph), 126.9 (s, CH Ph), 124.2 (s, CH MeC_6_H_3_-py), 123.9 (s, CH MeC_6_H_3_-py), 122.7 (s, CH MeC_6_H_3_-py), 122.2 (s, CH MeC_6_H_3_-py), 122.1 (s, CH py), 121.8 (s, CH py), 119.0 (s, CH py), 118.6 (s, CH py), 61.5 (s, *C*H_2_-^*t*^Bu), 43.7 (s, *C*H_2_Ph), 33.1 (s, C_q_-^t^Bu), 30.2 (s, CH_3_-^t^Bu), 21.8, 21.7 (both s, CH_3_ MeC_6_H_3_-py).

#### 4.1.4 Preparation of Ir{κ^2^-*C,O*-[C(CH_2_^t^Bu)NC(CF_3_)O]}{κ^2^-*C,N*-(MeC_6_H_3_-py)}_2_ (**5**)

To a suspension of **1a** (300 mg, 0.246 mmol) in toluene (20 mL) placed in a Schlenk flask equipped with a PTFE stopcock, trifluoroacetamide (55.6 mg, 0.492 mmol) was added. The mixture was hold during 24 h at 120 °C. The resulting red solution was cooled at room temperature, filtered through Celite, and evaporated to dryness. The addition of 10 mL of pentane to the crude causes the precipitation of a reddish pink solid, which was washed with pentane (5 × 5 mL) and evaporated to dryness (212 mg, 60%). Anal. Calcd. for C_32_H_31_F_3_IrN_3_O: C, 53.17; H, 4.32; N, 5.81. Found: C, 53.47; H, 4,64; N, 6.03. HRMS (electrospray, *m/z*): Calcd. for C_32_H_32_F_3_IrN_3_O [M + H]^+^: 724.2123, found: 724.2126. IR (cm^–1^): *v*(CO) 1603 (m), *v*(C=N) 1588 (m), *v*(CF_3_) 1175, 1144 (s). ^1^H NMR (400 MHz, CD_2_Cl_2_, 298 K): δ 8.04 (ddd, ^3^*J*_H-H_*=* 5.6, ^4^*J*_H-H_ = 1.7, ^5^*J*_H-H_ = 1.0, 1H, CH py), 7.93 (ddd, ^3^*J*_H-H_*=* 8.4, ^4^*J*_H-H_ = ^4^*J*_H-H_ = 1.3, 1H, CH py), 7.87–7.78 (m, 2H, CH py), 7.63–7.57 (m, 2H, CH MeC_6_H_3_-py + CH py), 7.53 (d, ^3^*J*_H-H_ = 7.9, 1H, CH MeC_6_H_3_-py), 7.26 (ddd, ^3^*J*_H-H_ = 7.2, 5.6, ^4^*J*_H-H_ = 1.3, 1H, CH py), 6.98 (ddd, ^3^*J*_H-H_ = 5.6, ^4^*J*_H-H_ = 1.7, ^5^*J*_H-H_ = 1.0, 1H, CH py), 6.91 (ddd, ^3^*J*_H-H_ = 7.9, ^4^*J*_H-H_ = 1.8, ^5^*J*_H-H_ = 0.8, 1H, CH MeC_6_H_3_-py), 6.85 (ddd, ^3^*J*_H-H_ = 7.2, 5.6, ^4^*J*_H-H_ = 1.3, 1H, CH py), 6.76 (ddd, ^3^*J*_H-H_ = 7.9, ^4^*J*_H-H_ = 1.8, ^5^*J*_H-H_ = 0.8, 1H, CH MeC_6_H_3_-py), 6.70 (s, 1H, CH MeC_6_H_3_-py), 6.63 (s, 1H, CH MeC_6_H_3_-py), 3.18 (d, *^2^J*_H-H′_ = 13.2, 1H, C*H*_2_-^t^Bu), 2.31 (s, 3H, CH_3_ MeC_6_H_3_-py), 2.20 (d, ^2^*J*_H-H′_ = 13.2, 1H, C*H*_2_-^t^Bu), 2.10 (s, 3H, CH_3_ MeC_6_H_3_-py), 0.62 (s, 9H, ^t^Bu). ^13^C{^1^H}-APT NMR plus HSQC and HMBC (101 MHz, CD_2_Cl_2_, 253 K): δ 280.4 (s, Ir–C=N), 179.5 (q, ^2^*J*_C-F_ = 34.6, Ir—O*=C*CF_3_), 165.7 (s, N—C py), 164.4(s, N—C py), 155.7 (s, C MeC_6_H_3_-py), 149.4 (s, CH py), 148.0 (s, C MeC_6_H_3_-py), 146.3 (s, CH py), 141.5 (s, 2C MeC_6_H_3_-py), 140.8 (s, C MeC_6_H_3_-py), 140.6 (s, C MeC_6_H_3_-py), 139.3 (s, tol), 138.8 (s, py), 138.0 (s, CH py), 137.4 (s, CH MeC_6_H_3_-py), 124.4 (s, CH MeC_6_H_3_-py), 124.1 (s, CH MeC_6_H_3_-py), 123.7 (s, CH MeC_6_H_3_-py), 123.3 (s, CH MeC_6_H_3_-py), 123.1 (s, CH py), 122.1 (s, CH py), 119.6 (s, CH py), 119.0 (s, CH py), 117.7 (q, ^1^*J*_C-F_ = 281.4, C-*C*F_3_), 63.6 (s, CH_2_), 33.5 (s, C ^t^Bu), 30.3 (s, CH_3_-^t^Bu), 21.8 (s, CH_3_ MeC_6_H_3_-py), 21.7 (s, *C*H_3_ MeC_6_H_3_-py). ^19^F NMR (376.5 MHz, CD_2_Cl_2_, 298 K): −71.9 (s, C*F*_3_).

#### 4.1.5 Preparation of Ir{κ^1^-*N-*[NHC(CH_3_)O]}{κ^2^-*C,N*-(MeC_6_H_3_-py)}_2_{=C(CH_2_^t^Bu)OH} (**6**)

To a solution of complex **3** (50 mg, 0.075 mmol) in dichloromethane (5 mL), deoxygenated water (15 μL, 0.833 mmol) was added. The solution was stirring at room temperature for 24 h and then evaporated to dryness. The crude was washed with pentane (3 × 5 mL) affording a yellow solid (27 mg, 52%). Anal. Calcd for C_32_H_36_IrN_3_O_2_: C, 55.96; H, 5.28; N, 6.12. Found: C, 56.14; H, 5.02; N, 5.98. HRMS (electrospray, *m/z*): Calcd for C_30_H_31_IrN_2_O [M - acetamide]: 628.2060; found: 628.2031. IR (cm^–1^): *v*(OH) 3347, *v*(NH) 3033, *v*(CO) 1587 (s). ^1^H NMR (300 MHz, CD_2_Cl_2_, 298 K): δ 13.18 (s br, 1H, OH), 8.94 (ddd, ^3^*J*_H-H_*=* 5.5, ^4^*J*_H-H_*=* 1.7, ^5^*J*_H-H_*=* 0.9, 1H, CH py; and s br, 1H, NH), 7.97 (d, ^3^*J*_H-H_*=* 8.2, 1H, CH py), 7.85 (ddd, ^3^*J*_H-H_*=* 8.2; 7.4, ^4^*J*_H-H_*=* 1.7, 1H, CH py), 7.71 (d, ^3^*J*_H-H_*=* 8.2, 1H, CH py), 7.56–7.47 (m, 3H, CH py, CH MeC_6_H_3_-py), 7.43 (s, 1H, CH MeC_6_H_3_-py), 7.31 (ddd, ^3^*J*_H-H_*=* 7.4; 5.5, ^4^*J*_H-H_*=* 1.3, 1H, CH py), 7.08 (ddd, ^3^*J*_H-H_*=* 5.6, ^4^*J*_H-H_*=* 1.5, ^5^*J*_H-H_*=* 0.7, 1H, CH py), 6.88 (dd, ^3^*J*_H-H_*=* 7.9, ^4^*J*_H-H_*=* 1.2, 1H, CH MeC_6_H_3_-py), 6.73 (ddd, ^3^*J*_H-H_*=* 7.1; 5.6, ^4^*J*_H-H_*=* 1.3, 1H, CH py), 6.64 (dd, ^3^*J*_H-H_*=* 7.9, ^4^*J*_H-H_*=* 1.2, 1H, CH MeC_6_H_3_-py), 6.37 (s, 1H, CH MeC_6_H_3_-py), 2.42 (s, 3H, CH_3_), 1.99 (s, 3H, CH_3_), 1.87 (AB spin system, Δν = 33, *J*_A-B_*=* 15.0, 2H, CH_2_), 1.61 (s, 3H, CH_3_), 0.59 (s, 9H, ^t^Bu). ^13^C{^1^H}NMR (75 MHz, CD_2_Cl_2_, 298 K): δ 229.0 (s, Ir=C), 179.5 (s, C=O), 166.4 (s, N—C py), 165.9 (s, N—C py), 159.9 (s, C MeC_6_H_3_-py), 148.8 (s, CH py), 148.4 (s, C MeC_6_H_3_-py), 147.2 (s, CH py), 142.2 (s, C MeC_6_H_3_-py), 141.9 (s, C MeC_6_H_3_-py), 140.0 (s, C MeC_6_H_3_-py), 139.3 (s, C MeC_6_H_3_-py), 138.3 (s, CH MeC_6_H_3_-py), 138.0 (s, CH py), 137.9 (s, CH MeC_6_H_3_-py), 137.3 (s, CH py), 124.5 (s, CH MeC_6_H_3_-py), 124.4 (s, CH MeC_6_H_3_-py), 122.3 (s, CH MeC_6_H_3_-py), 122.3 (s, CH MeC_6_H_3_-py), 122.2 (s, CH py), 121.1 (s, CH py), 119.1 (s, CH py), 118.2 (s, CH py), 58.0 (s, CH_2_), 31.8 (s, C ^t^Bu), 30.6 (s, CH_3_^t^Bu), 25.8 (s, CH_3_ acetamide), 22.1 (s, CH_3_ MeC_6_H_3_-py), 21.7 (s, CH_3_ MeC_6_H_3_-py).

#### 4.1.6 Preparation of Ir{κ^1^-*N-*[NHC(CH_2_Ph)O]}{κ^2^-*C,N*-(MeC_6_H_3_-py)}_2_{=C(CH_2_^t^Bu)OH} (**7**)

To a solution of complex **4** (150 mg, 0.201 mmol) in dichloromethane (15 mL), deoxygenated water (40 μL, 2.220 mmol) was added. The solution was stirring at room temperature for 24 h and then evaporated to dryness. The crude was washed with pentane (3 × 5 mL) affording a yellow solid (72 mg, 47%). Crystals suitable for X-ray diffraction analysis were obtained by slow diffusion of pentane over a dichloromethane solution of the compound at room temperature. Anal. Calcd. for C_38_H_40_IrN_3_O_2_: C, 59.82; H, 5.28; N, 5.51. Found: C, 59.65; H, 4.97; N, 5.33. HRMS (electrospray, *m/z*): Calcd. for C_38_H_40_IrN_3_NaO_2_ [M + Na]^+^: 786.2642, found: 786.2632. IR (cm^–1^): *v*(NH) 3379 (br w), *v*(OH) 3339 (br m), *v*(CO) 1584 (s). ^1^H NMR (400 MHz, CD_2_Cl_2_, 298 K): δ 12.92 (s br, 1H, OH), 8.86 (s br, 1H, NH), 8.38 (d, ^3^*J*_H-H_*=* 5.1, 1H, CH py), 7.91 (d, ^3^*J*_H-H_*=* 8.2, 1H, CH py), 7.77 (dd, ^3^*J*_H-H_*=*^3^*J’*_H-H_ = 7.7, 1H, CH py), 7.70 (d, ^3^*J*_H-H_*=* 8.1, 1H, CH py),7.58–7.46 (m, 3H, 2H CH py + CH MeC_6_H_3_-py), 7.43 (s, 1H, CH MeC_6_H_3_-py), 7.16–7.02 (m, 4H, 3H CH Ph + CH py), 6.93–6.82 (m, 4H, 2H CH Ph + CH py + CH MeC_6_H_3_-py), 6.70 (dd, ^3^*J*_H-H_ = ^3^*J’*_H-H_ = 6.3, 1H, CH py), 6.63 (d, ^3^*J*_H-H_ = 7.8, 1H, CH MeC_6_H_3_-py), 6.37 (s, 1H, CH MeC_6_H_3_-py), 3.23 (AB system, Δν = 34.1, *J*_A–B_ = 14.7, 2 H, C*H*_2_Ph), 2.43 (s, 3H, C*H*_3_), 1.99 (s, 3H, CH_3_), 1.83 (AB system, Δν = 37.4, *J*_A–B_ = 15.0, 2 H, C*H*_2_-^t^Bu), 0.56 (s, 9H, CH_3_-^*t*^Bu). ^13^C{^1^H}-APT NMR plus HSQC and HMBC (101 MHz, CD_2_Cl_2_, 298 K): δ 229.2 (s, Ir=C), 178.8 (s, C=O), 166.3 (s, N—C py), 165.8 (s, N—C py), 159.6 (s, C MeC_6_H_3_-py), 148.5 (s, CH py), 148.3 (s, C MeC_6_H_3_-py), 147.2 (s, CH py), 142.2 (s, C MeC_6_H_3_-py), 141.8 (s, C MeC_6_H_3_-py), 140.0 (s, C MeC_6_H_3_-py), 139.4 (s, C Ph), 139.3 (s, C MeC_6_H_3_-py), 138.3 (s, CH MeC_6_H_3_-py), 137.9 (s, CH MeC_6_H_3_-py), 137.9 (s, CH py), 137.3 (s, CH py), 129.8 (s, 2C, CH Ph), 128.3 (s, 2C, CH Ph), 125.8 (s, CH Ph), 124.5 (s, CH MeC_6_H_3_-py), 124.4 (s, CH MeC_6_H_3_-py), 122.3(s, 2C CH MeC_6_H_3_-py), 122.0 (s, CH py), 121.1 (s, CH py), 119.0 (s, CH py), 118.3 (s, CH py), 57.9 (s, *C*H_2_-^t^Bu), 46.3 (s, *C*H_2_Ph), 31.8 (s, C ^t^Bu), 30.6 (s, *C*H_3_^t^Bu), 22.2 (s, CH_3_ MeC_6_H_3_-py), 21.7 (s, CH_3_ MeC_6_H_3_-py).

#### 4.1.7 Preparation of Ir{κ^1^-*N-*[NHC(CF_3_)O]}{κ^2^-*C,N*-(MeC_6_H_3_-py)}_2_{=C(CH_2_^t^Bu)OH} (**8**)

To a solution of complex **5** (150 mg, 0.208 mmol) in dichloromethane (15 mL), deoxygenated water (40 μL, 2.220 mmol) was added. The solution was stirring at room temperature for 24 h and then evaporated to dryness. The crude was washed with pentane (3 × 5 mL) affording a yellow solid (102 mg, 66%). Crystals suitable for X-ray diffraction analysis were obtained by slow diffusion of pentane over a dichloromethane solution of the compound at room temperature. Anal. Calcd. for C_32_H_33_F_3_IrN_3_O_2_: C, 51.88; H, 4.49; N, 5.67. Found: C, 51.53; H, 4.36; N, 5.75. HRMS (electrospray, *m/z*): Calcd. for C_32_H_33_F_3_IrN_3_NaO [M + Na]^+^: 764.2046, found: 764.2065. IR (cm^–1^): *v*(NH) 3385, *v*(OH) 3381 (br m), *v*(CO) 1681 (s), *v*(CF_3_) 1200, 1131. ^1^H NMR (400 MHz, CD_2_Cl_2_, 298 K): δ 11.08 (s br, 1H, OH), 8.84–8.70 (m, 2H, CH py + NH), 7.99 (d, ^3^*J*_H-H_ = 8.2, 1H, CH py), 7.88 (ddd, ^3^*J*_H-H_ = 8.2, 7.5, ^4^*J*_H-H_ = 1.6, 1H, CH py), 7.73 (d, ^3^*J*_H-H_ = 8.1, 1H, CH py), 7.62–7.48 (m, 3H, 2H CH MeC_6_H_3_-py + CH py), 7.38–7.29 (m, 2H, CH MeC_6_H_3_-py + CH py), 7.11 (ddd, ^3^*J*_H-H_ = 5.6, ^4^*J*_H-H_ = 1.5, ^5^*J*_H-H_ = 0.9, 1H, CH py), 6.93 (dd, ^3^*J*_H-H_ = 7.9, 2.3, 1H, CH MeC_6_H_3_-py), 6.75 (ddd, ^3^*J*_H-H_ = 7.1, 5.6, ^4^*J*_H-H_ = 1.5, 1H, CH py), 6.69 (dd, ^3^*J*_H-H_ = 7.9, ^4^*J*_H-H_ = 2.5, 1H, CH MeC_6_H_3_-py), 6.41 (s, 1H, CH MeC_6_H_3_-py), 2.43 (s, 3H, C*H*_3_), 2.10 (m, 2H, C*H*_2_-^t^Bu), 2.02 (s, 3H, CH_3_), 0.65 (s, 9H, C*H*_3_^t^Bu). ^13^C{^1^H}-APT NMR plus HSQC and HMBC (101 MHz, CD_2_Cl_2_, 298 K): δ 231.8 (s, Ir=C), 166.5 (s, N—C py), 165.5(s, N—C py), 162.6 (q, ^2^*J*_C-F_ = 35.2, C*—C*OCF_3_), 157.6 (s, C MeC_6_H_3_-py), 148.5 (s, CH py), 147.5 (s, CH py), 145.2 (s, C MeC_6_H_3_-py), 142.6 (s, C MeC_6_H_3_-py), 141.6 (s, C MeC_6_H_3_-py), 140.4 (s, C MeC_6_H_3_-py), 139.9 (s, C MeC_6_H_3_-py), 138.5 (s, CH py), 138.0 (s, CH MeC_6_H_3_-py), 137.7 (s, CH MeC_6_H_3_-py), 137.6 (s, CH py), 124.6 (s, CH MeC_6_H_3_-py), 124.4 (s, CH MeC_6_H_3_-py), 123.0 (s, CH MeC_6_H_3_-py), 123.0 (s, CH MeC_6_H_3_-py), 122.7 (s, CH py), 121.2 (s, CH py), 119.4 (s, CH py), 118.4 (s, CH py), 116.6 (q, ^1^*J*_C-F_ = 292.9, *C*F_3_), 58.0 (s, *C*H_2_-^t^Bu), 32.1 (s, C ^*t*^Bu), 30.7 (s, *C*H_3_-^t^Bu), 22.2 (s, CH_3_ MeC_6_H_3_-py), 21.8 (s, CH_3_ MeC_6_H_3_-py). ^19^F NMR (376.5 MHz, CD_2_Cl_2_, 298 K): δ −76.1 (s, C*F*_3_).

#### 4.1.8 Preparation of Ir{κ^4^-*N,C,C*′*,O*-[Py-MeC_6_H_3_-C(CH_2_-C_6_H_4_)NHC(*Ph*)O]}{κ^2^-*C,N*-(MeC_6_H_3_-py)} (**9**)

To a suspension of **1b** (300 mg, 0.238 mmol) in toluene (20 mL) placed in a Schlenk flask equipped with a PTFE stopcock, benzamide (58 mg, 0.476 mmol) was added. The mixture was hold during 24 h at 120 °C. The crude was cooled at room temperature, evaporated to dryness, and purified by column chromatographic (neutral Al_2_O_3_, activity grade V) using toluene as eluent to get a yellow solution. This solution was evaporated to dryness, and the addition of pentane causes the precipitation of a yellow solid, which was washed with pentane several times, giving a yellow solid (31 mg, 9%). Anal. Calcd. for C_39_H_32_IrN_3_O: C, 62.38; H, 4.30; N, 5.60. Found: C, 62.53; H, 4.00; N, 5.27. HRMS (electrospray, *m/z*): Calcd. for C_39_H_33_IrN_3_O [M + H]^+^: 752.2247, found: 752.2212. IR (cm^–1^): *v*(NH_2_) 3325 (m), *v*(CO) 1596 (s). ^1^H NMR (400 MHz, CD_2_Cl_2_, 298 K): δ 9.49 (d, ^3^*J*_H-H_ = 5.4, 1H, CH py), 8.63 (d, ^3^*J*_H-H_ = 4.8, 1H, CH py tetra), 7.99 (s, 1H, CH MeC_6_H_3_-py tetra), 7.89 (d, ^3^*J*_H-H_ = 7.9, 1H, CH py), 7.83 (ddd, ^3^*J*_H-H_ = ^3^*J’*_H-H_ = 7.9, ^4^*J*_H-H_ = 1.9, 1H, CH py), 7.56 (ddd, ^3^*J*_H-H_ = ^3^*J’*_H-H_ = 7.8, ^4^*J*_H-H_ = 1.8, 1H, CH py tetra), 7.53–7.47 (m, 3H, CH py tetra +2 CH COPh), 7.44–7.35 (m, 3H, CH py + CH COPh + CH MeC_6_H_3_-py), 7.33 (d, ^3^*J*_H-H_ = 7.8, 1H, CH MeC_6_H_3_-py tetra), 7.27 (t, ^3^*J*_H-H_ = 7.7, 2H, CH COPh), 7.21 (d, ^3^*J*_H-H_ = 7.8, 1H, CH MeC_6_H_3_-py tetra), 7.00 (s, 1H, NH), 6.98–6.92 (m, 2H, CH py tetra + CH CH_2_Ph tetra), 6.54 (t, ^3^*J*_H-H_ = 7.3, 1H, CH CH_2_Ph tetra), 6.46 (d, ^3^*J*_H-H_ = 7.8, 1H, CH MeC_6_H_3_-py), 6.42–6.35 (m, 2H, CH MeC_6_H_3_-py + CH CH_2_Ph tetra), 5.93 (d, ^3^*J*_H-H_ = 7.3, 1H, CH CH_2_Ph tetra), 4.20 (d, ^2^*J*_H-H′_ = 15.4, 1H, CH_2_), 3.29 (d, ^2^*J*_H-H′_ = 15.4, 1H, CH_2_), 2.61 (s, 3H, CH_3_ MeC_6_H_3_-py), 1.90 (s, 3H, CH_3_ MeC_6_H_3_-py). ^13^C{^1^H}-APT NMR plus HSQC and HMBC (101 MHz, CD_2_Cl_2_, 298 K): δ 173.4 (s, Ir—O=C), 167.7 (s, N—C py), 159.5 (s, N—C py tetra), 155.6 (s, Ir—C CH_2_Ph tetra), 151.2 (s, CH py tetra), 150.5 (s, Ir—C MeC_6_H_3_-py), 150.2 (s, CH py), 149.7 (s, C MeC_6_H_3_-py tetra), 145.8 (s, C CH_2_Ph tetra), 142.6 (s, C MeC_6_H_3_-py), 140.2 (s, C MeC_6_H_3_-py tetra), 139.3 (s, CH MeC_6_H_3_-py), 139.0 (s, C MeC_6_H_3_-py), 136.9 (s, C MeC_6_H_3_-py tetra), 136.7 (s, 2C, CH py + CH py tetra), 132.9 (s, CH MeC_6_H_3_-py tetra), 132.7 (s, CH CH_2_Ph tetra), 132.1 (s, CH MeC_6_H_3_-py), 131.4 (s, C COPh), 129.0 (s, 2C, CH COPh), 127.7 (s, 2C, CH COPh), 127.0 (s, CH MeC_6_H_3_-py tetra), 126.9 (s, CH MeC_6_H_3_-py tetra), 125.0 (s, CH py tetra), 124.0 (s, CH COPh), 123.6 (s, CH CH_2_Ph tetra), 122.9 (s, CH py tetra), 122.2 (s, CH py), 121.6 (s, CH CH_2_Ph tetra), 121.3 (s, CH CH_2_Ph tetra), 120.4 (s, CH py), 118.7 (s, CH py), 55.8 (s, *C*H_2_Ph), 54.4 (s, C Ir—C—NH), 21.9 (s, CH_3_ MeC_6_H_3_-py tetra), 21.5 (s, CH_3_ MeC_6_H_3_-py).

#### 4.1.9 Preparation of Ir{κ^4^-*N,C,C*′*,O*-[Py-MeC_6_H_3_-C(CH_2_-C_6_H_4_)NHC(CH_3_)O]}{κ^2^-*C,N*-(MeC_6_H_3_-py)} (**10**)

To a suspension of **1b** (300 mg, 0.238 mmol) in toluene (20 mL) placed in a Schlenk flask equipped with a PTFE stopcock, acetamide (28 mg, 0.476 mmol) was added. The mixture was hold during 24 h at 120 °C. The crude was cooled at room temperature, evaporated to dryness, and purified by column chromatographic (neutral Al_2_O_3_, activity grade V) using toluene as eluent to get a yellow solution. This solution was evaporated to dryness, and the addition of pentane causes the precipitation of a yellow solid, which was washed with pentane several times, giving a yellow solid (47 mg, 14%). Anal. Calcd. for C_34_H_30_IrN_3_O: C, 59.28; H, 4.39; N, 6.10. Found: C, 59.61; H, 4.16; N, 6.45. HRMS (electrospray, *m/z*): Calcd. for C_34_H_30_IrN_3_O [M]^+^: 689.2013, found: 689.2010. IR (cm^–1^): *v*(NH_2_) 3335 (m), *v*(CO) 1586 (s). ^1^H NMR (400 MHz, CD_2_Cl_2_, 298 K): δ 9.32 (d, ^3^*J*_H-H_ = 5.4, 1H, CH py), 8.54 (d, ^3^*J*_H-H_ = 5.5, 1H, CH py), 7.92–7.83 (m, 2H, CH MeC_6_H_3_-py + CH MeC_6_H_3_-py), 7.80 (ddd, ^3^*J*_H-H_ = ^3^*J’*_H-H_ = 7.7, ^4^*J*_H-H_ = 1.8, 1H, CH py), 7.65 (ddd, ^3^*J*_H-H_ = ^3^*J’*_H-H_ = 7.8, ^4^*J*_H-H_ = 1.8, 1H, CH py), 7.57 (d, ^3^*J*_H-H_ = 7.8, 1H, CH py), 7.41–7.29 (m, 3H, CH py + CH MeC_6_H_3_-py + CH MeC_6_H_3_-py), 7.19 (d, ^3^*J*_H-H_ = 7.8, 1H, CH MeC_6_H_3_-py), 7.00–6.89 (m, 2H, CH py + CH Ph), 6.55 (ddd, ^3^*J*_H-H_ = ^3^*J’*_H-H_ = 7.3, ^4^*J*_H-H_ = 1.4, 1H, CH Ph), 6.43 (dd, ^3^*J*_H-H_ = 7.7, ^4^*J*_H-H_ = 1.8, 1H, CH py), 6.41–6.29 (m, 3H, CH MeC_6_H_3_-py + CH Ph + NH), 5.87 (d, ^3^*J*_H-H_ = 7.3, 1H, CH Ph), 4.08 (d, ^2^*J*_H-H′_ = 15.3, 1H, CH_2_), 3.13 (d, ^2^*J*_H-H′_ = 15.3, 1H, CH_2_), 2.58 (s, 3H, CH_3_ MeC_6_H_3_-py), 1.87 (s, 3H, CH_3_ MeC_6_H_3_-py), 1.67 (s, 3H, COC*H*_3_). ^13^C{^1^H}-APT NMR plus HSQC and HMBC (101 MHz, CD_2_Cl_2_, 253 K): δ 176.0 (s, Ir—O=C), 167.6 (s, N—C py), 159.5 (s, N—C py), 155.8 (s, C Ph), 151.2 (s, CH py), 150.3 (s, C MeC_6_H_3_-py), 150.2 (s, CH py), 149.8 (s, C MeC_6_H_3_-py), 145.9 (s, C Ph), 142.5 (s, C MeC_6_H_3_-py), 140.2 (s, C MeC_6_H_3_-py), 139.3 (s, CH MeC_6_H_3_-py), 139.0 (s, C MeC_6_H_3_-py), 136.8 (s, C MeC_6_H_3_-py), 136.6 (s, CH py), 136.6 (s, CH py), 132.9 (s, CH MeC_6_H_3_-py), 132.7 (s, CH Ph), 126.9 (s, CH MeC_6_H_3_-py), 126.7 (s, CH MeC_6_H_3_-py), 125.0 (s, CH py), 124.0 (s, CH Ph), 123.6 (s, CH Ph), 123.0 (s, CH py), 122.2 (s, CH py), 121.6 (s, CH Ph), 121.3 (s, CH Ph), 120.3 (s, CH py), 118.6 (s, CH MeC_6_H_3_-py), 55.5 (s, *C*H_2_Ph), 53.9 (s, C Ir—C—NH), 21.8 (s, CH_3_ MeC_6_H_3_-py tetra), 21.5 (s, CH_3_ MeC_6_H_3_-py). 19.7 (s, CO*C*H_3_).

## Supporting Information Available

General information for the experimental section, structural analysis of complexes 2, 7, 8, and 10, computational details, energies of optimized structures, NICS scan curve for complex 2, induced current density (ACID) of complex 2, NBO7 analysis and π NBO orbitals for complexes OsH{κ^2^-*C*,*O*-[C(Ph)NC(CH_3_)O]}(IPr)(P^i^Pr_3_) and 3, observed and calculated UV-vis spectra of complexes 2-4 and 6-10, analysis of computed UV/Vis data, theoretical analysis of molecular orbitals, spin density distribution for the optimized triplet T^_1_^, cyclic voltammograms of complexes 2 and 6-10, normalized excitation and emission spectra of complexes 2-4 and 6-10, and NMR spectra. (PDF)
Atomic coordinates of complexes **2**–**10** (XYZ)

## Supplementary Material

ic2c03522_si_001.pdf

ic2c03522_si_002.xyz
