# Supplementary material for: Acetylides for the Preparation of Phosphorescent Iridium(III) Complexes: Iridaoxazoles and Their Transformation into Hydroxycarbenes and N,C(sp3),C(sp2),O-Tetradentate Ligands
Source: Inorg Chem. 2022 Nov 23;61(48):19597–611. doi: 10.1021/acs.inorgchem.2c03522 (PMC9949702; doi:10.1021/acs.inorgchem.2c03522)
Supplement: Supplementary file 1 — ic2c03522_si_001.pdf [file ic2c03522_si_001.pdf]

Supporting Information For

Acetylides for the Preparation of Phosphorescent Iridium(III) Complexes: Iridaoxazoles and their Transformation into Hydroxycarbenes and *N,C(sp^3),C(sp^2),O* – Tetradentate Ligands

María Benítez,[1] María L. Buil,[1] Miguel A. Esteruelas,[,1] Susana Izquierdo,[1] Enrique Oñate,[1] and Jui-Yi Tsai[2]*

[1]Departamento de Química Inorgánica, Instituto de Síntesis Química y Catálisis Homogénea (ISQCH), Centro de Innovación en Química Avanzada (ORFEO-CINQA), Universidad de Zaragoza – CSIC, 50009 Zaragoza, Spain

[2]Universal Display Corporation, Ewing, New Jersey 08618, United States

*Corresponding author's e-mail address: maester@unizar.es

CONTENTS

Experimental section: Instrumental methods S3

Structural Analysis of Complexes 2, 7, 8, and 10 S4

Computational Details S6

Energies of Optimized Structures of 2-10 S6

NICS scan curve for complex 2 S11

Induced current density (ACID) of complex 2 S11

NBO7 analysis and π NBO orbitals for complexes OsH{κ^2-*C,O*-[C(Ph)NC(CH_3)O]}(IPr)(P^iPr_3) and 3 S12

UV-vis Spectra of Complexes 2-4 and 6-10 (Observed and Calculated) S13

Analysis of Computed UV/Vis Data of Complexes 2-4 and 6-10 S16

Theoretical Analysis of Molecular Orbitals of Complexes 2-10 S24

Spin Density Distributions for the Optimized Triplet T_1 of 2-10 S42

Cyclic Voltammograms of Complexes 2 and 6-10 S43

Normalized Excitation and Emission Spectra of Complexes 2-4 and 6-10 S44

NMR spectra S52

References S64

Experimental section: Instrumental methods. Solvents were dried by the usual procedures and distilled under argon atmosphere or from an MBraun solvent purification apparatus. NMR spectra were recorded on a Bruker ARX 300, Bruker Avance 300 MHz or Bruker Avance 400 MHz instruments. Elemental analyses were carried out in a Perkin-Elmer 2400-B Series II CHNS-Analyzer. High-resolution electrospray (HRMS) mass spectra were acquired using a MicroTOF-Q hybrid quadrupole time-of-flight spectrometer (Bruker Daltonics, Bremen, Germany). Attenuated total reflection infrared spectra (ATR-IR) of solid samples were run on a Perkin-Elmer Spectrum 100 FT-IR spectrometer. UV-visible spectra were registered on an Evolution 600 spectrophotomer. Steady-state photoluminescence spectra were recorded on a Jobin-Yvon Horiba Fluorolog FL-3-11 spectrofluorometer. Lifetimes were measured using an IBH 5000F coaxial nanosecond flash lamp. Quantum yields were measured using the Hamamatsu Absolute PL Quantum Yield Measurement System C11347-11. Cyclic voltammetry measurements were performed using a Voltalab PST050 potentiostat with Pt wire as working electrode, Pt wire as counter electrode, and saturated calomel (SCE) as reference electrode. The experiments were carried out under argon in dicloromethane solutions (10^{-3} M), with Bu_4NPF_6 as supporting electrolyte (0.1 M). Scan rate was 100 mV·s-1. The potentials were referenced to the ferrocene/ferrocenium (Fc/Fc^+) couple.

Structural Analysis of Complexes 2, 7, 8, and 10. X-ray data were collected on a APEX D8 Venture Bruker diffractometer (Mo radiation, λ = 0.71073 Å). The crystals were cooled with a nitrogen flow with a Oxford Cryosystems systems. Data were corrected for absorption by using a multiscan method applied with the SADABS program.[1] The structures were solved by Patterson or direct methods and refined by full-matrix least squares on F^2 with SHELXL2019,[2] including isotropic and subsequently anisotropic displacement parameters. The hydrogen atoms were observed in the last Fourier Maps or calculated, and refined freely or using a restricted riding model.

For **7** the OH groups were observed disordered at 50% in two positions corresponding to an intramolecular or intermolecular hydrogen bonds.

Crystal data for **2**: $C_{37}H_{36}IrN_3O$, M_W 730.89, red, irregular block (0.270 x 0.070 x 0.040 mm^3), monoclinic, space group P21/n, *a*: 11.1935(5) Å, *b*: 19.7924(9) Å, *c*: 13.9164(6) Å, *β*: 92.797(2)°, *V* = 3079.5(2) Å^3, *Z* = 4, *Z'* = 1, D_{calc}: 1.576 g cm^{-3}, F(000): 1456, T = 100(2) K, μ 4.370 mm^{-1}. 115817 measured reflections (2θ: 3-57°, ω and φ scans 0.5°), 7658 unique (R_{int} = 0.0307); min./max. transm. Factors 0.637/0.862. Final agreement factors were R^1 = 0.0186 (7207 observed reflections, I > 2σ(I)) and wR^2 = 0.0467; data/restraints/parameters 7658/0/384; GoF = 0.995. Largest peak and hole 1.850 (close to Ir atoms) and -0.464 e/ Å^3.

Crystal data for **7**: $C_{38}H_{40}IrN_3O_2$, 0.5(C_5H_{12}), 0.25(H_2O), M_W 803.50, yellow, irregular block (0.250 x 0.250 x 0.080 mm^3), triclinic, space group P-1, *a*: 10.0550(6) Å, *b*: 12.6906(8) Å, *c*: 16.2052(10) Å, *α*: 76.380(3)°, *β*: 84.497(3)°, *γ*: 74.244(2)°, *V* = 1933.1(2) Å^3, *Z* = 2, *Z'* = 1, D_{calc}: 1.380 g cm^{-3}, F(000): 811, T = 100(2) K, μ 3.489 mm^{-1}. 41830 measured reflections (2θ: 3-57°, ω and φ scans 0.5°), 11591 unique (R_{int} = 0.0291); min./max. transm. Factors 0.721/0.862. Final agreement factors were R^1 =

0.0427 (10768 observed reflections, I > 2σ(I)) and wR^2 = 0.1103; data/restraints/parameters 11591/7/438; GoF = 1.288. Largest peak and hole 2.574 (close to Ir atoms or solvent disordered molecules) and -3.485 e/ Å^3.

Crystal data for **8**: $C_{32}H_{33}F_3IrN_3O_2$, M_W 740.81, green, irregular block (0.130 x 0.112 x 0.110 mm^3), monoclinic, space group P21, *a*: 10.8176(5) Å, *b*: 23.1311(11) Å, *c*: 12.7553(6)Å, *β*: 112.980(2)°, *V* = 2938.4(2) Å^3, *Z* = 4, *Z'* = 2, D_{calc}: 1.675 g cm^{-3}, F(000): 1464, T = 100(2) K, μ 4.597 mm^{-1}. 77473 measured reflections (2θ: 3-57°, ω and φ scans 0.5°), 14553 unique (R_{int} = 0.0410); min./max. transm. Factors 0.586/0.862. Final agreement factors were R^1 = 0.0294 (14262 observed reflections, I > 2σ(I)) and wR^2 = 0.0713; flack parameter 0.4440(8); data/restraints/parameters 14553/22/751; GoF = 1.481. Largest peak and hole 2.972 (close to Ir atoms) and -1.186 e/ Å^3.

Crystal data for **10**: $C_{34}H_{30}IrN_3O$, 0.783(CH_2Cl_2), M_W 755.33, yellow, irregular block (0.187 x 0.041 x 0.032 mm^3), trigonal, space group R -3, *a*: 35.5661(7) Å, *b*: 35.5661(7)Å, *c*: 13.7458(4) Å, *V* = 15058.2(7) Å^3, *Z* = 18, *Z'* = 1, D_{calc}: 1.499 g cm^{-3}, F(000): 6712, T = 100(2) K, μ 4.145 mm^{-1}. 232663 measured reflections (2θ: 3-57°, ω and φ scans 0.5°), 13848 unique (R_{int} = 0.0542); min./max. transm. Factors 0.688/0.862. Final agreement factors were R^1 = 0.0356 (13234 observed reflections, I > 2σ(I)) and wR^2 = 0.0880; data/restraints/parameters 13848/12/388; GoF = 1.133. Largest peak and hole 2.116 (close to Ir atoms) and -2.414 e/ Å^3.

Computational Details. All calculations were performed at the DFT level using the B3LYP functional[3] supplemented with the Grimme's dispersion correction D3[4] as implemented in Gaussian09.[5] Ir atoms were described by means of an effective core potential SDD for the inner electron[6] and its associated double-ζ basis set for the outer ones, complemented with a set of f-polarization functions for iridium.[7] The 6-31G** basis set was used for the H, C, N, and O atoms.[8] All minima were verified to have no negative frequencies. The geometries were fully optimized in THF (ε = 7.4257) or Toluene (ε = 2.3741) solvents using the continuum SMD model.[9] We performed TD-DFT calculations at the same level of theory in THF calculating the lowest 50 singlet-singlet excitations at the ground state S_0. It should be noted that the singlet-triplet excitations are set to zero due to the neglect of spin-orbit coupling in the TDDFT calculations as implemented in G09. The UV/vis absorption spectra were obtained by using the GaussSum 3 software.[10] The phosphorescence emission compares well with the 0-0 transition calculated taking into account the zero point energies (zpe) of the geometries of both the optimized T_1 and S_0 states in THF.

Energies of Optimized Structures of 2-10

Complex 2 S_0 (THF)

Zero-point correction= 0.639579 (Hartree/Particle)
Thermal correction to Energy= 0.677853
Thermal correction to Enthalpy= 0.678797
Thermal correction to Gibbs Free Energy= 0.567816
Sum of electronic and zero-point Energies= -1775.193036
Sum of electronic and thermal Energies= -1775.154762
Sum of electronic and thermal Enthalpies= -1775.153818
Sum of electronic and thermal Free Energies= -1775.264799

Complex 2 T_1(THF)

Zero-point correction= 0.636419 (Hartree/Particle)
Thermal correction to Energy= 0.674484
Thermal correction to Enthalpy= 0.675428
Thermal correction to Gibbs Free Energy= 0.564192

Sum of electronic and zero-point Energies= -1775.120395
Sum of electronic and thermal Energies= -1775.082330
Sum of electronic and thermal Enthalpies= -1775.081385
Sum of electronic and thermal Free Energies= -1775.192621

Complex 3 S_0 (Toluene)

Zero-point correction= 0.585537 (Hartree/Particle)
Thermal correction to Energy= 0.621418
Thermal correction to Enthalpy= 0.622362
Thermal correction to Gibbs Free Energy= 0.515428
Sum of electronic and zero-point Energies= -1583.488388
Sum of electronic and thermal Energies= -1583.452508
Sum of electronic and thermal Enthalpies= -1583.451564
Sum of electronic and thermal Free Energies= -1583.558498

Complex 3 T_1 (Toluene)

Zero-point correction= 0.582895 (Hartree/Particle)
Thermal correction to Energy= 0.619148
Thermal correction to Enthalpy= 0.620092
Thermal correction to Gibbs Free Energy= 0.512396
Sum of electronic and zero-point Energies= -1583.403334
Sum of electronic and thermal Energies= -1583.367080
Sum of electronic and thermal Enthalpies= -1583.366136
Sum of electronic and thermal Free Energies= -1583.473832

Complex 4 S_0 (Toluene)

Zero-point correction= 0.668102 (Hartree/Particle)
Thermal correction to Energy= 0.707981
Thermal correction to Enthalpy= 0.708925
Thermal correction to Gibbs Free Energy= 0.592809
Sum of electronic and zero-point Energies= -1814.482958
Sum of electronic and thermal Energies= -1814.443080
Sum of electronic and thermal Enthalpies= -1814.442135
Sum of electronic and thermal Free Energies= -1814.558252

Complex 4 T_1 (Toluene)

Zero-point correction= 0.665058 (Hartree/Particle)
Thermal correction to Energy= 0.705526
Thermal correction to Enthalpy= 0.706470
Thermal correction to Gibbs Free Energy= 0.587984
Sum of electronic and zero-point Energies= -1814.400626
Sum of electronic and thermal Energies= -1814.360158
Sum of electronic and thermal Enthalpies= -1814.359214
Sum of electronic and thermal Free Energies= -1814.477700

Complex 5 S_0 (THF)

Zero-point correction= 0.562489 (Hartree/Particle)
Thermal correction to Energy= 0.600103
Thermal correction to Enthalpy= 0.601048
Thermal correction to Gibbs Free Energy= 0.489953
Sum of electronic and zero-point Energies= -1881.217584
Sum of electronic and thermal Energies= -1881.179970
Sum of electronic and thermal Enthalpies= -1881.179025
Sum of electronic and thermal Free Energies= -1881.290120

Complex 5 T_1 (THF)

Zero-point correction= 0.560337 (Hartree/Particle)
Thermal correction to Energy= 0.598181
Thermal correction to Enthalpy= 0.599125
Thermal correction to Gibbs Free Energy= 0.487810
Sum of electronic and zero-point Energies= -1881.147809
Sum of electronic and thermal Energies= -1881.109965
Sum of electronic and thermal Enthalpies= -1881.109021
Sum of electronic and thermal Free Energies= -1881.220336

Complex 6 S_0 (THF)

Zero-point correction= 0.612095 (Hartree/Particle)
Thermal correction to Energy= 0.649436
Thermal correction to Enthalpy= 0.650380
Thermal correction to Gibbs Free Energy= 0.542179
Sum of electronic and zero-point Energies= -1659.925650
Sum of electronic and thermal Energies= -1659.888308
Sum of electronic and thermal Enthalpies= -1659.887364
Sum of electronic and thermal Free Energies= -1659.995565

Complex 6 T_1 (THF)

Zero-point correction= 0.608333 (Hartree/Particle)
Thermal correction to Energy= 0.646247
Thermal correction to Enthalpy= 0.647191
Thermal correction to Gibbs Free Energy= 0.537182
Sum of electronic and zero-point Energies= -1659.834584
Sum of electronic and thermal Energies= -1659.796670
Sum of electronic and thermal Enthalpies= -1659.795726
Sum of electronic and thermal Free Energies= -1659.905734

Complex 7 S_0 (THF)

Zero-point correction= 0.694163 (Hartree/Particle)
Thermal correction to Energy= 0.735538
Thermal correction to Enthalpy= 0.736482
Thermal correction to Gibbs Free Energy= 0.618458

Sum of electronic and zero-point Energies= -1890.922165
Sum of electronic and thermal Energies= -1890.880790
Sum of electronic and thermal Enthalpies= -1890.879846
Sum of electronic and thermal Free Energies= -1890.997870

Complex 7 T_1 (THF)

Zero-point correction= 0.690266 (Hartree/Particle)
Thermal correction to Energy= 0.732378
Thermal correction to Enthalpy= 0.733322
Thermal correction to Gibbs Free Energy= 0.612654
Sum of electronic and zero-point Energies= -1890.831674
Sum of electronic and thermal Energies= -1890.789562
Sum of electronic and thermal Enthalpies= -1890.788618
Sum of electronic and thermal Free Energies= -1890.909286

Complex 8 S_0 (THF)

Zero-point correction= 0.590189 (Hartree/Particle)
Thermal correction to Energy= 0.629253
Thermal correction to Enthalpy= 0.630197
Thermal correction to Gibbs Free Energy= 0.517145
Sum of electronic and zero-point Energies= -1957.654762
Sum of electronic and thermal Energies= -1957.615698
Sum of electronic and thermal Enthalpies= -1957.614754
Sum of electronic and thermal Free Energies= -1957.727806

Complex 8 T_1 (THF)

Zero-point correction= 0.586940 (Hartree/Particle)
Thermal correction to Energy= 0.626479
Thermal correction to Enthalpy= 0.627423
Thermal correction to Gibbs Free Energy= 0.512742
Sum of electronic and zero-point Energies= -1957.551093
Sum of electronic and thermal Energies= -1957.511555
Sum of electronic and thermal Enthalpies= -1957.510611
Sum of electronic and thermal Free Energies= -1957.625292

Complex 9 S_0 (THF)

Zero-point correction= 0.610251 (Hartree/Particle)
Thermal correction to Energy= 0.646944
Thermal correction to Enthalpy= 0.647888
Thermal correction to Gibbs Free Energy= 0.540752
Sum of electronic and zero-point Energies= -1849.014931
Sum of electronic and thermal Energies= -1848.978238
Sum of electronic and thermal Enthalpies= -1848.977294
Sum of electronic and thermal Free Energies= -1849.084430

Complex 9 T_1 (THF)

Zero-point correction= 0.606321 (Hartree/Particle)
Thermal correction to Energy= 0.643664
Thermal correction to Enthalpy= 0.644608
Thermal correction to Gibbs Free Energy= 0.534985
Sum of electronic and zero-point Energies= -1848.930817
Sum of electronic and thermal Energies= -1848.893474
Sum of electronic and thermal Enthalpies= -1848.892530
Sum of electronic and thermal Free Energies= -1849.002153

Complex 10 S_0 (THF)

Zero-point correction= 0.556801 (Hartree/Particle)
Thermal correction to Energy= 0.590542
Thermal correction to Enthalpy= 0.591486
Thermal correction to Gibbs Free Energy= 0.491269
Sum of electronic and zero-point Energies= -1657.315027
Sum of electronic and thermal Energies= -1657.281286
Sum of electronic and thermal Enthalpies= -1657.280342
Sum of electronic and thermal Free Energies= -1657.380558

Complex 10 T_1 (THF)

Zero-point correction= 0.553401 (Hartree/Particle)
Thermal correction to Energy= 0.587563
Thermal correction to Enthalpy= 0.588508
Thermal correction to Gibbs Free Energy= 0.487392
Sum of electronic and zero-point Energies= -1657.230532
Sum of electronic and thermal Energies= -1657.196370
Sum of electronic and thermal Enthalpies= -1657.195426
Sum of electronic and thermal Free Energies= -1657.296541

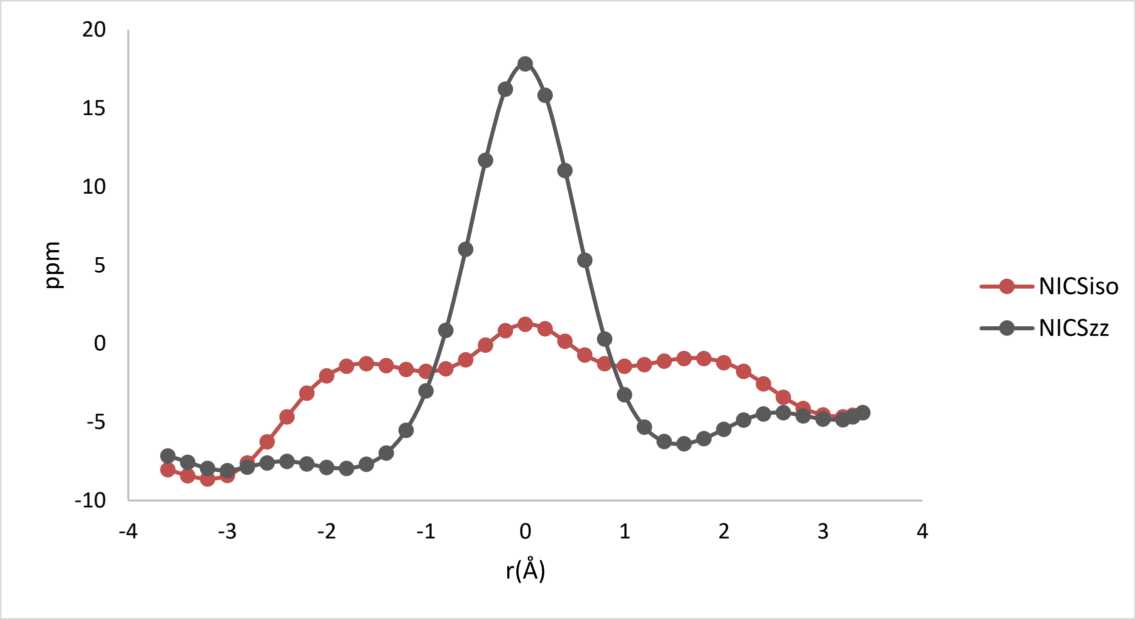

Figure S1. NICS scan curve for complex **2**.

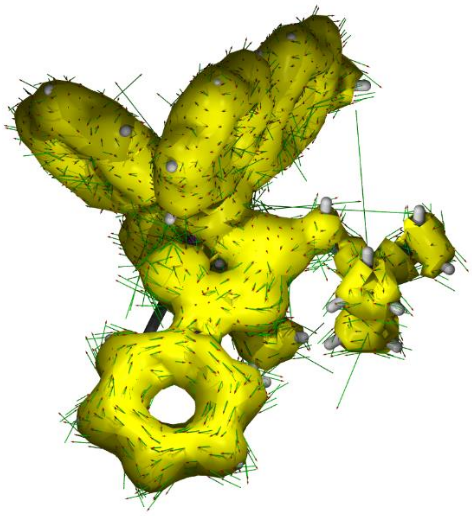

Figure S2. AICD plot of complex **2** with an isosurface value of 0.03.

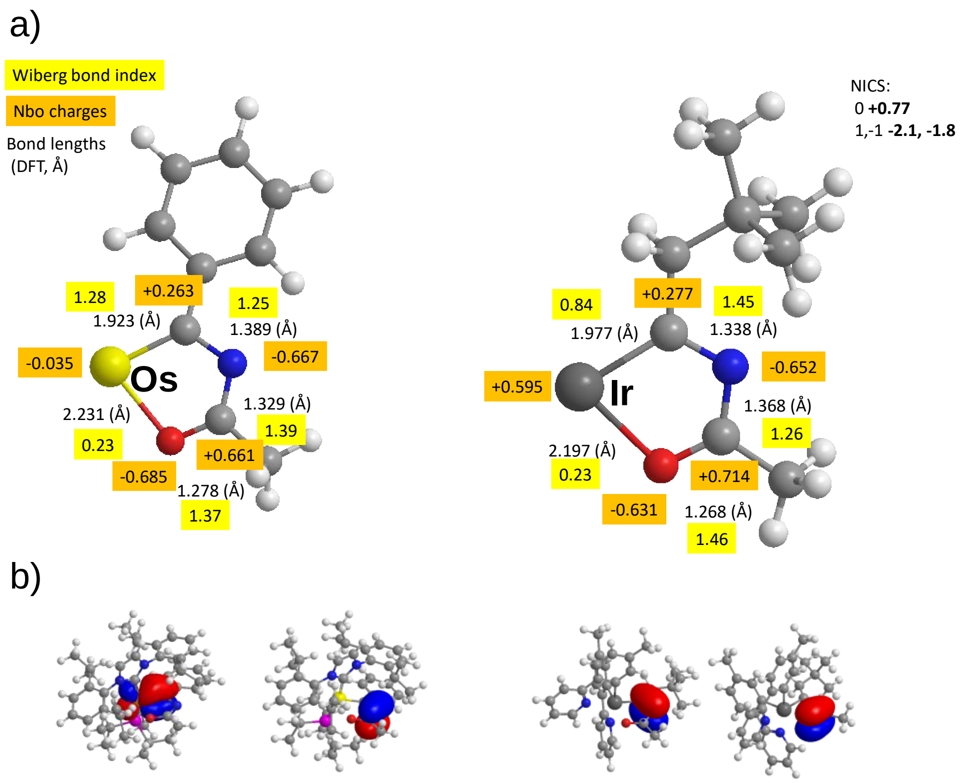

Figure S3. NBO7 analysis (a) and π NBO orbitals (b. Isovalue 0.03) for the matalaoxazole ring of complexes OsH{κ^2-*C*,*O*-[C(Ph)NC(CH_3)O]}(IPr)(P^iPr_3) and **3**.[11]

UV-vis Spectra of Complexes 2-4 and 6-10 (Observed and Calculated)

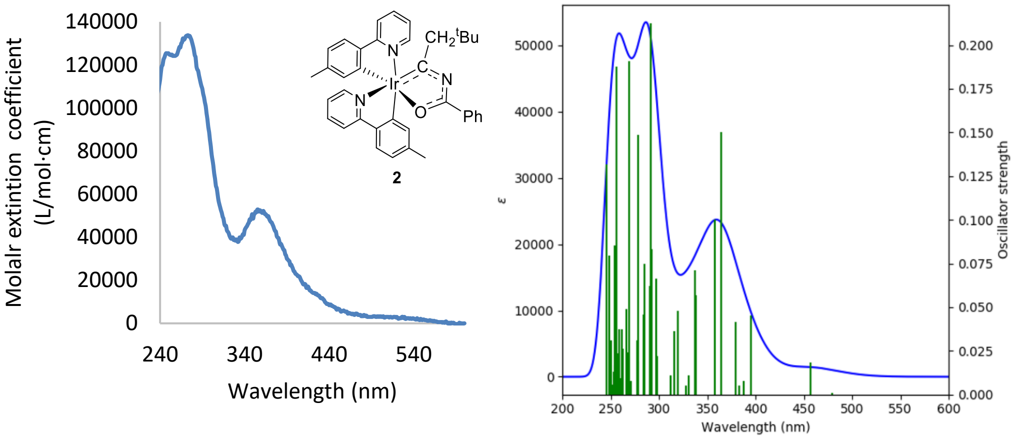

Figure S4. Observed UV-vis spectrum of complex **2** in 2-MeTHF (1.0 x 10^{-5} M) and calculated (B3LYP-D3//SDD(f)/6-31G**) in THF.

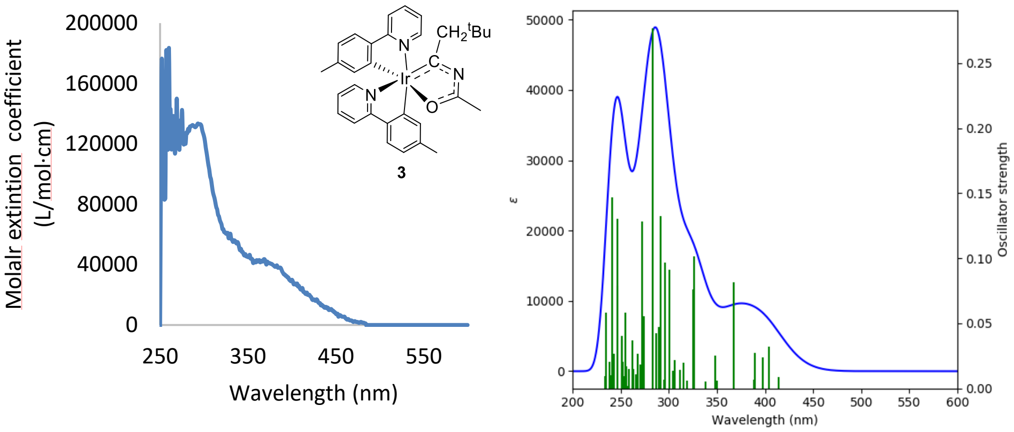

Figure S5. Observed UV-vis spectrum of complex **3** in toluene (1.0 x 10^{-5} M) and calculated (B3LYP-D3//SDD(f)/6-31G**) in toluene.

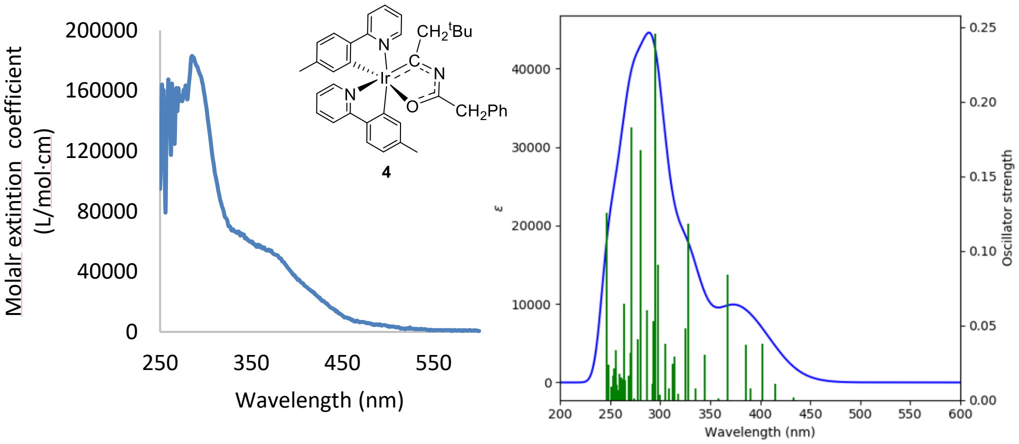

Figure S6. Observed UV-vis spectrum of complex **4** in toluene (1.0 x 10^{-5} M) and calculated (B3LYP-D3//SDD(f)/6-31G**) in toluene.

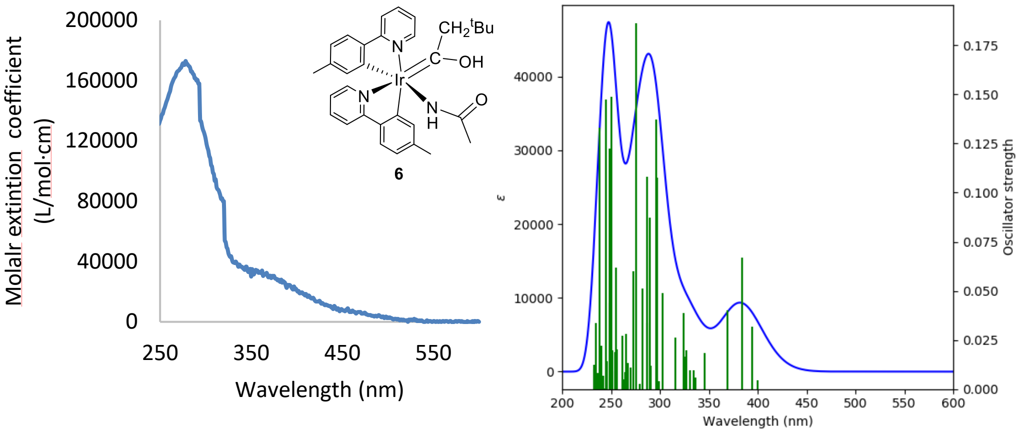

Figure S7. Observed UV-vis spectrum of complex **6** in 2-MeTHF (1.0 x 10^{-5} M) and calculated (B3LYP-D3//SDD(f)/6-31G**) in THF.

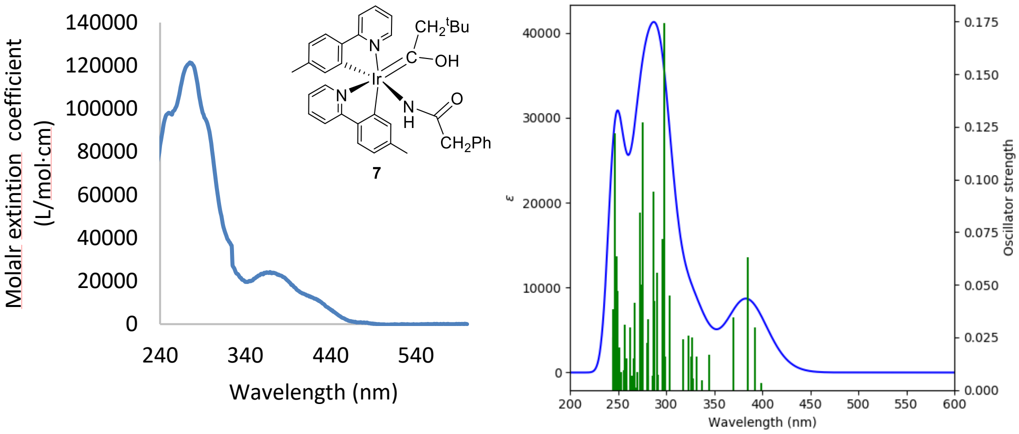

Figure S8. Observed UV-vis spectrum of complex **7** in 2-MeTHF (1.0 x 10^{-5} M) and calculated (B3LYP-D3//SDD(f)/6-31G**) in THF.

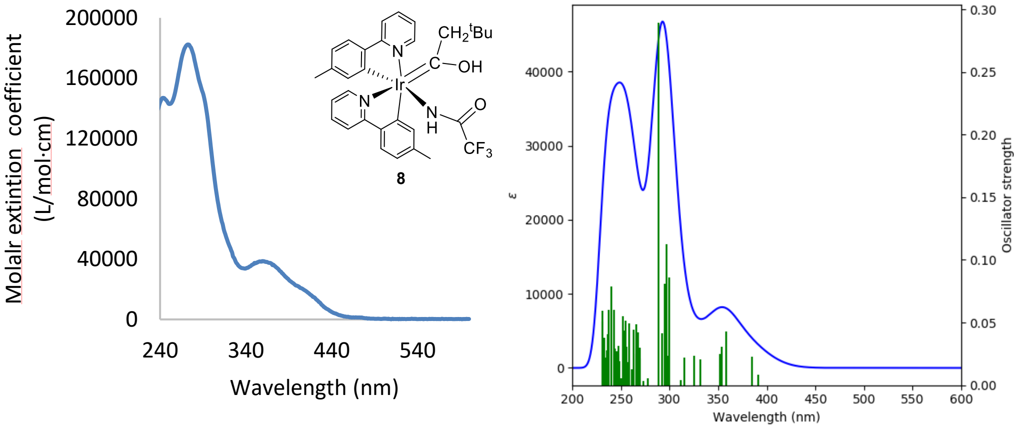

Figure S9. Observed UV-vis spectrum of complex **8** in 2-MeTHF (1.0 x 10^{-5} M) and calculated (B3LYP-D3//SDD(f)/6-31G**) in THF.

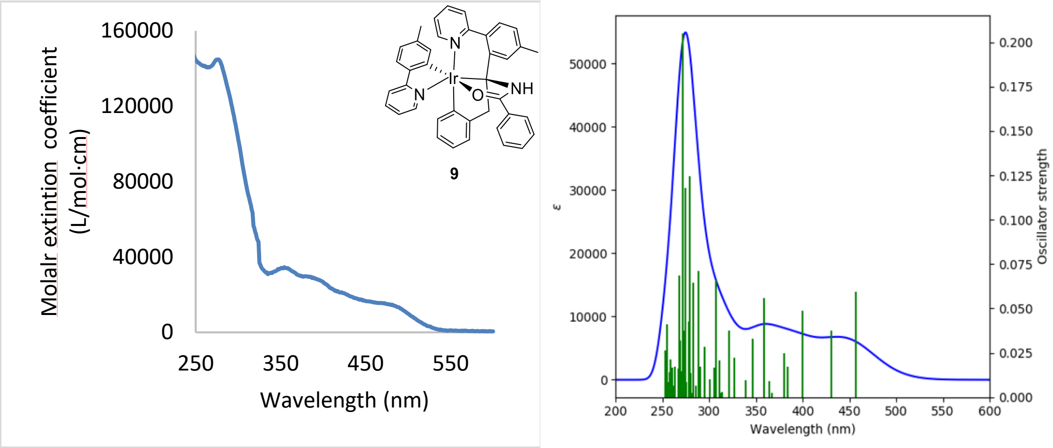

Figure S10. Observed UV-vis spectrum of complex **9** in 2-MeTHF (1.0 x 10^{-5} M) and calculated (B3LYP-D3//SDD(f)/6-31G**) in THF.

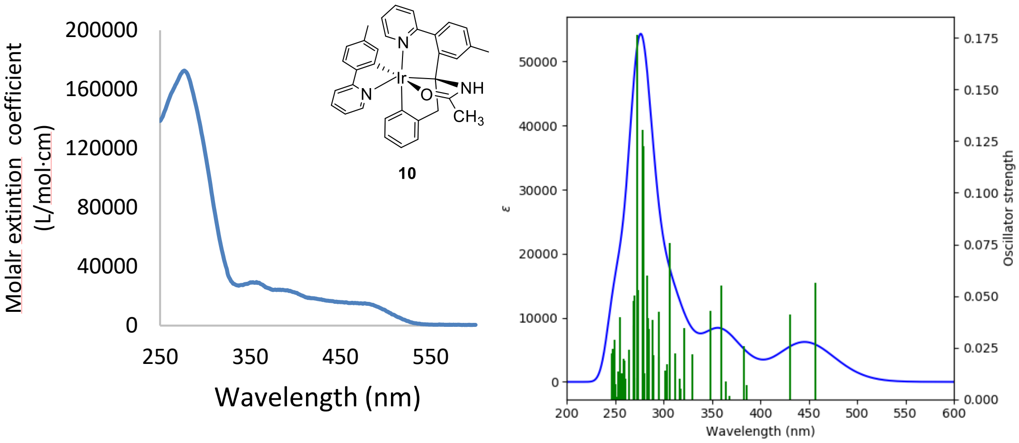

Figure S11. Observed UV-vis spectrum of complex **10** in 2-MeTHF (1.0 x 10^{-5} M) and calculated (B3LYP-D3//SDD(f)/6-31G**) in THF.

Analysis of Computed UV/Vis Data of Complexes 2-4 and 6-10

Table S1. Selected transitions for the calculated UV spectrum of complex 2 in THF

λ (nm)	Osc. Strength	Symmetry	Major contributions	Minor contributions
500	0	Triplet-A	HOMO->LUMO (85%)	H-3->LUMO (2%), H-1->LUMO (6%)
457	0.0181	Singlet-A	H-1->LUMO (93%)	H-2->LUMO (4%)
395	0.0454	Singlet-A	HOMO->L+1 (95%)	-
364	0.1503	Singlet-A	H-2->LUMO (12%), H-1->L+2 (65%)	H-5->LUMO (2%), H-4->LUMO (8%), HOMO->L+2 (8%)
358	0.0999	Singlet-A	H-4->LUMO (11%), H-2->LUMO (57%), H-1->L+2 (17%)	H-5->LUMO (4%), H-1->LUMO (3%), HOMO->L+2 (3%)
292	0.2126	Singlet-A	H-5->L+2 (11%), H-3->L+2 (62%)	H-5->L+1 (6%), H-4->L+1 (2%), H-3->L+1 (4%), H-2->L+2 (3%)
278	0.1484	Singlet-A	H-8->LUMO (15%), H-2->L+3 (47%)	H-9->LUMO (3%), H-7->LUMO (4%), H-5->L+2 (3%), H-4->L+1 (5%), H-4->L+2 (3%), H-4->L+3 (5%), H-3->L+3 (2%), H-2->L+4 (3%)
269	0.1910	Singlet-A	H-2->L+4 (70%)	H-4->L+1 (3%), H-4->L+4 (5%), H-3->L+3 (6%), H-3->L+4 (2%)
250	0.0311	Singlet-A	H-5->L+4 (81%)	H-7->L+1 (2%), H-1->L+8 (4%), HOMO->L+8 (2%)

Table S2. Composition (%) of the selected transitions for the calculated UV-vis spectrum of complex 2

λ (nm)	Osc. Strength	Symmetry	Ir	Ph-Py (1)	Ph-Py(2)	Metallacycle
500	0	Triplet-A	42-->7 (-35)	16-->1 (-15)	36-->2 (-34)	6-->90 (84)
457	0.0181	Singlet-A	40-->7 (-33)	43-->1 (-42)	12-->2 (-10)	5-->90 (85)
395	0.0454	Singlet-A	43-->3 (-40)	12-->15 (3)	38-->80 (42)	6-->1 (-5)
364	0.1503	Singlet-A	38-->5 (-33)	34-->62 (28)	19-->11 (-8)	8-->22 (14)
358	0.0999	Singlet-A	32-->7 (-25)	25-->18 (-7)	30-->5 (-25)	13-->71 (58)
292	0.2126	Singlet-A	14-->5 (-9)	52-->71 (19)	14-->24 (10)	21-->1 (-20)
278	0.1484	Singlet-A	23-->4 (-19)	19-->24 (5)	29-->50 (21)	29-->22 (-7)
269	0.1910	Singlet-A	27-->2 (-25)	27-->66 (39)	32-->31 (-1)	13-->0 (-13)
250	0.0311	Singlet-A	17-->3 (-14)	12-->68 (56)	23-->29 (6)	48-->1 (-47)

Table S3. Selected transitions for the calculated UV spectrum of complex 3 in Toluene

λ (nm)	Osc. Strength	Symmetry	Major contributions	Minor contributions
464	0	Triplet-A	HOMO->LUMO (64%)	H-5->LUMO (2%), H-2->LUMO (7%), H-1->LUMO (7%), HOMO->L+1 (6%)
405	0.0320	Singlet-A	HOMO->LUMO (55%), HOMO->L+2 (29%)	H-1->LUMO (2%), HOMO->L+1 (8%), HOMO->L+3 (2%)
367	0.0818	Singlet-A	H-1->L+1 (61%), H-1->L+2 (20%)	HOMO->L+1 (9%), HOMO->L+2 (3%), HOMO->L+3 (2%)
348	0.0255	Singlet-A	HOMO->L+3 (83%)	-
326	0.1017	Singlet-A	H-2->LUMO (57%), HOMO->L+4 (25%)	H-4->LUMO (8%), H-4->L+2 (2%)
300	0.0910	Singlet-A	H-5->LUMO (61%)	H-4->LUMO (2%), H-3->LUMO (5%), H-3->L+1 (7%), H-3->L+2 (9%), H-2->L+3 (9%)
296	0.0970	Singlet-A	H-3->L+1 (64%)	H-5->LUMO (4%), H-5->L+1 (3%), H-5->L+2 (2%), H-4->LUMO (4%), H-4->L+1 (2%), H-4->L+2 (7%), H-3->L+2 (5%)
262	0.0371	Singlet-A	H-3->L+4 (55%), H-1->L+5 (17%)	H-6->LUMO (5%), H-6->L+1 (7%) H-2->L+4 (3%)

Table S4. Composition (%) of the selected transitions for the calculated UV-vis spectrum of complex 3

λ (nm)	Osc. Strength	Symmetry	Ir	Ph-Py (1)	Ph-Py(2)	Metallacycle
464	0	Triplet-A	41-->3 (-38)	17-->13 (-4)	35-->79 (44)	7-->6 (-1)
405	0.0320	Singlet-A	43-->5 (-38)	14-->16 (2)	37-->56 (19)	6-->24 (18)
367	0.0818	Singlet-A	41-->6 (-35)	39-->59 (20)	16-->12 (-4)	4-->23 (19)
348	0.0255	Singlet-A	44-->4 (-40)	13-->17 (4)	37-->73 (36)	6-->6 (0)
326	0.1017	Singlet-A	33-->3 (-30)	23-->28 (5)	31-->64 (33)	13-->6 (-7)
300	0.0910	Singlet-A	16-->4 (-12)	20-->15 (-5)	40-->70 (30)	24-->12 (-12)
296	0.0970	Singlet-A	15-->6 (-9)	46-->59 (13)	15-->17 (2)	24-->18 (-6)
262	0.0371	Singlet-A	18-->20 (2)	48-->60 (12)	17-->18 (1)	16-->2 (-14)

Table S5. Selected transitions for the calculated UV spectrum of complex 4 in Toluene

λ (nm)	Osc. Strength	Symmetry	Major contributions	Minor contributions
461	0	Triplet-A	HOMO->LUMO (52%), HOMO->L+1 (14%)	H-2->LUMO (3%), H-2->L+1 (4%), H-1->LUMO (5%), H-1->L+1 (4%), H-1->L+2 (2%)
402	0.0377	Singlet-A	HOMO->LUMO (34%), HOMO->L+1 (62%)	-
386	0.0370	Singlet-A	H-1->LUMO (35%), H-1->L+1 (62%)	-
368	0.0841	Singlet-A	H-1->L+2 (85%)	HOMO->L+2 (8%), HOMO->L+3 (2%)
344	0.0305	Singlet-A	HOMO->L+3 (92%)	-
328	0.1182	Singlet-A	H-4->LUMO (19%), H-2->LUMO (61%)	H-5->LUMO (7%), H-5->L+1 (2%), H-2->L+1 (2%)
295	0.2458	Singlet-A	H-3->L+2 (66%), H-2->L+2 (12%)	H-5->L+2 (5%), H-4->L+1 (3%), H-4->L+2 (3%)
287	0.0599	Singlet-A	H-5->L+2 (75%)	H-4->L+2 (9%), H-3->L+2 (6%), H-2->L+3 (3%)
281	0.1673	Singlet-A	H-2->L+3 (59%)	H-4->L+1 (9%), H-4->L+2 (2%), H-4->L+3 (8%), H-3->L+3 (4%), H-2->L+4 (3%)
271	0.1825	Singlet-A	H-3->L+3 (41%), H-2->L+4 (39%)	H-4->L+1 (2%), H-4->L+4 (3%)

Table S6. Composition (%) of the selected transitions for the calculated UV-vis spectrum of complex 4

λ (nm)	Osc. Strength	Symmetry	Ir	Ph-Py (1)	Ph-Py(2)	Metallacycle
461	0	Triplet-A	42-->5 (-37)	21-->9 (-12)	32-->43 (11)	6-->43 (37)
402	0.0377	Singlet-A	44-->7 (-37)	16-->5 (-11)	35-->45 (10)	5-->43 (38)
386	0.0370	Singlet-A	40-->7 (-33)	41-->5 (-36)	15-->45 (30)	5-->43 (38)
368	0.0841	Singlet-A	40-->5 (-35)	38-->85 (47)	17-->9 (-8)	5-->1 (-4)
344	0.0305	Singlet-A	44-->3 (-41)	16-->25 (9)	35-->71 (36)	5-->1 (-4)
328	0.1182	Singlet-A	26-->4 (-22)	26-->8 (-18)	33-->43 (10)	16-->45 (29)
295	0.2458	Singlet-A	14-->5 (-9)	48-->84 (36)	21-->9 (-12)	16-->3 (-13)
287	0.0599	Singlet-A	18-->5 (-13)	14-->85 (71)	19-->9 (-10)	49-->1 (-48)
281	0.1673	Singlet-A	26-->4 (-22)	29-->26 (-3)	34-->65 (31)	11-->5 (-6)
271	0.1825	Singlet-A	18-->3 (-15)	43-->48 (5)	26-->48 (22)	12-->2 (-10)

Table S7. Selected transitions for the calculated UV spectrum of complex 6 in THF

λ (nm)	Osc. Strength	Symmetry	Major contributions	Minor contributions
455	0	Triplet-A	H-4->L+1 (10%), HOMO->L+1 (71%)	HOMO->LUMO (6%)
394	0.0316	Singlet-A	HOMO->L+1 (92%)	H-1->L+1 (2%), HOMO->LUMO (3%)
384	0.0669	Singlet-A	H-1->LUMO (97%)	-
369	0.0403	Singlet-A	H-1->L+1 (94%)	-
316	0.0263	Singlet-A	H-1->L+3 (95%)	-
297	0.1077	Singlet-A	H-5->LUMO (17%), H-4->L+1 (56%)	H-4->LUMO (3%), H-3->L+1 (5%), H-3->L+2 (7%), H-2->L+2 (4%)
290	0.0875	Singlet-A	H-5->L+1 (72%)	H-5->LUMO (5%), H-4->L+1 (6%), HOMO->L+4 (2%), HOMO->L+5 (4%)
275	0.1864	Singlet-A	H-3->L+3 (68%)	H-5->LUMO (8%), H-5->L+3 (2%), H-4->L+3 (5%), H-3->L+2 (2%), H-2->L+3 (3%)
273	0.0601	Singlet-A	H-4->L+2 (76%)	H-5->L+2 (5%), H-4->L+3 (2%), H-3->L+2 (6%)
250	0.1486	Singlet-A	H-1->L+6 (49%), H-1->L+8 (18%)	H-8->L+1 (9%), H-7->LUMO (3%), H-7->L+1 (3%)

Table S8. Composition (%) of the selected transitions for the calculated UV-vis spectrum of complex 6

λ (nm)	Osc. Strength	Symmetry	Ir	Ph-Py (1)	Ph-Py(2)	Carbene	Amide
455	0	Triplet-A	42-->4 (-38)	6-->13 (7)	48-->82 (34)	1-->1 (0)	3-->1 (-2)
394	0.0316	Singlet-A	45-->4 (-41)	7-->9 (2)	44-->85 (41)	1-->1 (0)	3-->1 (-2)
384	0.0669	Singlet-A	41-->4 (-37)	45-->88 (43)	5-->8 (3)	4-->0 (-4)	4-->0 (-4)
369	0.0403	Singlet-A	41-->4 (-37)	45-->7 (-38)	5-->87 (82)	4-->1 (-3)	4-->1 (-3)
316	0.0263	Singlet-A	41-->2 (-39)	45-->35 (-10)	5-->62 (57)	4-->0 (-4)	4-->0 (-4)
297	0.1077	Singlet-A	21-->4 (-17)	19-->31 (12)	53-->64 (11)	6-->1 (-5)	1-->1 (0)
290	0.0875	Singlet-A	21-->7 (-14)	58-->11 (-47)	16-->77 (61)	3-->5 (2)	1-->1 (0)
275	0.1864	Singlet-A	45-->2 (-43)	30-->41 (11)	15-->57 (42)	9-->0 (-9)	1-->0 (-1)
273	0.0601	Singlet-A	18-->4 (-14)	9-->61 (52)	69-->34 (-35)	3-->1 (-2)	1-->1 (0)
250	0.1486	Singlet-A	38-->7 (-31)	43-->43 (0)	10-->19 (9)	4-->1 (-3)	5-->30 (25)

Table S9. Selected transitions for the calculated UV spectrum of complex 7 in THF

λ (nm)	Osc. Strength	Symmetry	Major contributions	Minor contributions
453	0	Triplet-A	H-4->L+1 (11%), HOMO->L+1 (72%)	HOMO->LUMO (3%)
392	0.0298	Singlet-A	HOMO->L+1 (91%)	H-1->L+1 (2%), HOMO->LUMO (4%)
385	0.0631	Singlet-A	H-1->LUMO (96%)	-
370	0.0345	Singlet-A	H-1->L+1 (94%)	HOMO->L+1 (2%)
317	0.0241	Singlet-A	H-1->L+3 (95%)	-
298	0.1745	Singlet-A	H-4->L+1 (72%)	H-5->LUMO (3%), H-5->L+1 (2%), H-3->L+1 (7%), H-2->L+2 (3%)
274	0.0500	Singlet-A	H-4->L+2 (74%)	H-5->L+2 (2%), H-4->L+1 (2%), H-4->L+3 (5%), H-3->L+2 (7%)
263	0.0296	Singlet-A	H-4->L+3 (75%)	H-5->L+2 (3%), H-4->L+2 (5%), H-3->L+3 (6%)
249	0.0472	Singlet-A	H-8->L+1 (42%)	H-8->LUMO (3%), H-3->L+5 (4%), H-3->L+6 (6%), H-3->L+7 (5%), H-1->L+8 (8%), H-1->L+9 (3%), H-1->L+10 (6%), HOMO->L+9 (4%)

Table S10. Composition (%) of the selected transitions for the calculated UV-vis spectrum of complex 7

λ (nm)	Osc. Strength	Symmetry	Ir	Ph-Py (1)	Ph-Py(2)	Carbene	Amide
453	0	Triplet-A	42-->3 (-39)	6-->8 (2)	49-->85 (36)	1-->1 (0)	3-->2 (-1)
392	0.0298	Singlet-A	46-->3 (-43)	7-->8 (1)	43-->85 (42)	1-->1 (0)	3-->2 (-1)
385	0.0631	Singlet-A	42-->4 (-38)	44-->90 (46)	4-->6 (2)	5-->0 (-5)	5-->0 (-5)
370	0.0345	Singlet-A	42-->3 (-39)	44-->5 (-39)	5-->88 (83)	4-->1 (-3)	5-->2 (-3)
317	0.0241	Singlet-A	42-->2 (-40)	44-->40 (-4)	4-->51 (47)	5-->0 (-5)	5-->6 (1)
298	0.1745	Singlet-A	19-->3 (-16)	9-->9 (0)	65-->84 (19)	5-->1 (-4)	2-->2 (0)
274	0.0500	Singlet-A	18-->3 (-15)	7-->54 (47)	70-->38 (-32)	2-->1 (-1)	2-->3 (1)
263	0.0296	Singlet-A	18-->2 (-16)	8-->42 (34)	70-->50 (-20)	2-->0 (-2)	2-->6 (4)
249	0.0472	Singlet-A	27-->12 (-15)	27-->15 (-12)	8-->54 (46)	4-->6 (2)	34-->13 (-21)

Table S11. Selected transitions for the calculated UV spectrum of complex 8 in THF

λ (nm)	Osc. Strength	Symmetry	Major contributions	Minor contributions
447	0	Triplet-A	H-2->L+1 (13%),<br>HOMO->L+1 (62%)	H-1->L+1 (9%)
385	0.0231	Singlet-A	HOMO->LUMO (72%),<br>HOMO->L+1 (25%)	-
359	0.0430	Singlet-A	H-1->LUMO (89%)	H-1->L+1 (3%)
297	0.1124	Singlet-A	H-3->LUMO (35%),<br>H-2->L+1 (19%),<br>H-1->L+4 (23%)	H-4->L+1 (4%),<br>H-3->L+1 (4%),<br>H-2->LUMO (4%),<br>H-1->L+3 (4%)
289	0.2895	Singlet-A	H-4->LUMO (45%),<br>H-3->LUMO (18%)	H-4->L+1 (8%),<br>H-3->L+1 (7%),<br>H-3->L+2 (2%),<br>H-1->L+3 (2%),<br>H-1->L+4 (7%)
270	0.0302	Singlet-A	H-2->L+3 (58%)	H-4->L+3 (5%),<br>H-3->L+2 (3%),<br>H-3->L+3 (8%),<br>H-2->L+2 (5%),<br>H-2->L+4 (6%),<br>H-1->L+3 (4%)
258	0.0493	Singlet-A	H-7->LUMO (49%),<br>HOMO->L+6 (27%)	H-6->LUMO (4%),<br>H-4->L+2 (3%),<br>H-4->L+3 (2%),<br>HOMO->L+7 (4%)
247	0.0252	Singlet-A	H-5->L+2 (44%),<br>H-1->L+6 (24%)	H-8->LUMO (4%),<br>H-7->L+1 (2%),<br>H-6->L+3 (3%),<br>H-4->L+4 (4%),<br>HOMO->L+8 (7%)

Table S12. Composition (%) of the selected transitions for the calculated UV-vis spectrum of complex 8

λ (nm)	Osc. Strength	Symmetry	Ir	Ph-Py (1)	Ph-Py(2)	Carbene	Amide
447	0	Triplet-A	37-->3 (-34)	19-->0 (-19)	37-->92 (55)	1-->4 (3)	5-->1 (-4)
385	0.0231	Singlet-A	44-->2 (-42)	16-->69 (53)	32-->25 (-7)	1-->3 (2)	7-->0 (-7)
359	0.0430	Singlet-A	32-->2 (-30)	46-->89 (43)	20-->5 (-15)	1-->3 (2)	0-->0 (0)
297	0.1124	Singlet-A	19-->4 (-15)	47-->47 (0)	30-->38 (8)	1-->10 (9)	2-->0 (-2)
289	0.2895	Singlet-A	43-->3 (-40)	37-->69 (32)	11-->22 (11)	7-->6 (-1)	2-->0 (-2)
270	0.0302	Singlet-A	15-->11 (-4)	23-->20 (-3)	58-->23 (-35)	1-->46 (45)	3-->0 (-3)
258	0.0493	Singlet-A	33-->4 (-29)	33-->63 (30)	20-->6 (-14)	7-->3 (-4)	8-->24 (16)
247	0.0252	Singlet-A	20-->5 (-15)	25-->40 (15)	30-->30 (0)	5-->3 (-2)	20-->22 (2)

Table S13. Selected transitions for the calculated UV spectrum of complex 9 in THF

λ (nm)	Osc. Strength	Symmetry	Major contributions	Minor contributions
494	0	Triplet-A	HOMO->LUMO (59%), HOMO->L+1 (27%)	H-3->L+1 (4%), HOMO->L+2 (2%)
457	0.0596	Singlet-A	HOMO->LUMO (87%), HOMO->L+1 (10%)	-
430	0.0374	Singlet-A	HOMO->LUMO (11%), HOMO->L+1 (85%)	-
400	0.0488	Singlet-A	HOMO->L+2 (92%)	HOMO->L+1 (2%), HOMO->L+3 (3%)
359	0.0559	Singlet-A	H-2->LUMO (25%), H-2->L+1 (10%), H-1->L+1 (52%)	HOMO->L+4 (7%)
347	0.0330	Singlet-A	H-2->L+1 (71%), H-1->L+1 (16%)	H-2->L+2 (4%)
279	0.1247	Singlet-A	H-4->L+1 (12%), H-3->L+3 (10%), HOMO->L+8 (49%)	H-5->L+1 (5%), H-5->L+2 (3%), HOMO->L+9 (3%)
273	0.0373	Singlet-A	H-3->L+4 (66%)	H-6->L+1 (3%), H-6->L+2 (2%), H-1->L+6 (9%), HOMO->L+10 (5%)
272	0.2051	Singlet-A	H-7->LUMO (34%), H-1->L+5 (12%), H-1->L+6 (28%)	H-3->L+4 (7%), HOMO->L+11 (2%)

Table S14. Composition (%) of the selected transitions for the calculated UV-vis spectrum of complex 9

λ (nm)	Osc. Strength	Symmetry	Ir	Ph-Py	Ph-Py (6tt')	CH_2-Ph (6tt')	Amidure (6tt')	C_q (6tt')
494	0	Triplet-A	45-->3 (-42)	26-->34 (8)	3-->56 (53)	23-->1 (-22)	2-->7 (5)	0-->0 (0)
457	0.0596	Singlet-A	47-->2 (-45)	25-->24 (-1)	3-->71 (68)	23-->1 (-22)	2-->3 (1)	0-->0 (0)
430	0.0374	Singlet-A	47-->4 (-43)	25-->59 (34)	3-->27 (24)	23-->1 (-22)	2-->9 (7)	0-->0 (0)
400	0.0488	Singlet-A	47-->2 (-45)	25-->14 (-11)	3-->7 (4)	23-->1 (-22)	2-->77 (75)	0-->0 (0)
359	0.0559	Singlet-A	72-->4 (-68)	11-->53 (42)	7-->35 (28)	6-->1 (-5)	1-->7 (6)	2-->0 (-2)
347	0.0330	Singlet-A	77-->4 (-73)	9-->62 (53)	4-->20 (16)	5-->1 (-4)	2-->13 (11)	2-->0 (-2)
279	0.1247	Singlet-A	33-->4 (-29)	30-->21 (-9)	6-->56 (50)	29-->13 (-16)	2-->6 (4)	1-->1 (0)
273	0.0373	Singlet-A	12-->4 (-8)	49-->55 (6)	4-->33 (29)	32-->5 (-27)	1-->3 (2)	1-->0 (-1)
272	0.2051	Singlet-A	37-->6 (-31)	12-->14 (2)	39-->61 (22)	8-->2 (-6)	2-->16 (14)	3-->0 (-3)

Table S15. Selected transitions for the calculated UV spectrum of complex 10 in THF

λ (nm)	Osc. Strength	Symmetry	Major contributions	Minor contributions
494	0	Triplet-A	HOMO->LUMO (56%), HOMO->L+1 (33%)	H-3->L+1 (4%)
457	0.0564	Singlet-A	HOMO->LUMO (86%), HOMO->L+1 (12%)	-
431	0.0410	Singlet-A	HOMO->LUMO (13%), HOMO->L+1 (84%)	-
383	0.0258	Singlet-A	H-1->LUMO (77%), HOMO->L+2 (19%)	-
360	0.0550	Singlet-A	H-2->LUMO (24%), H-2->L+1 (14%), H-1->L+1 (51%)	HOMO->L+3 (7%)
348	0.0427	Singlet-A	H-2->L+1 (73%), H-1->L+1 (19%)	-
307	0.0757	Singlet-A	H-4->LUMO (15%), H-3->L+1 (40%), H-2->L+3 (16%), H-1->L+3 (17%)	H-5->LUMO (6%)
272	0.1766	Singlet-A	H-7->LUMO (19%), H-3->L+3 (57%)	H-6->L+1 (3%), H-1->L+4 (4%), HOMO->L+8 (3%)

Table S16. Composition (%) of the selected transitions for the calculated UV-vis spectrum of complex 10

λ (nm)	Osc. Strength	Symmetry	Ir	Ph-Py	Ph-Py (6tt’)	CH_2-Ph (6tt’)	Amidure (6tt’)	C_q (6tt’)
494	0	Triplet-A	46-->3 (-43)	26-->41 (15)	3-->55 (52)	23-->1 (-22)	1-->0 (-1)	0-->0 (0)
457	0.0564	Singlet-A	48-->2 (-46)	25-->24 (-1)	3-->72 (69)	23-->1 (-22)	1-->0 (-1)	0-->0 (0)
431	0.0410	Singlet-A	48-->4 (-44)	25-->69 (44)	3-->25 (22)	23-->1 (-22)	1-->0 (-1)	0-->0 (0)
383	0.0258	Singlet-A	66-->2 (-64)	14-->20 (6)	9-->77 (68)	8-->1 (-7)	0-->0 (0)	2-->0 (-2)
360	0.0550	Singlet-A	73-->4 (-69)	11-->61 (50)	7-->34 (27)	6-->1 (-5)	1-->0 (-1)	2-->0 (-2)
348	0.0427	Singlet-A	78-->5 (-73)	9-->77 (68)	4-->17 (13)	5-->1 (-4)	2-->0 (-2)	2-->0 (-2)
307	0.0757	Singlet-A	30-->3 (-27)	35-->60 (25)	6-->35 (29)	27-->1 (-26)	1-->0 (-1)	2-->0 (-2)
272	0.1766	Singlet-A	8-->3 (-5)	41-->51 (10)	20-->42 (22)	28-->3 (-25)	2-->1 (-1)	2-->0 (-2)

Theoretical Analysis of Molecular Orbitals of Complexes 2-10

Energies and population analysis (%) of frontier molecular orbitals are given in Tables S17–S25 whereas Figures S9–S17 collects the frontier molecular orbitals.

Table S17. Composition of the frontier orbital of complex 2

MO	eV	Iridium	Ph-Py (1)	Ph-Py(2)	Metallacycle
L+9	0.53	7	54	38	1
L+8	0.44	9	35	53	3
L+7	0.22	14	1	2	83
L+6	0.12	84	3	2	11
L+5	-0.09	1	0	0	98
L+4	-0.61	2	72	26	0
L+3	-0.82	3	25	71	1
L+2	-1.17	5	80	14	1
L+1	-1.28	3	15	80	1
LUMO	-1.75	7	1	2	90
HOMO	-5.18	43	12	38	6
H-1	-5.26	41	43	11	5
H-2	-5.86	29	25	34	12
H-3	-6	12	65	9	13
H-4	-6.09	34	5	39	22
H-5	-6.16	15	10	22	52
H-6	-6.7	14	40	6	40
H-7	-6.83	20	16	52	11
H-8	-6.89	1	1	1	96
H-9	-7.04	15	28	36	21

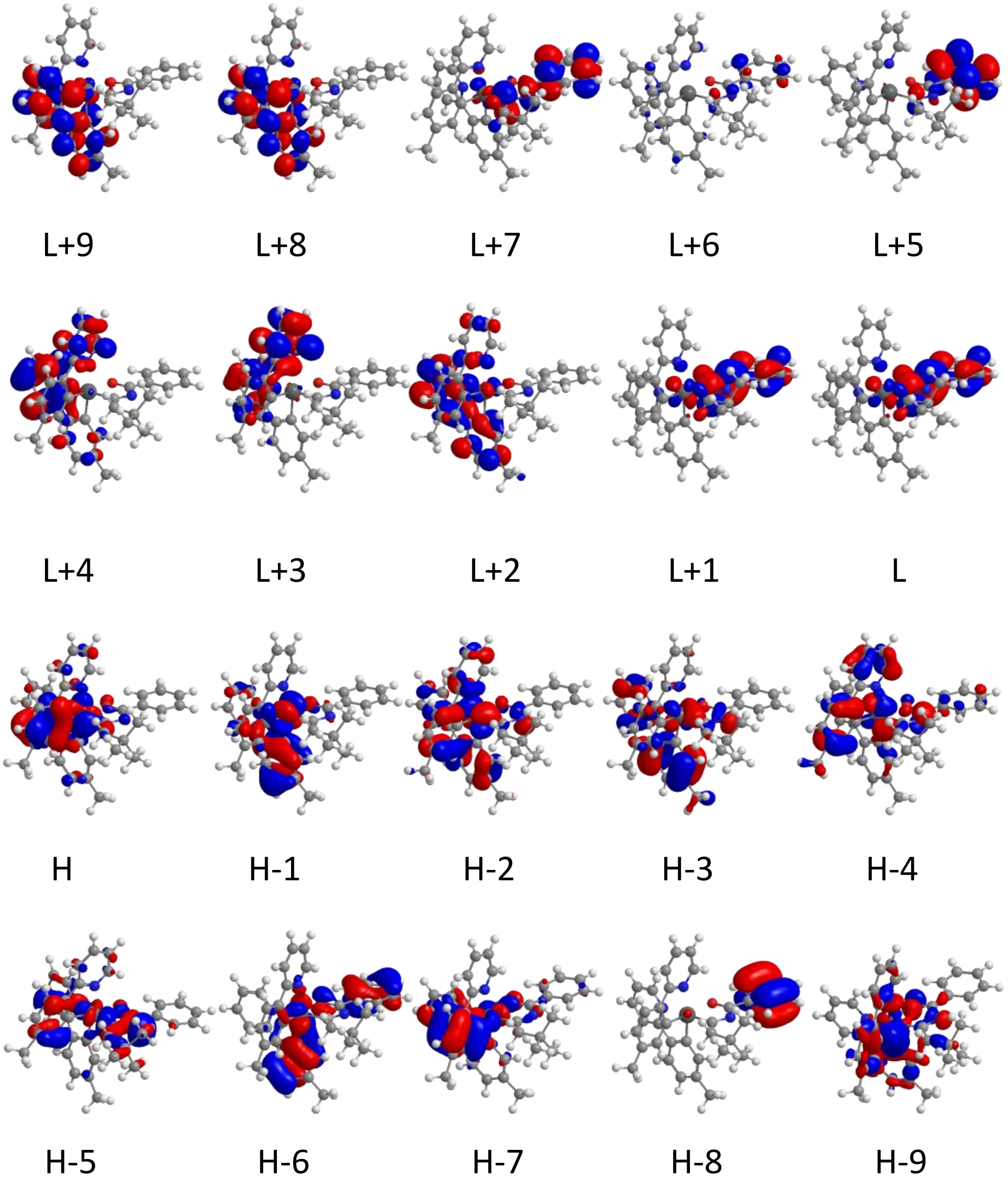

Figure S12. Molecular orbitals of complex **2** (isovalue 0.003 au).

Table S18. Composition of the frontier orbital of complex 3

MO	eV	Iridium	Ph-Py (1)	Ph-Py(2)	Metallacycle
L+9	1.2	22	51	25	2
L+8	1.04	8	23	69	1
L+7	0.64	7	62	29	1
L+6	0.52	8	28	63	1
L+5	0.12	94	2	2	3
L+4	-0.6	2	80	17	1
L+3	-0.83	4	17	73	6
L+2	-1.07	9	15	10	66
L+1	-1.14	5	74	11	10
LUMO	-1.3	2	8	83	6
HOMO	-5.09	44	13	37	6
H-1	-5.17	40	44	12	4
H-2	-5.79	28	28	32	12
H-3	-5.91	10	58	10	21
H-4	-6.04	37	15	13	35
H-5	-6.05	16	7	52	26
H-6	-6.73	22	13	54	11
H-7	-6.78	31	56	6	7
H-8	-6.94	15	26	36	23
H-9	-7.23	19	32	29	21

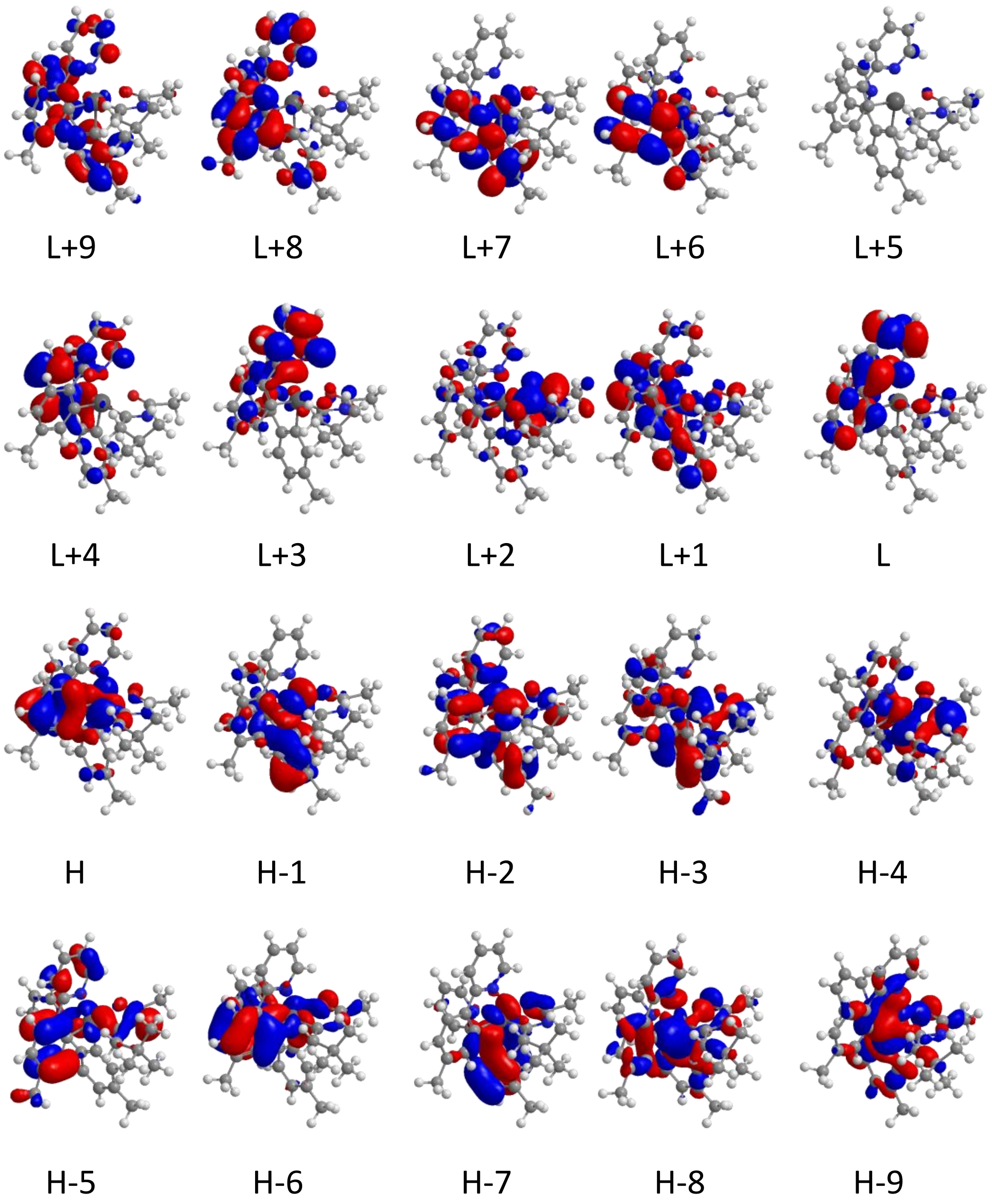

Figure S13. Molecular orbitals of complex **3** (isovalue 0.003 au).

Table S19. Composition of the frontier orbital of complex 4

MO	eV	Iridium	Ph-Py (1)	Ph-Py(2)	Metallacycle
L+9	0.65	8	61	30	1
L+8	0.55	9	28	62	1
L+7	0.21	93	3	1	3
L+6	0.03	3	0	2	96
L+5	-0.07	1	0	0	98
L+4	-0.58	2	73	25	1
L+3	-0.79	3	25	71	1
L+2	-1.12	5	86	8	1
L+1	-1.18	9	3	47	42
LUMO	-1.3	4	8	43	45
HOMO	-5.07	44	16	35	5
H-1	-5.15	40	41	15	5
H-2	-5.79	23	34	34	10
H-3	-5.91	11	57	18	15
H-4	-6.02	40	7	38	15
H-5	-6.07	16	11	16	57
H-6	-6.55	3	8	7	82
H-7	-6.73	22	17	41	19
H-8	-6.78	9	8	14	69
H-9	-6.81	25	40	5	30

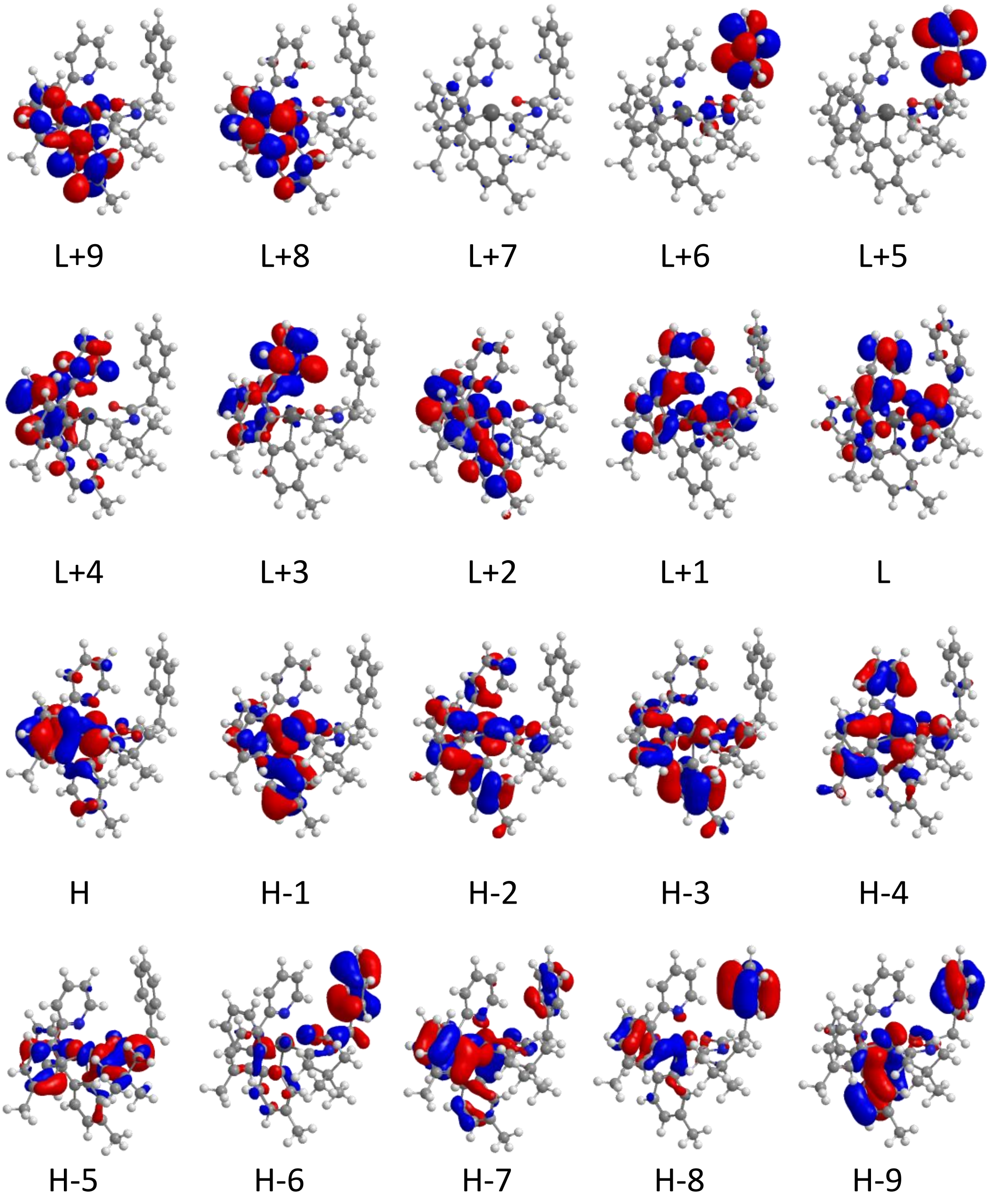

Figure S14. Molecular orbitals of complex **4** (isovalue 0.003 au).

Table S20. Composition of the frontier orbital of complex 5

MO	eV	Iridium	Ph-Py (1)	Ph-Py(2)	Metallacycle
L+9	1.05	17	43	37	3
L+8	0.89	12	26	61	1
L+7	0.43	9	54	36	1
L+6	0.32	13	31	56	1
L+5	0.09	90	5	3	2
L+4	-0.68	2	75	23	0
L+3	-0.9	3	22	74	1
L+2	-1.27	5	87	7	1
L+1	-1.38	4	8	87	1
LUMO	-1.82	11	2	4	84
HOMO	-5.39	39	17	40	4
H-1	-5.46	35	46	16	3
H-2	-6.01	15	51	31	3
H-3	-6.14	10	36	50	4
H-4	-6.37	50	11	21	18
H-5	-6.51	14	14	4	68
H-6	-6.99	27	16	50	7
H-7	-7.08	36	50	8	6
H-8	-7.23	15	39	31	14
H-9	-7.56	17	16	38	29

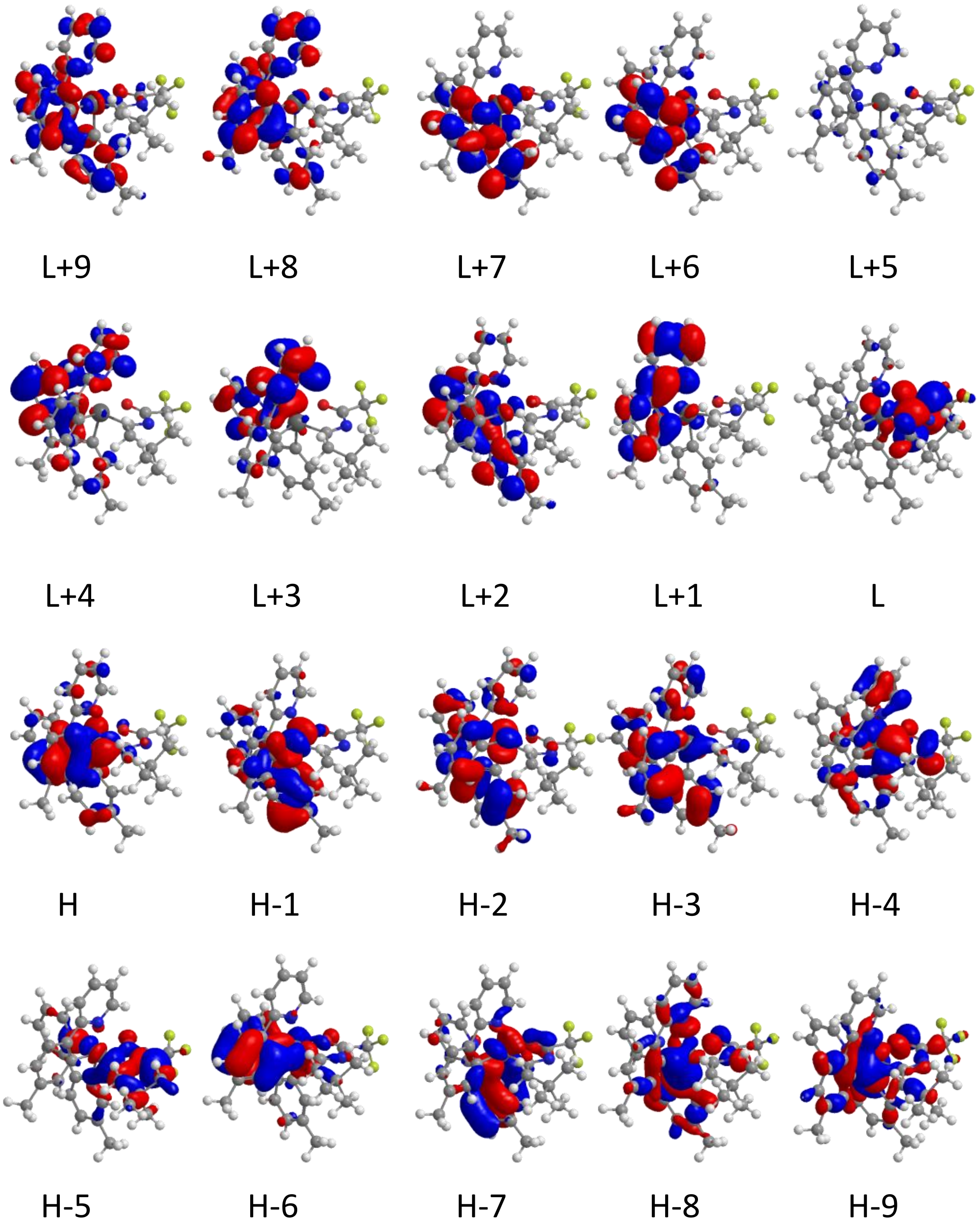

Figure S15. Molecular orbitals of complex **5** (isovalue 0.003 au).

Table S21. Composition of the frontier orbital of complex 6

MO	eV	Iridium	Ph-Py (1)	Ph-Py(2)	Carbene	Amide
L+9	1.04	7	67	24	1	2
L+8	0.66	12	33	24	1	30
L+7	0.57	6	15	67	0	12
L+6	0.44	7	53	1	0	39
L+5	0.27	29	1	3	65	2
L+4	0.03	80	1	2	15	3
L+3	-0.6	2	35	62	0	0
L+2	-0.81	4	61	34	1	1
L+1	-1.16	4	7	87	1	1
LUMO	-1.24	4	88	8	0	0
HOMO	-5.08	46	6	45	1	3
H-1	-5.2	41	45	5	4	4
H-2	-5.61	13	3	11	66	6
H-3	-5.76	52	28	12	8	1
H-4	-5.9	16	4	76	2	1
H-5	-6.03	20	66	10	4	1
H-6	-6.62	16	29	34	1	20
H-7	-6.77	27	37	29	5	2
H-8	-6.96	17	35	39	2	7
H-9	-7.21	18	41	27	1	13

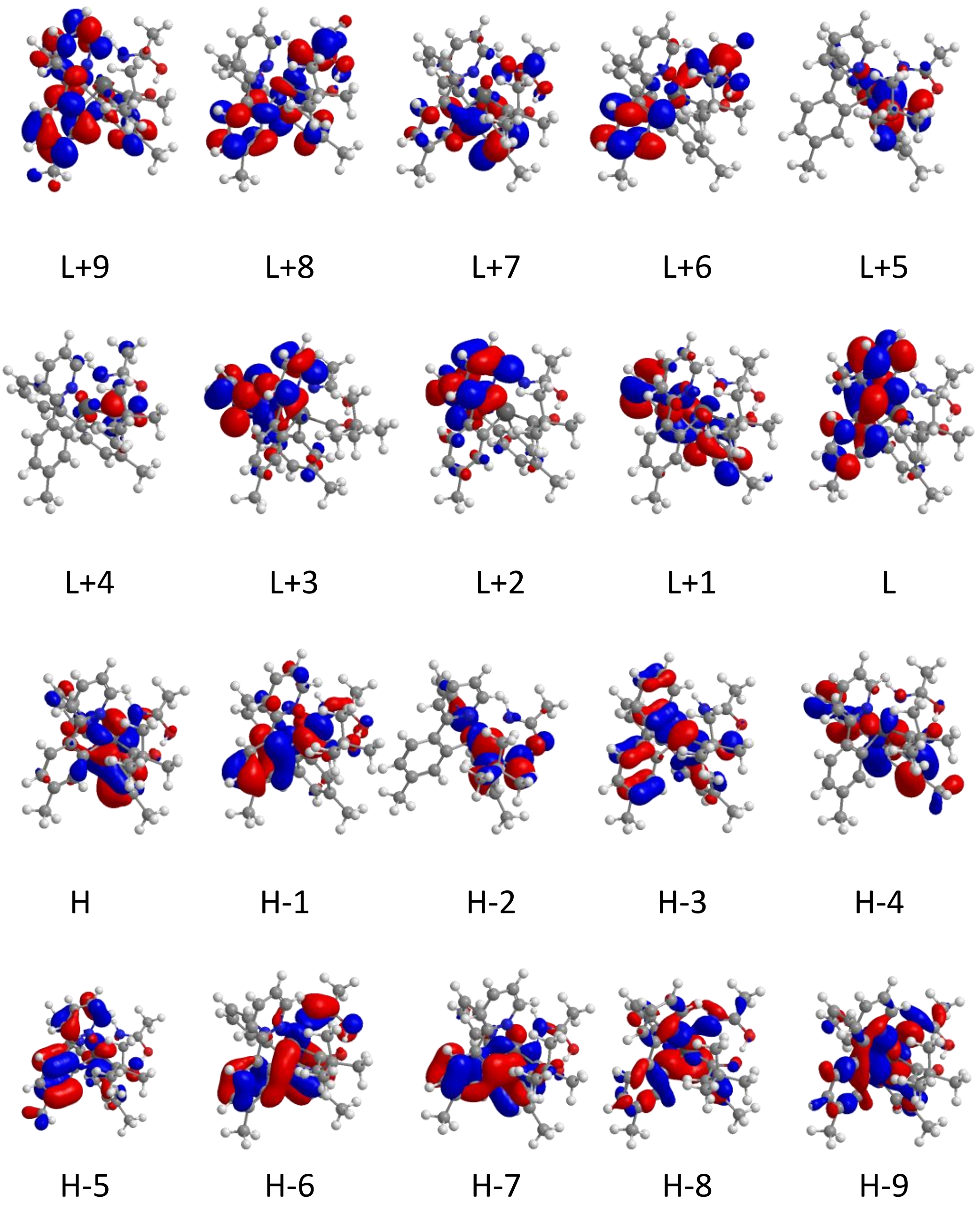

Figure S16. Molecular orbitals of complex **6** (isovalue 0.003 au).

Table S22. Composition of the frontier orbital of complex 7

MO	eV	Iridium	Ph-Py (1)	Ph-Py(2)	Carbene	Amide
L+9	0.59	11	7	69	1	12
L+8	0.48	10	60	3	1	26
L+7	0.29	36	1	4	49	11
L+6	0.1	64	2	3	28	3
L+5	-0.09	2	1	7	1	89
L+4	-0.11	1	0	2	1	96
L+3	-0.61	2	40	51	0	6
L+2	-0.81	3	56	36	1	3
L+1	-1.15	3	5	88	1	2
LUMO	-1.23	4	90	6	0	0
HOMO	-5.08	46	6	44	1	3
H-1	-5.18	42	44	4	5	5
H-2	-5.64	13	3	11	65	7
H-3	-5.76	51	27	12	8	1
H-4	-5.87	16	4	77	2	2
H-5	-6.02	20	66	8	4	2
H-6	-6.51	8	24	22	1	45
H-7	-6.68	11	7	27	2	53
H-8	-6.78	11	22	5	2	60
H-9	-6.81	12	14	19	2	52

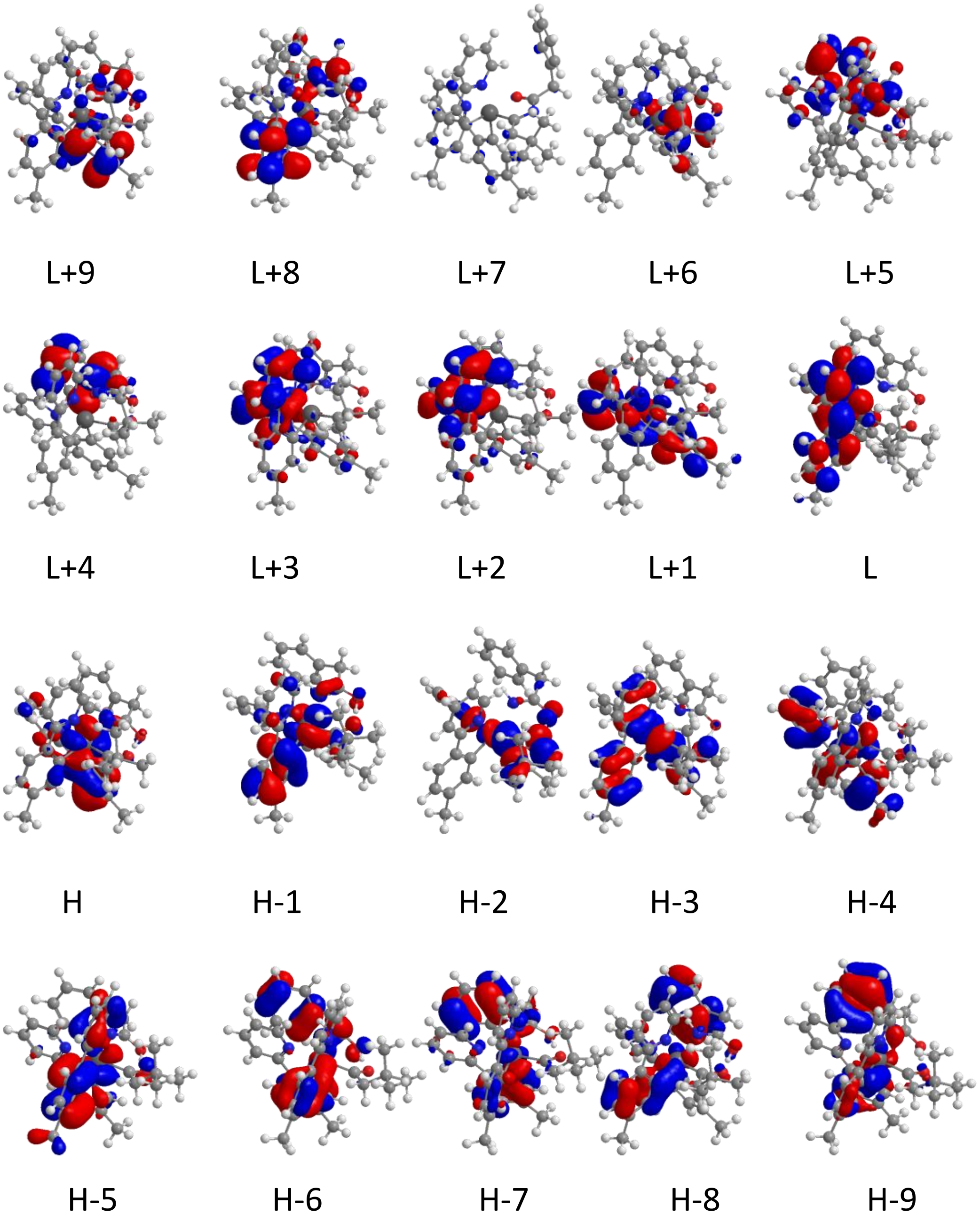

Figure S17. Molecular orbitals of complex **7** (isovalue 0.003 au).

Table S23. Composition of the frontier orbital of complex 8

MO	eV	Iridium	Ph-Py (1)	Ph-Py(2)	Carbene	Amide
L+9	0.95	7	52	40	1	0
L+8	0.47	6	39	47	0	7
L+7	0.37	5	42	47	0	5
L+6	0.22	7	14	1	1	79
L+5	0.14	93	1	1	2	3
L+4	-0.62	8	33	37	22	0
L+3	-0.78	12	16	20	52	0
L+2	-0.88	3	53	43	0	0
L+1	-1.31	3	0	92	4	1
LUMO	-1.34	2	92	2	3	0
HOMO	-5.29	44	16	32	1	7
H-1	-5.51	32	46	20	1	0
H-2	-6.04	11	14	72	0	3
H-3	-6.11	10	71	14	2	3
H-4	-6.34	62	18	9	10	1
H-5	-6.51	7	14	39	6	35
H-6	-6.87	11	1	18	40	29
H-7	-6.96	25	47	13	8	7
H-8	-7.17	23	31	32	2	11
H-9	-7.45	20	34	34	3	9

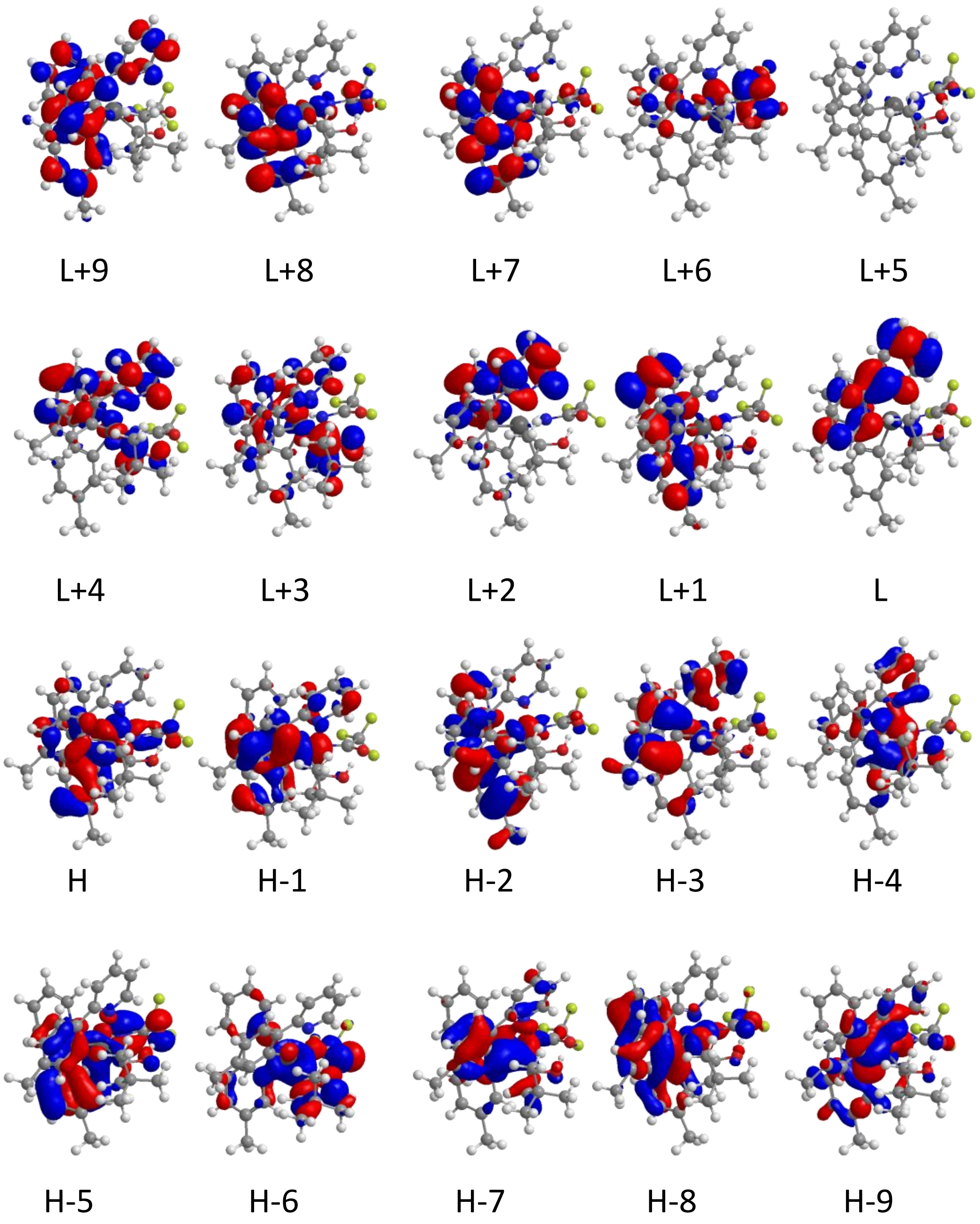

Figure S18. Molecular orbitals of complex **8** (isovalue 0.003 au).

Table S24. Composition of the frontier orbital of complex 9

MO	eV	Iridium	Ph-Py	Ph-Py (6tt')	CH_2-Ph (6tt')	Amidure (6tt')	C_q (6tt')
L+9	0.59	7	68	2	20	2	0
L+8	0.46	4	2	72	20	1	1
L+7	0.21	88	1	9	1	1	0
L+6	-0.04	12	2	80	2	4	1
L+5	-0.22	1	0	0	0	99	0
L+4	-0.56	3	67	29	1	0	0
L+3	-0.75	2	29	64	0	4	0
L+2	-0.96	2	12	5	1	81	0
L+1	-1.07	4	65	20	1	10	0
LUMO	-1.26	2	19	76	1	2	0
HOMO	-4.65	47	25	3	23	2	0
H-1	-5.29	70	12	11	5	0	2
H-2	-5.46	78	8	3	6	2	2
H-3	-5.66	2	57	1	37	1	1
H-4	-5.95	9	43	11	34	1	2
H-5	-6.01	11	6	21	51	6	5
H-6	-6.07	6	32	23	29	6	4
H-7	-6.37	3	4	81	5	4	4
H-8	-6.82	12	4	34	33	17	1
H-9	-6.86	4	11	5	6	74	1

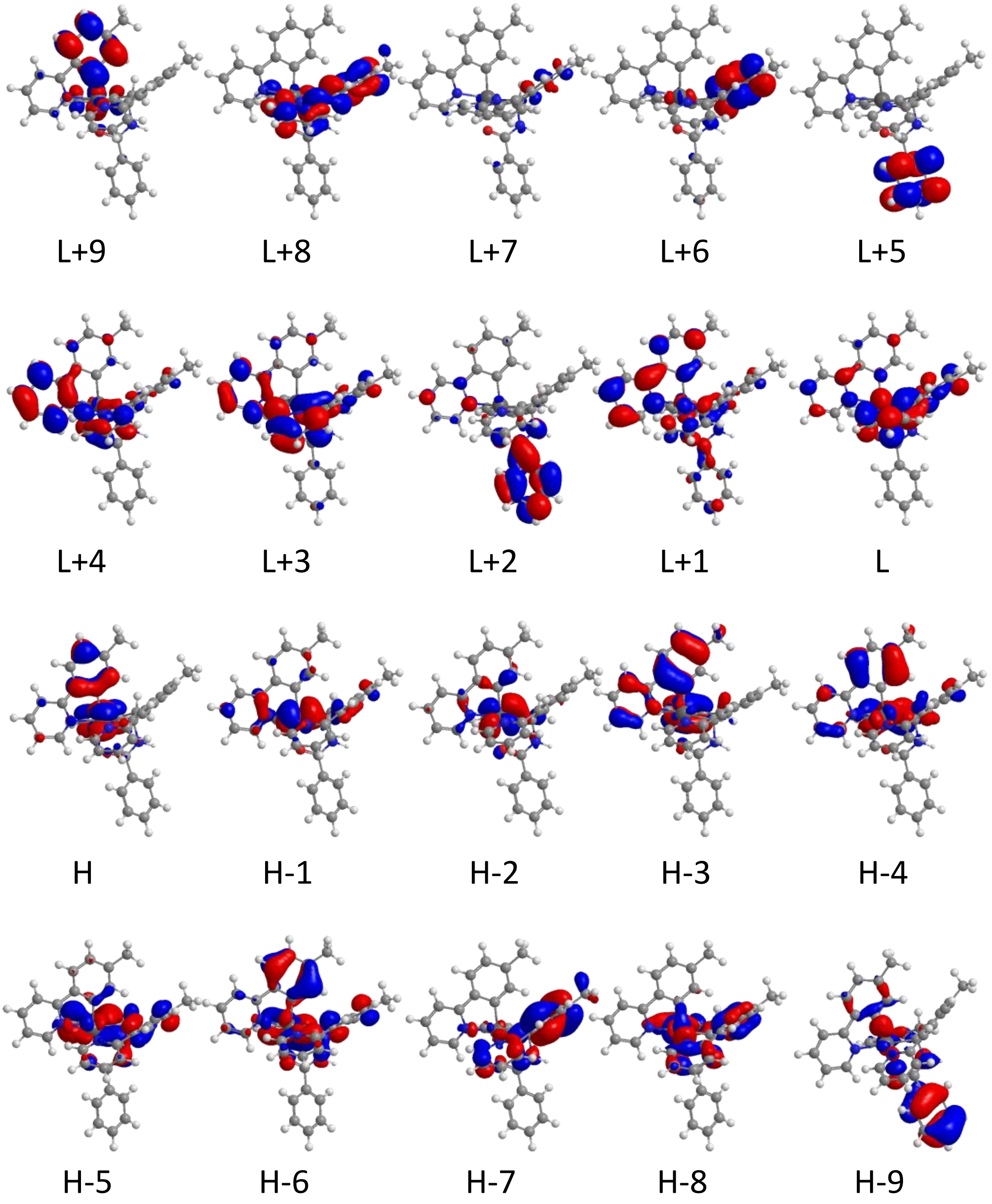

Figure S19. Molecular orbitals of complex **9** (isovalue 0.003 au).

Table S25. Composition of the frontier orbital of complex 10

MO	eV	Iridium	Ph-Py	Ph-Py (6tt')	CH_2-Ph (6tt')	Amidure (6tt')	C_q (6tt')
L+9	0.72	9	18	2	35	36	0
L+8	0.63	5	11	24	58	2	0
L+7	0.51	7	25	32	12	23	0
L+6	0.45	4	13	44	19	18	1
L+5	0.15	84	1	9	1	5	0
L+4	-0.09	14	2	73	2	8	0
L+3	-0.55	3	66	29	1	0	0
L+2	-0.76	3	30	66	0	1	0
L+1	-1.05	5	77	17	1	0	0
LUMO	-1.25	2	17	80	1	0	0
HOMO	-4.64	48	25	3	23	1	0
H-1	-5.28	71	12	10	5	0	2
H-2	-5.44	80	8	3	5	2	2
H-3	-5.65	2	57	1	38	1	0
H-4	-5.95	9	44	11	33	1	2
H-5	-6.02	9	8	18	58	4	4
H-6	-6.07	7	29	30	22	7	5
H-7	-6.37	3	4	80	5	4	4
H-8	-6.78	13	8	28	29	21	2
H-9	-6.9	12	28	18	23	10	9

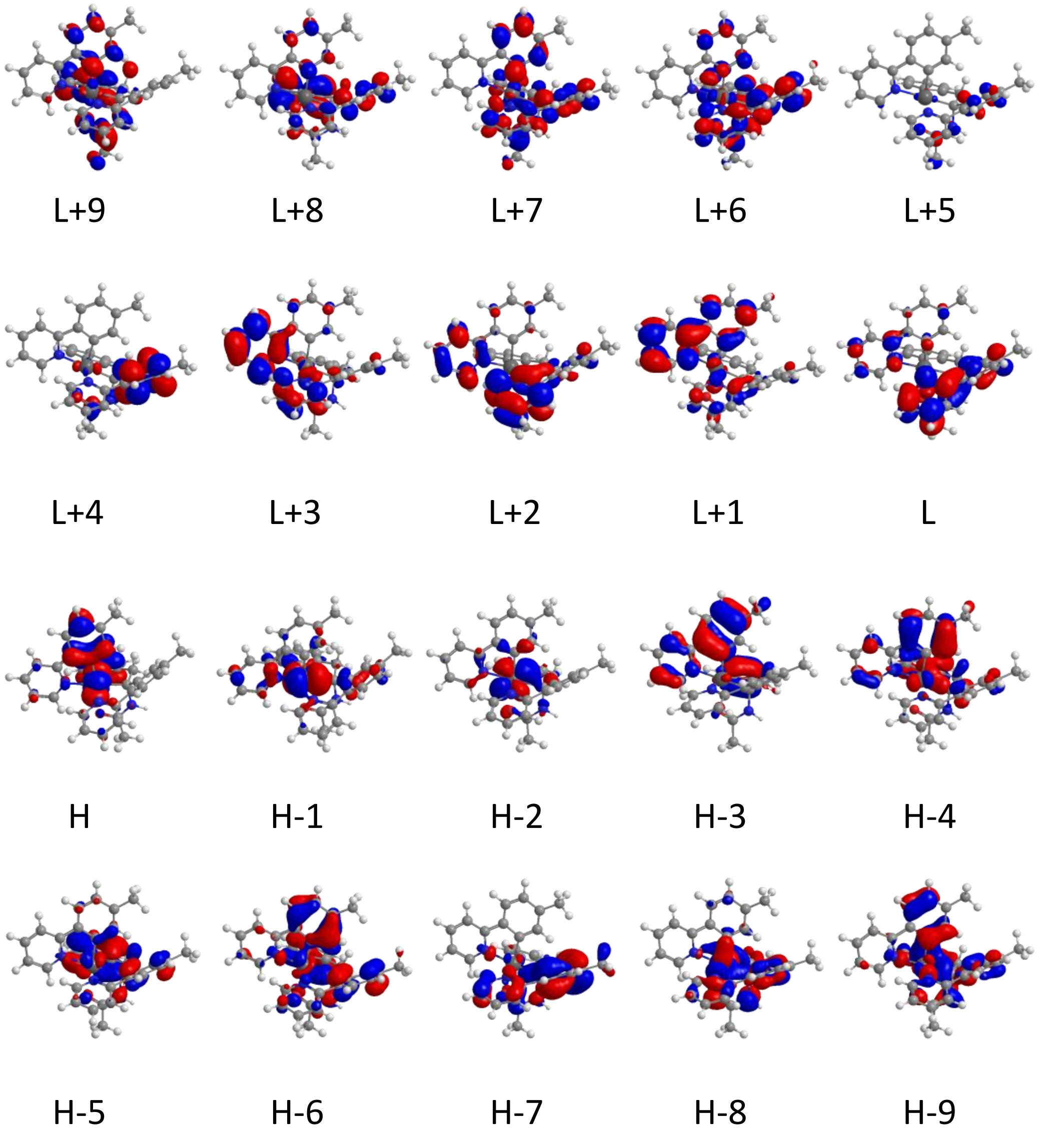

Figure S20. Molecular orbitals of complex **10** (isovalue 0.003 au).

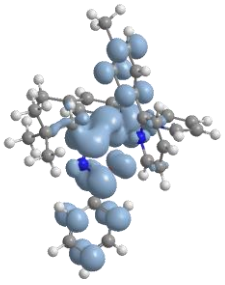

2

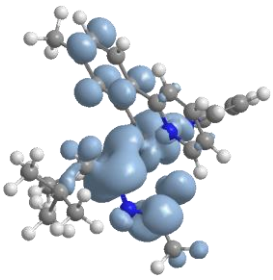

3

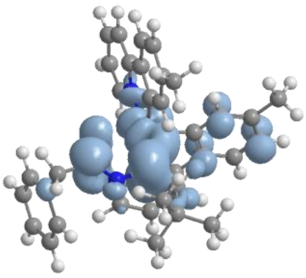

4

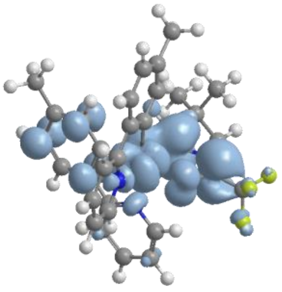

5

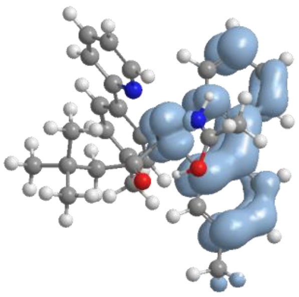

6

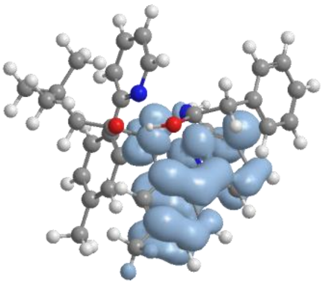

7

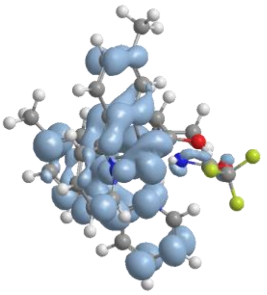

8

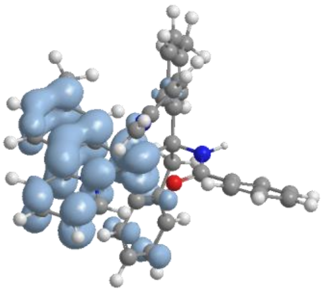

9

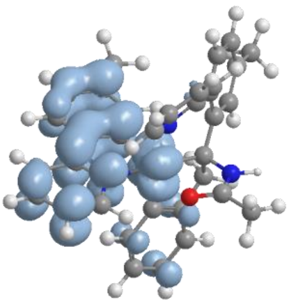

10

Figure S21. Spin density distributions for the optimized triplet T_1 of complexes **2-10** (0.002 isovalue).

Cyclic Voltammograms of Complexes 2 and 6-10

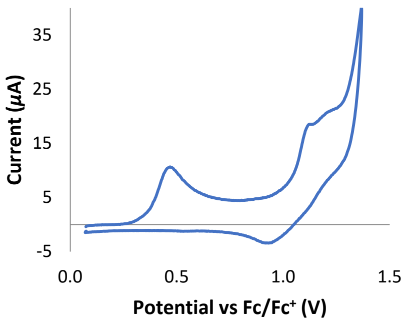

2

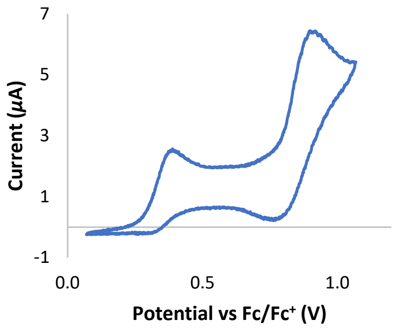

6

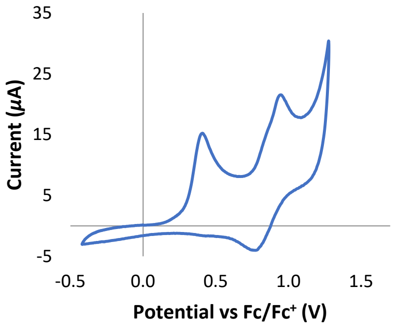

7

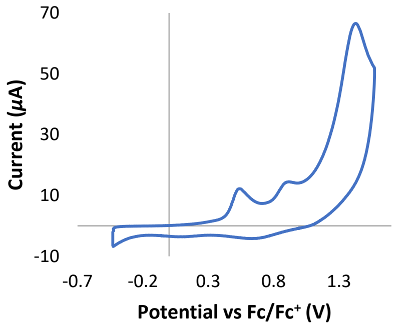

8

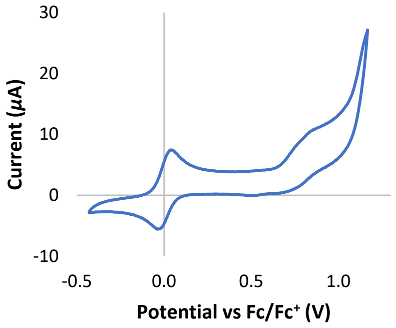

9

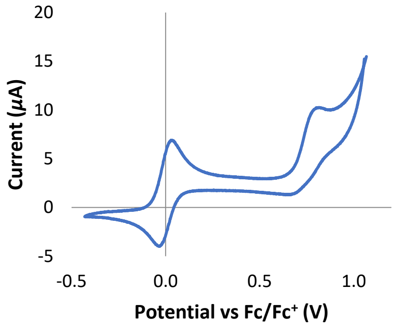

10

Figure S22. Cyclic voltammograms of complexes **2, 6-10** in 10^{-3} M solutions with Bu_4NPF_6 as supporting electrolyte (0.1 M) at a scan rate of 250 mV s^{-1}. The potentials were referenced to the ferrocene/ferrocenium (Fc/Fc^+) couple.

Normalized Emission and Excitation Spectra of 2-4 and 6-10

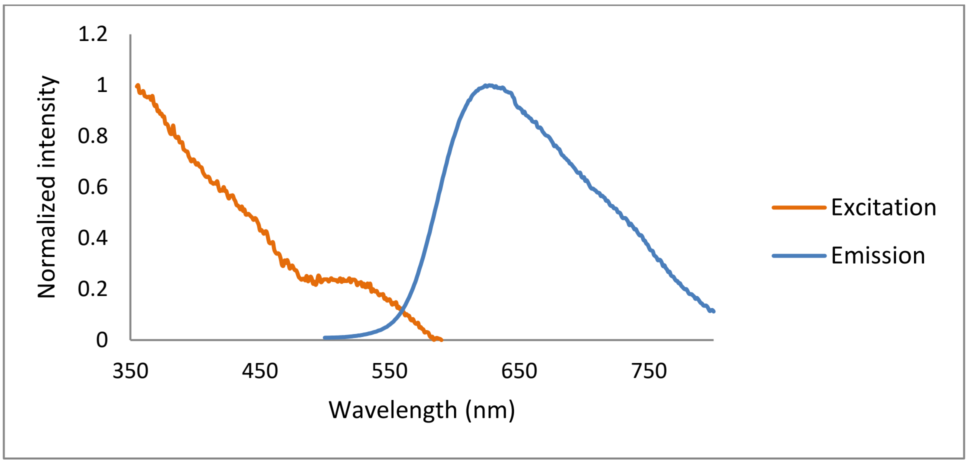

Figure S23. Normalized emission and excitation spectrum of complex **2** in PMMA film (5 wt %) at 298 K.

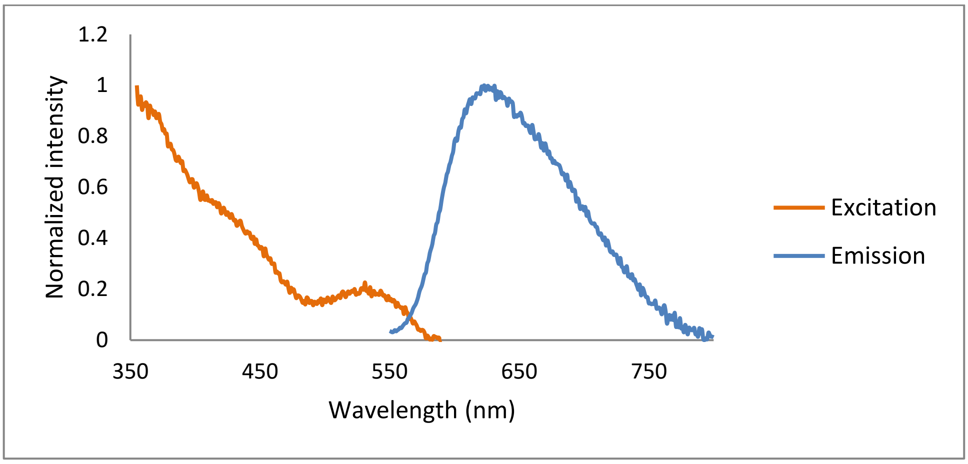

Figure S24. Normalized emission and excitation spectrum for a 1 x 10^{-5} M solution of complex **2** in 2-MeTHF at 298 K.

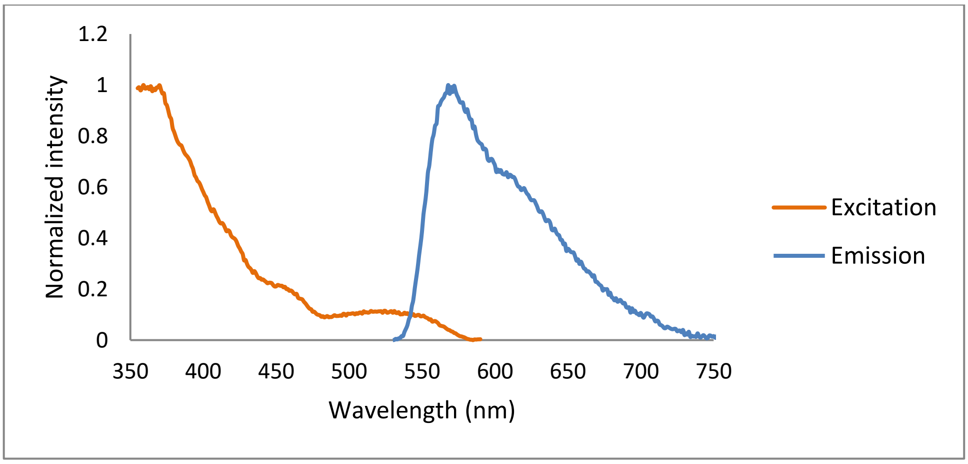

Figure S25. Normalized emission and excitation spectrum for a 1 x 10^{-5} M solution of complex **2** in 2-MeTHF at 77 K.

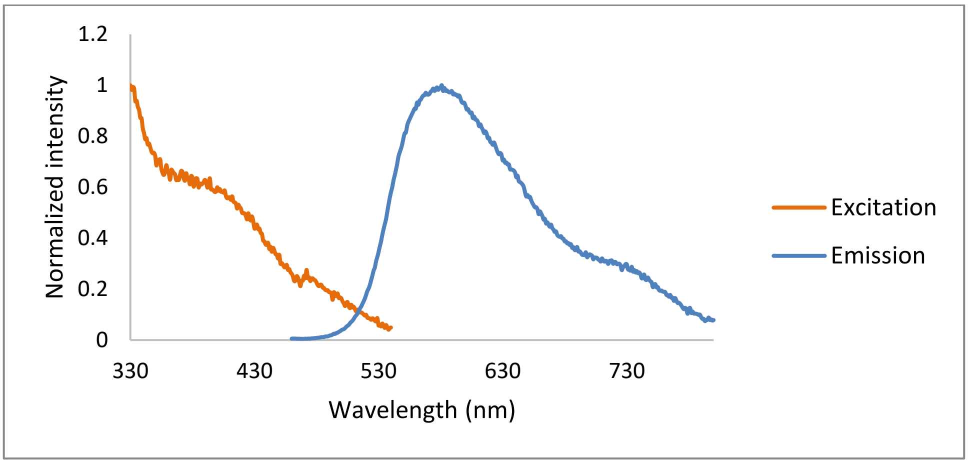

Figure S26. Normalized emission and excitation spectrum of complex **3** in PMMA film (5 wt %) at 298 K.

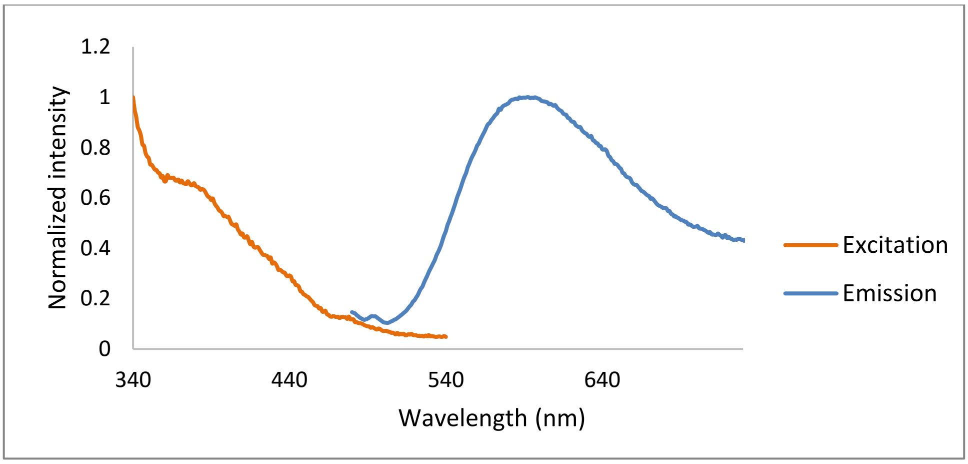

Figure S27. Normalized emission and excitation spectrum for a 1 x 10^{-5} M solution of complex **3** in toluene at 298 K.

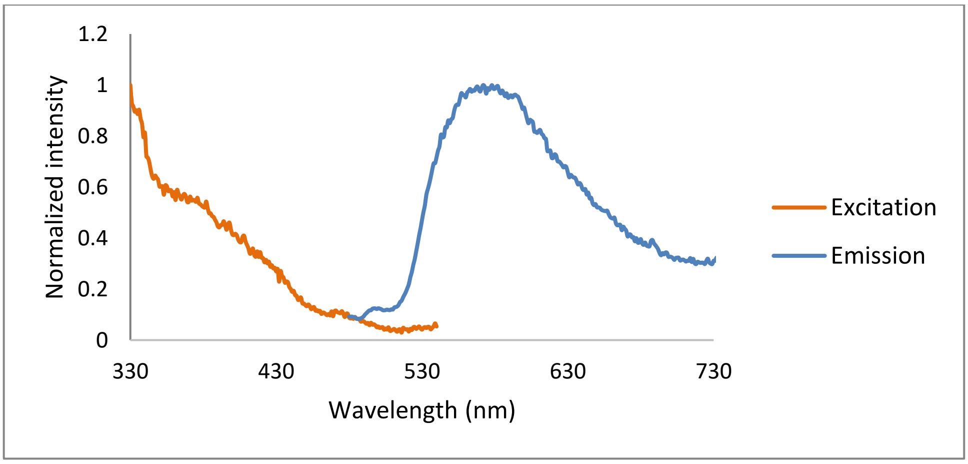

Figure S28. Normalized emission and excitation spectrum for a 1 x 10^{-5} M solution of complex **3** in toluene at 77 K.

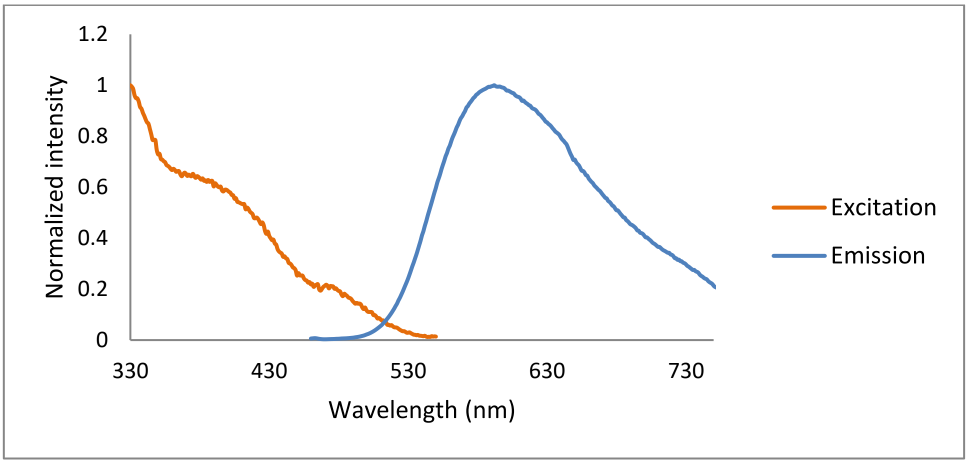

Figure S29. Normalized emission and excitation spectrum of complex **4** in PMMA film (5 wt %) at 298 K.

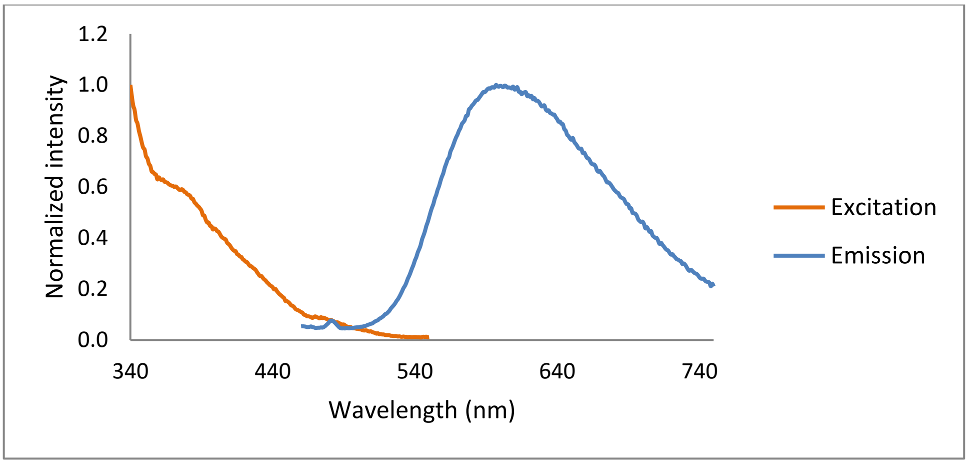

Figure S30. Normalized emission and excitation spectrum for a 1 x 10^{-5} M solution of complex **4** in toluene at 298 K.

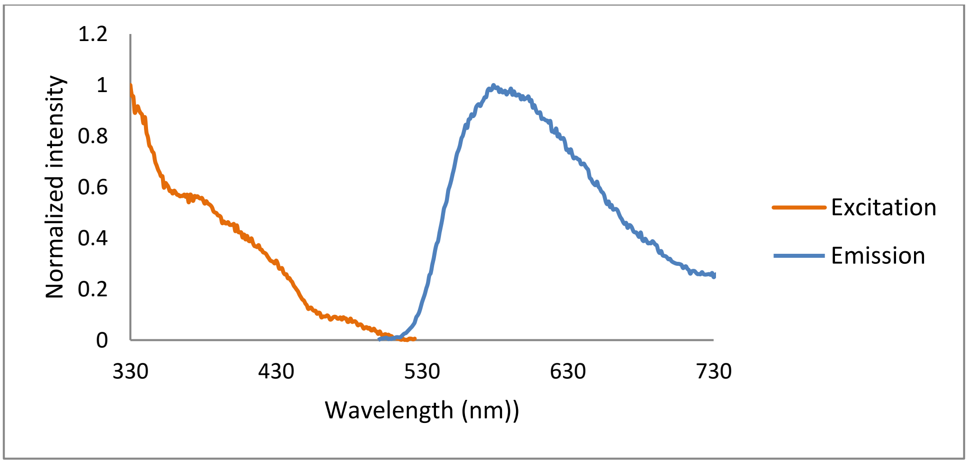

Figure S31. Normalized emission and excitation spectrum for a 1 x 10^{-5} M solution of complex **4** in toluene at 77 K.

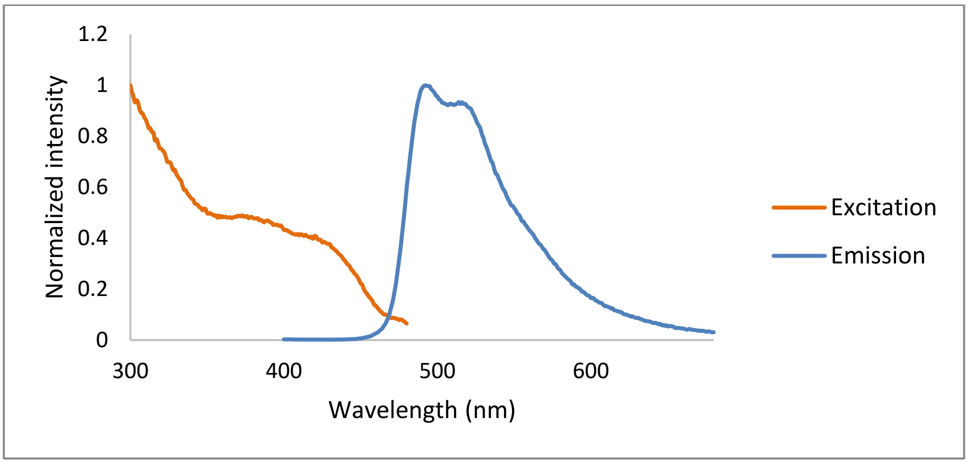

Figure S32. Normalized emission and excitation spectrum of complex **6** in PMMA film (5 wt %) at 298 K.

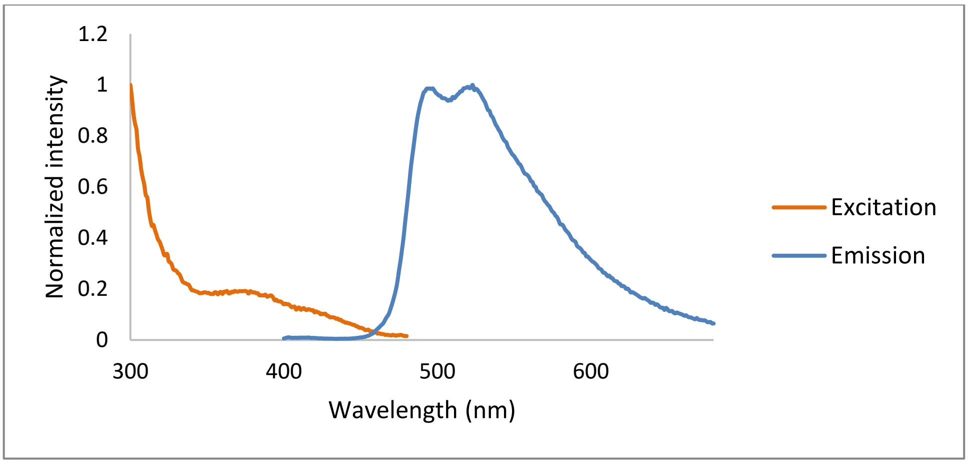

Figure S33. Normalized emission and excitation spectrum for a 1 x 10^{-5} M solution of complex **6** in 2-MeTHF at 298 K.

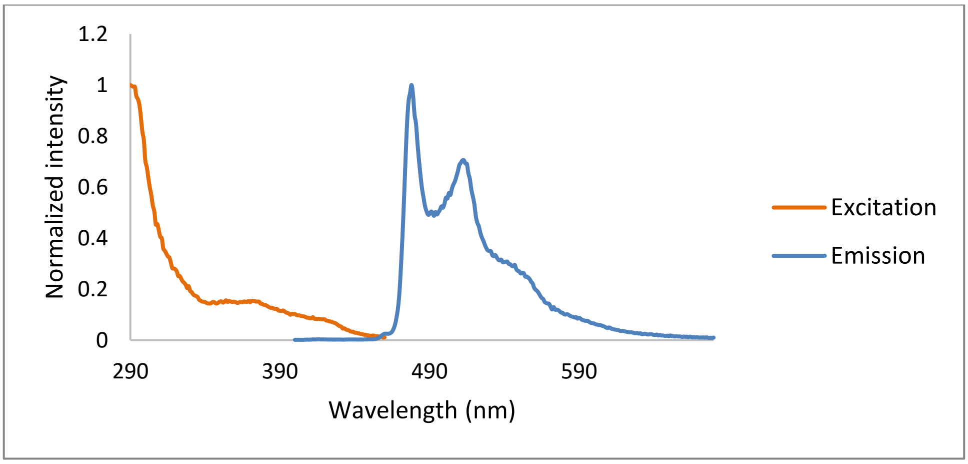

Figure S34. Normalized emission and excitation spectrum for a 1 x 10^{-5} M solution of complex **6** in 2-MeTHF at 77 K.

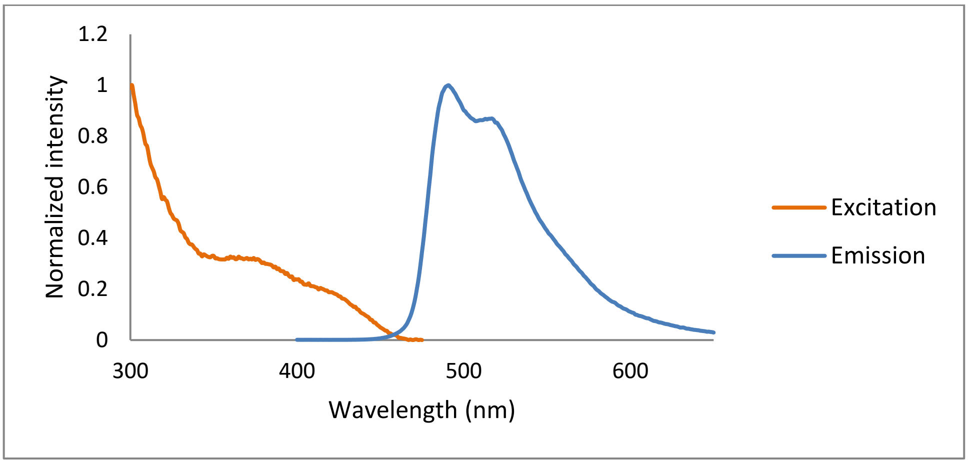

Figure S35. Normalized emission and excitation spectrum of complex **7** in PMMA film (5 wt %) at 298 K.

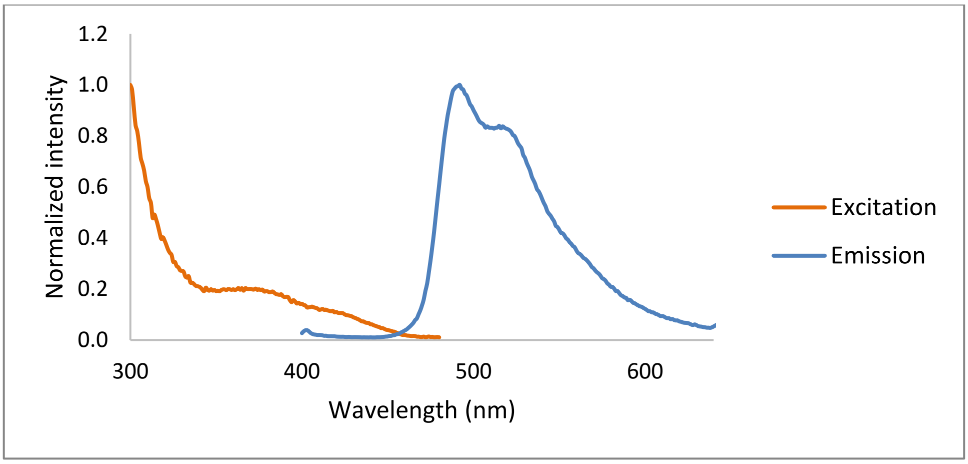

Figure S36. Normalized emission and excitation spectrum for a 1 x 10^{-5} M solution of complex **7** in 2-MeTHF at 298 K.

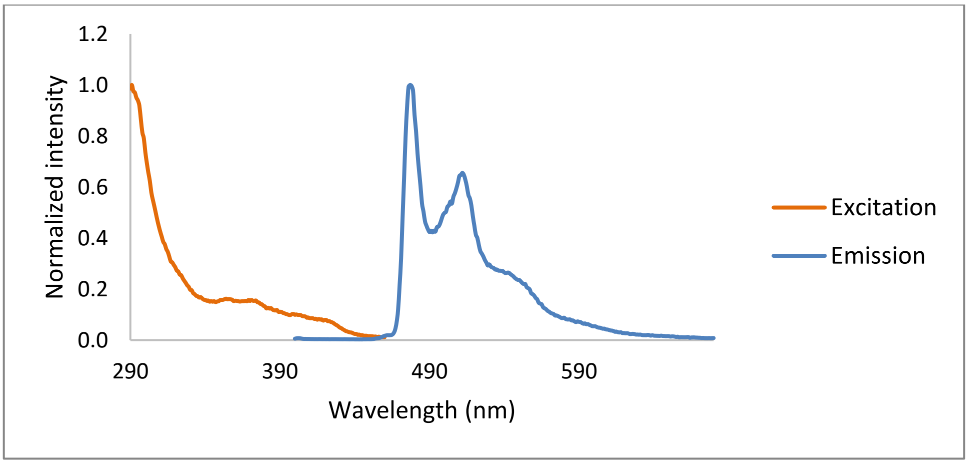

Figure S37. Normalized emission and excitation spectrum for a 1 x 10^{-5} M solution of complex **7** in 2-MeTHF at 77 K.

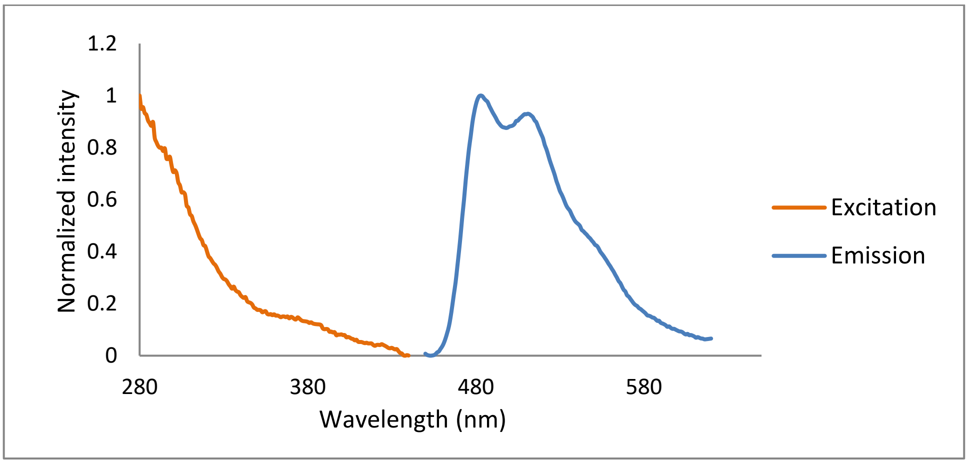

Figure S38. Normalized emission and excitation spectrum of complex **8** in PMMA film (5 wt %) at 298 K.

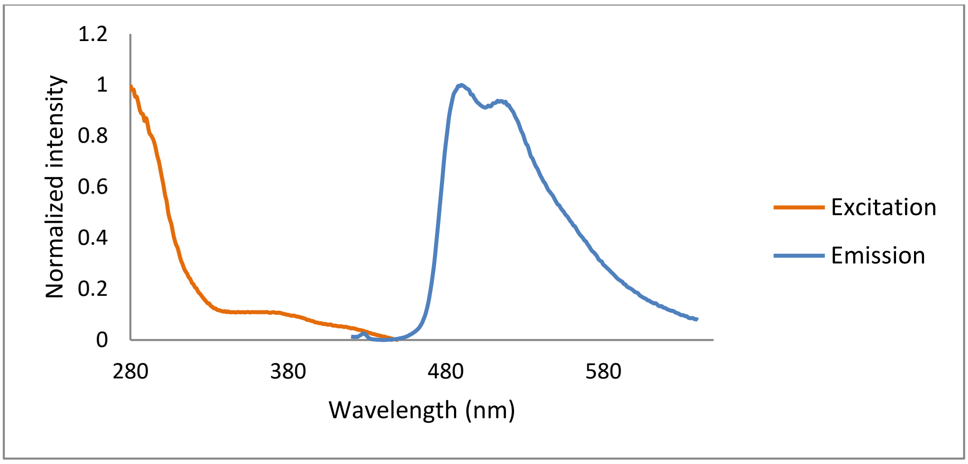

Figure S39. Normalized emission and excitation spectrum for a 1 x 10^{-5} M solution of complex **8** in 2-MeTHF at 298 K.

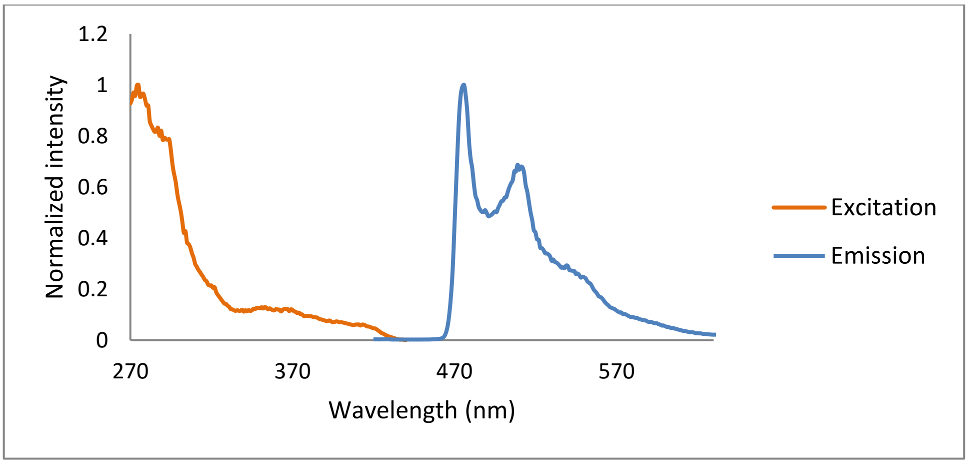

Figure S40. Normalized emission and excitation spectrum for a 1 x 10^{-5} M solution of complex **8** in 2-MeTHF at 77 K.

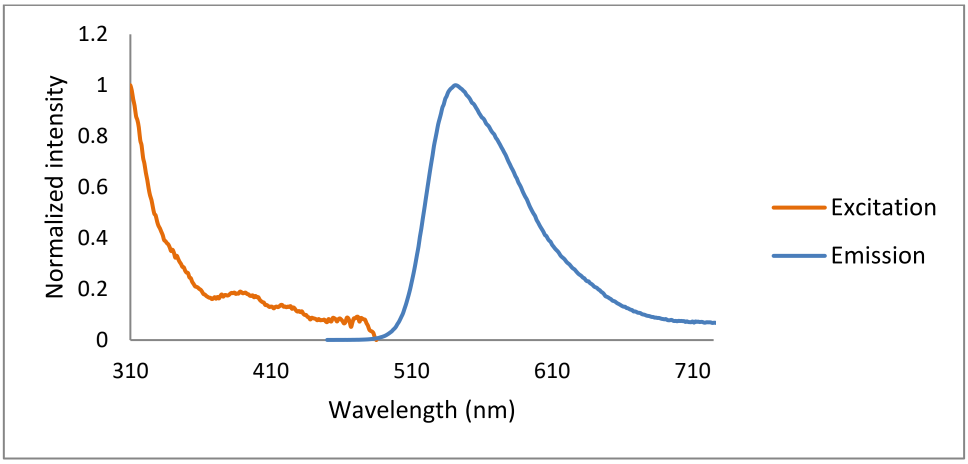

Figure S41. Normalized emission and excitation spectrum of complex **9** in PMMA film (5 wt %) at 298 K.

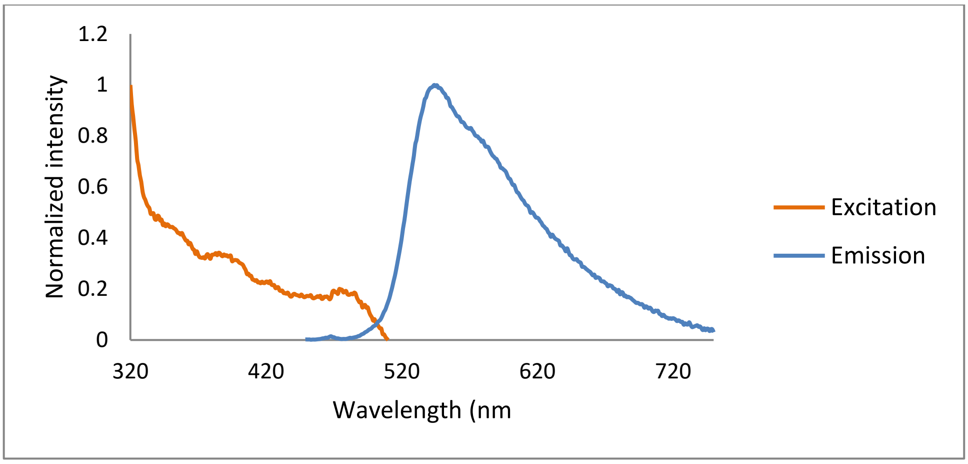

Figure S42. Normalized emission and excitation spectrum for a 1 x 10^{-5} M solution of complex **9** in 2-MeTHF at 298 K.

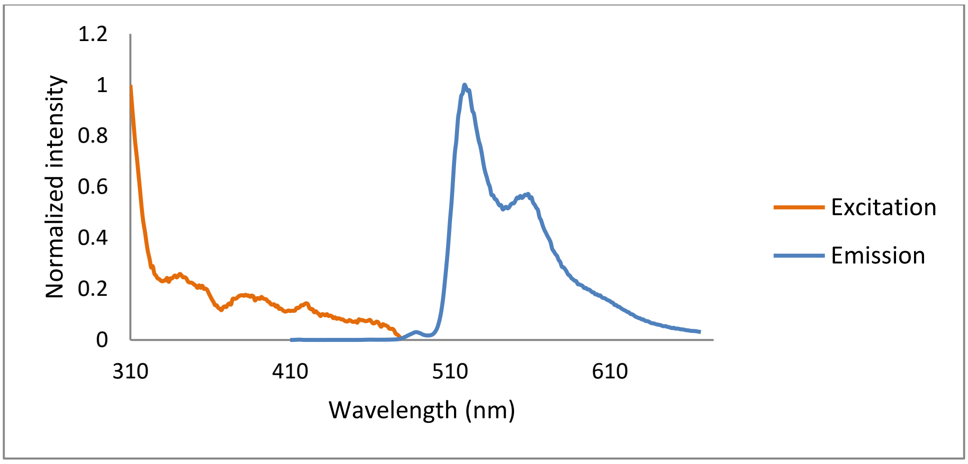

Figure S43. Normalized emission and excitation spectrum for a 1 x 10^{-5} M solution of complex **9** in 2-MeTHF at 77 K.

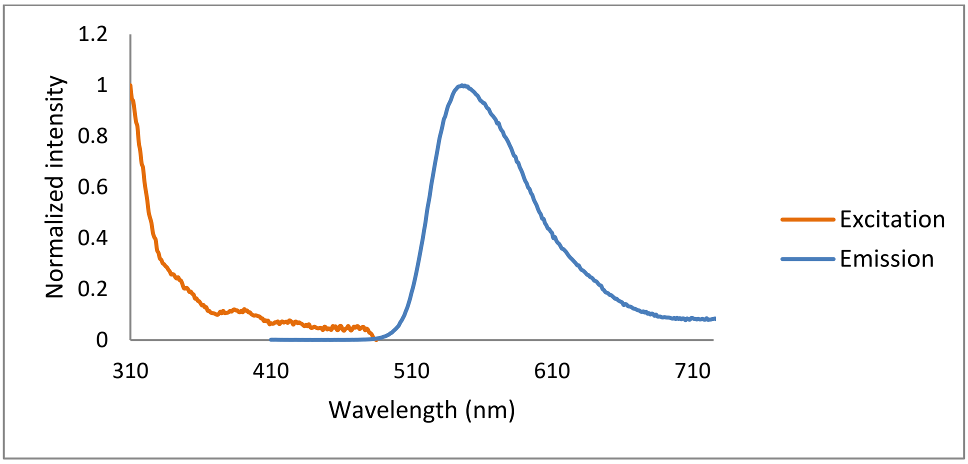

Figure S44. Normalized emission and excitation spectrum of complex **10** in PMMA film (5 wt %) at 298 K.

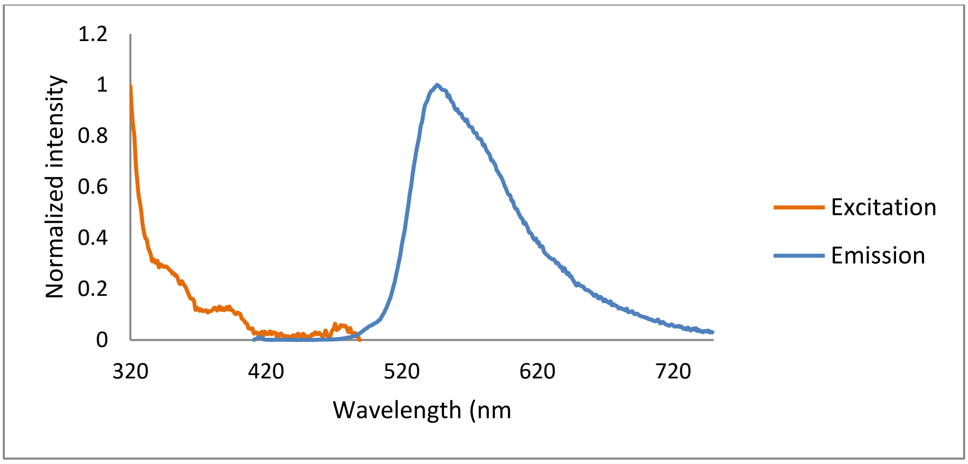

Figure S45. Normalized emission and excitation spectrum for a 1 x 10^{-5} M solution of complex **10** in 2-MeTHF at 298 K.

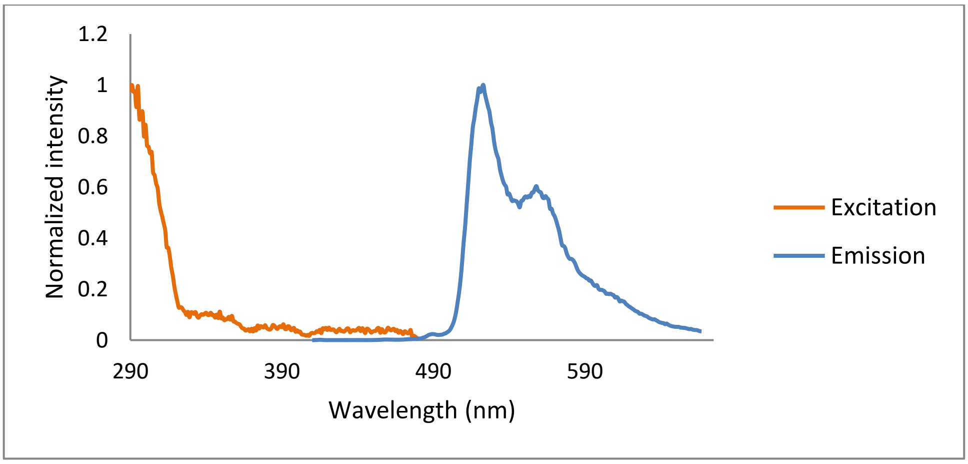

Figure S46. Normalized emission and excitation spectrum for a 1 x 10^{-5} M solution of complex **10** in 2-MeTHF at 77 K.

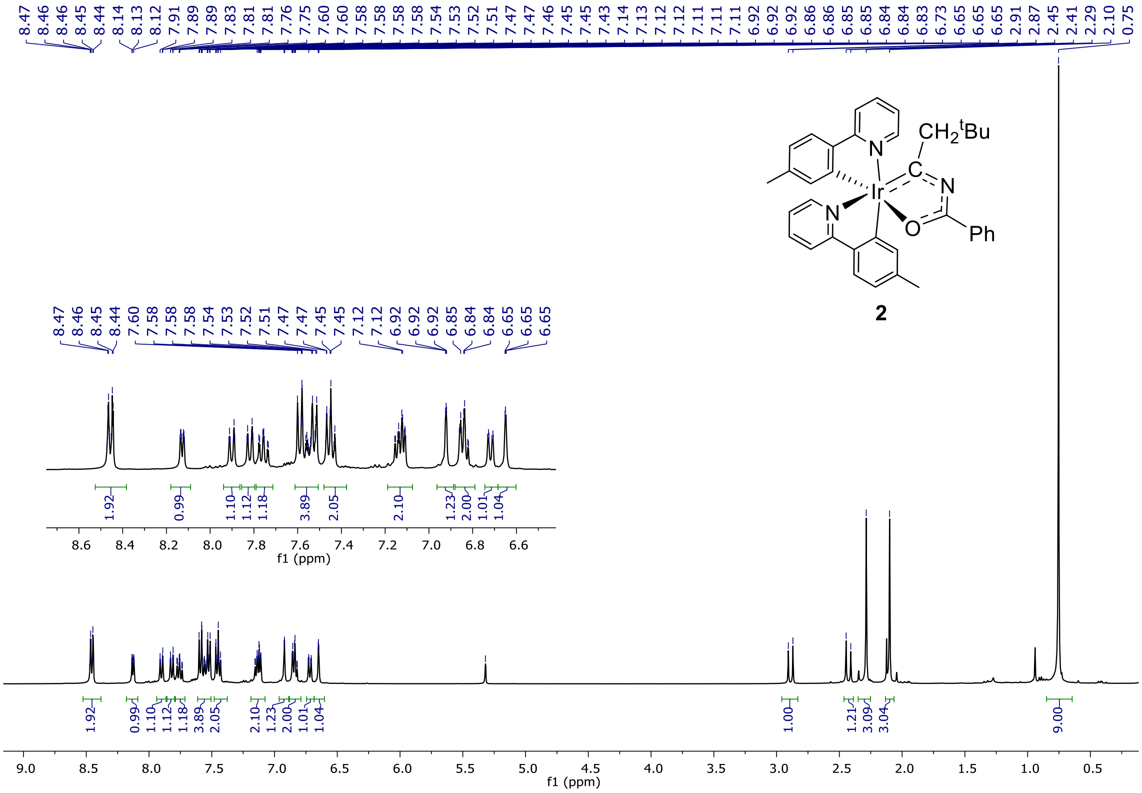

Figure S47. ^{1}H-NMR (400 MHz, CD_2Cl_2, 298 K) of complex **2**.

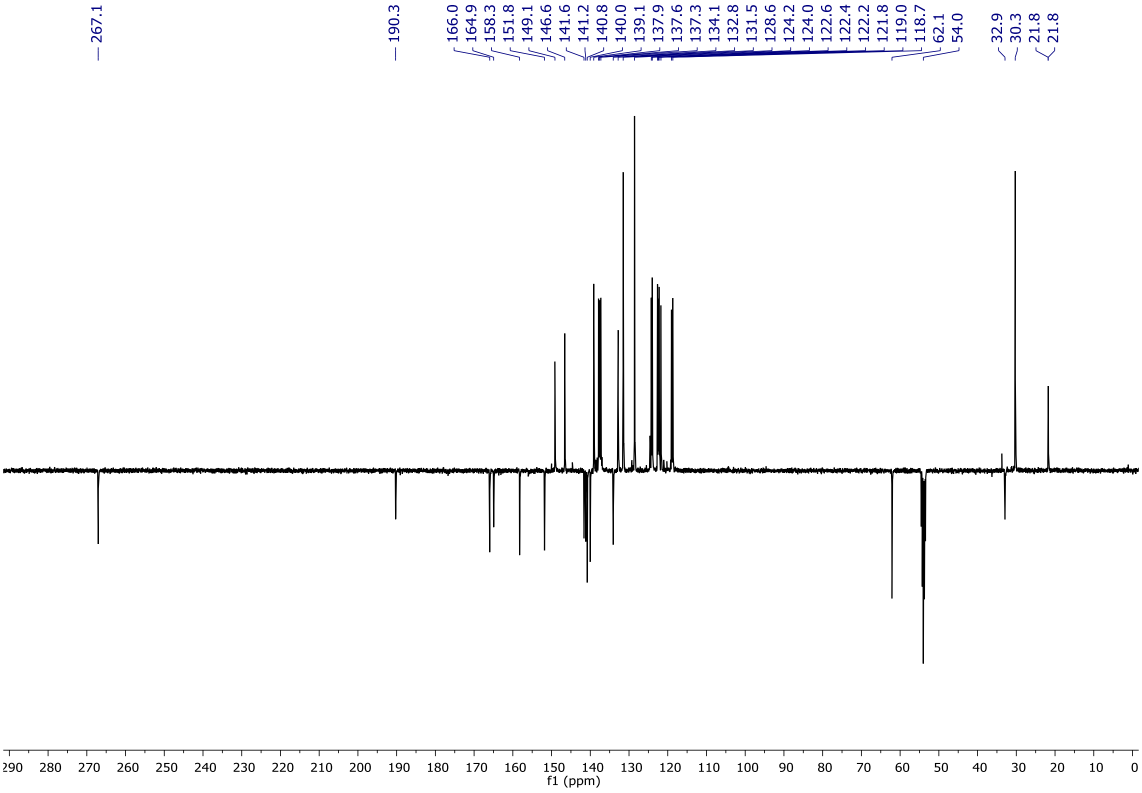

Figure S48. $^{13}C\{^{1}H\}$-APT NMR (101 MHz, CD_2Cl_2, 253 K) of complex **2**.

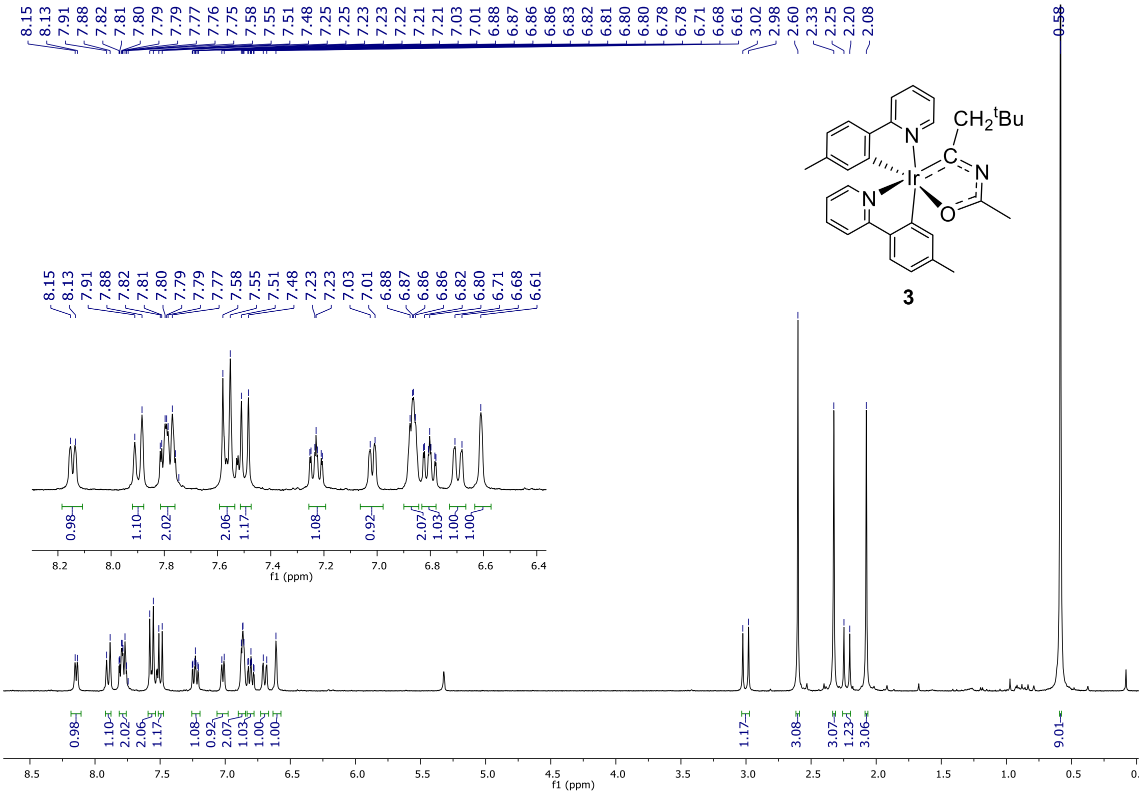

Figure S49. ^{1}H-NMR (400 MHz, CD_2Cl_2, 298 K) of complex **3**.

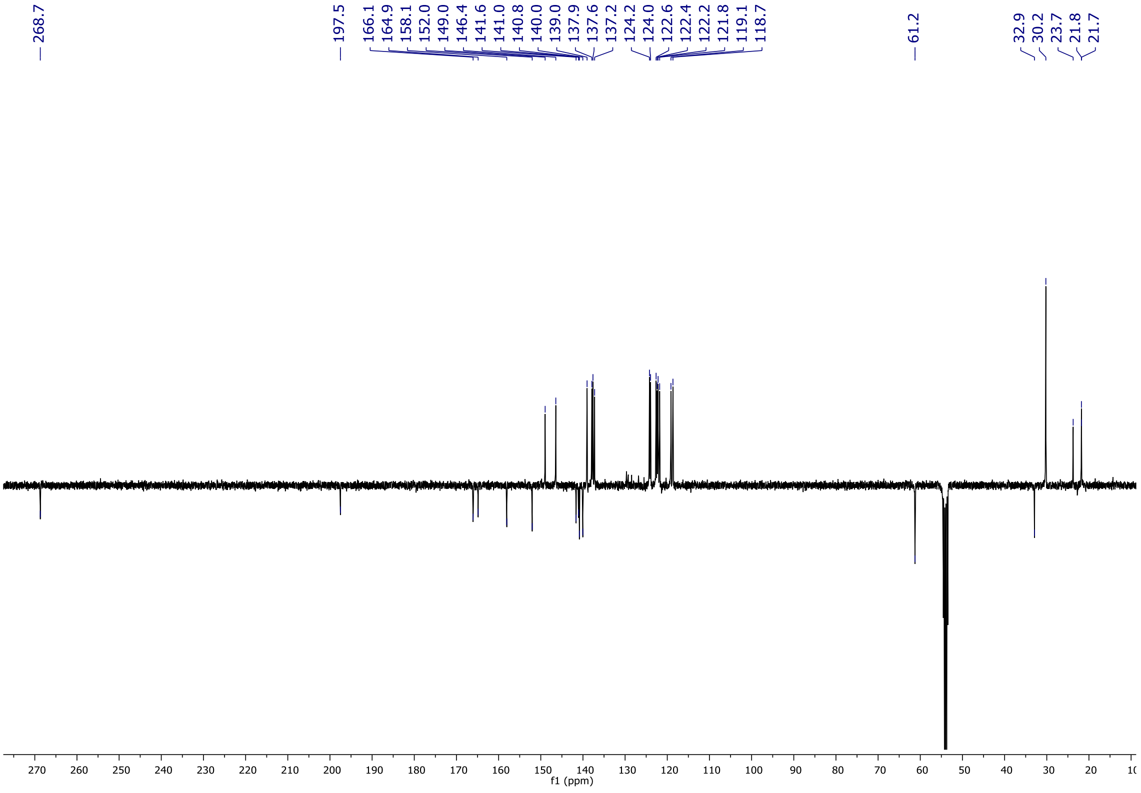

Figure S50. $^{13}C\{^{1}H\}$-APT NMR (101 MHz, CD_2Cl_2, 253 K) of complex **3**.

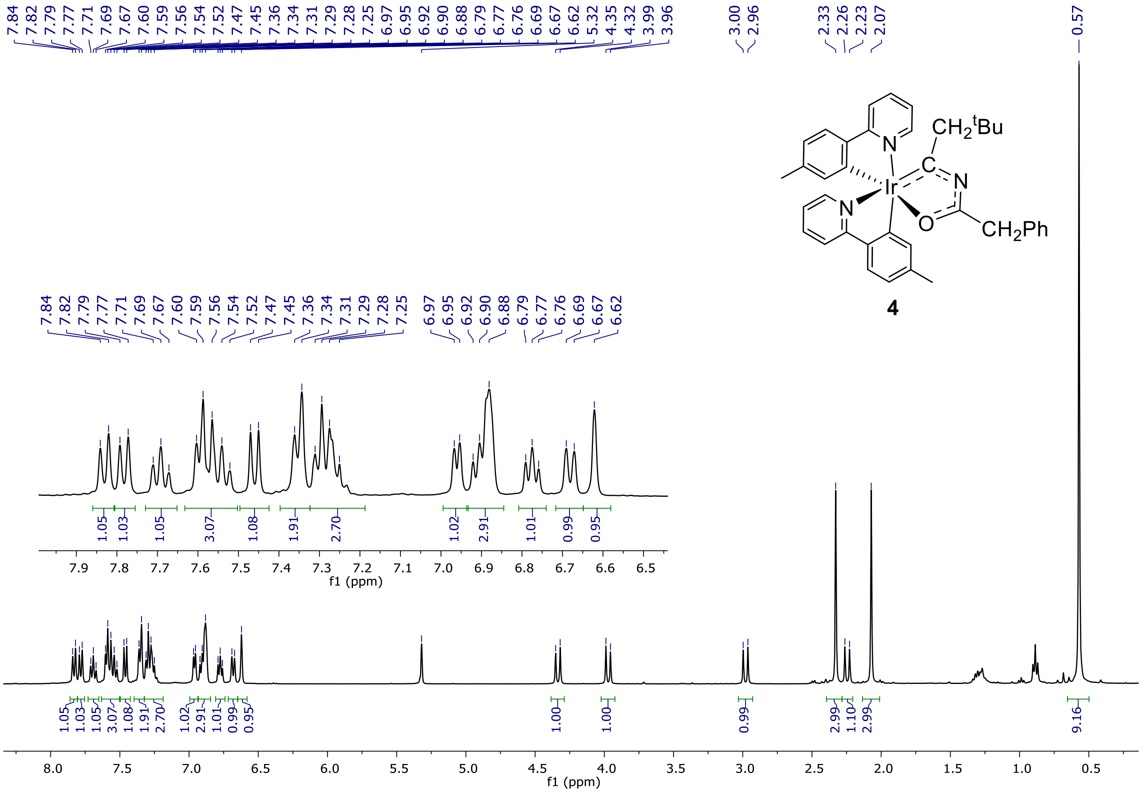

Figure S51. ^{1}H-NMR (400 MHz, CD_2Cl_2, 298 K) of complex **4**.

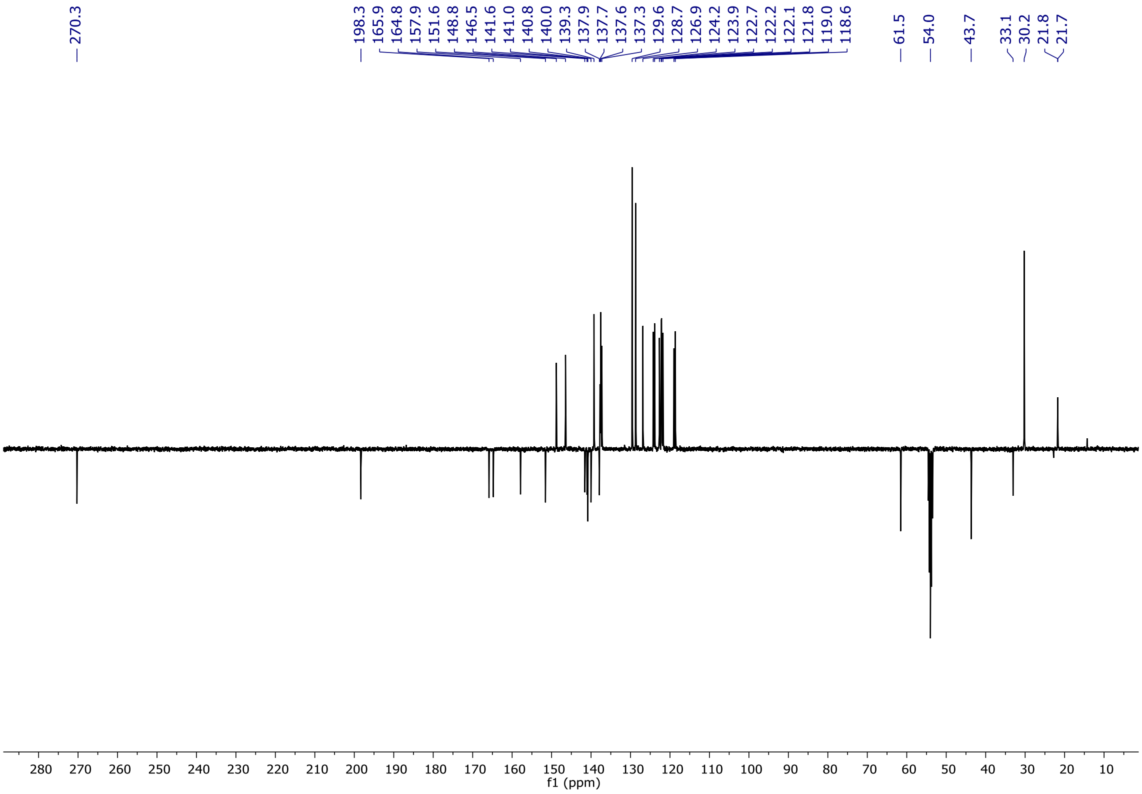

Figure S52. $^{13}C\{^{1}H\}$-APT NMR (101 MHz, CD_2Cl_2, 253 K) of complex **4**.

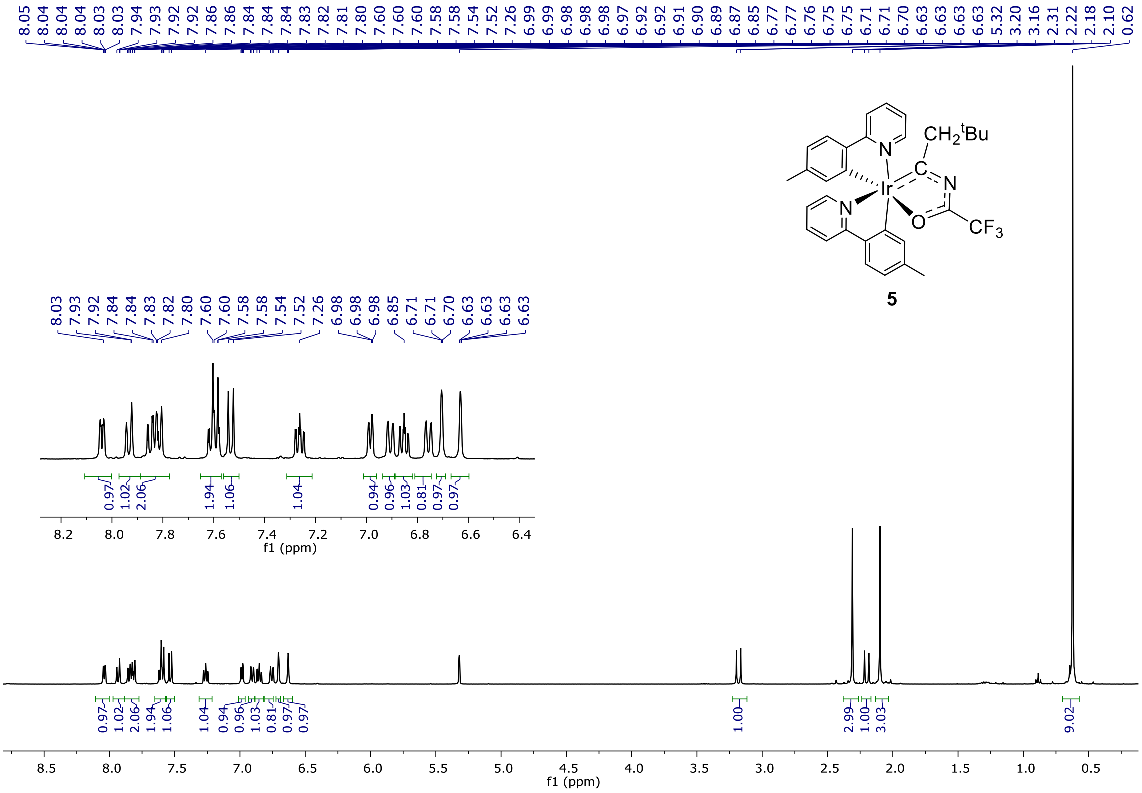

Figure S53. ^{1}H-NMR (400 MHz, CD_2Cl_2, 298 K) of complex **5**.

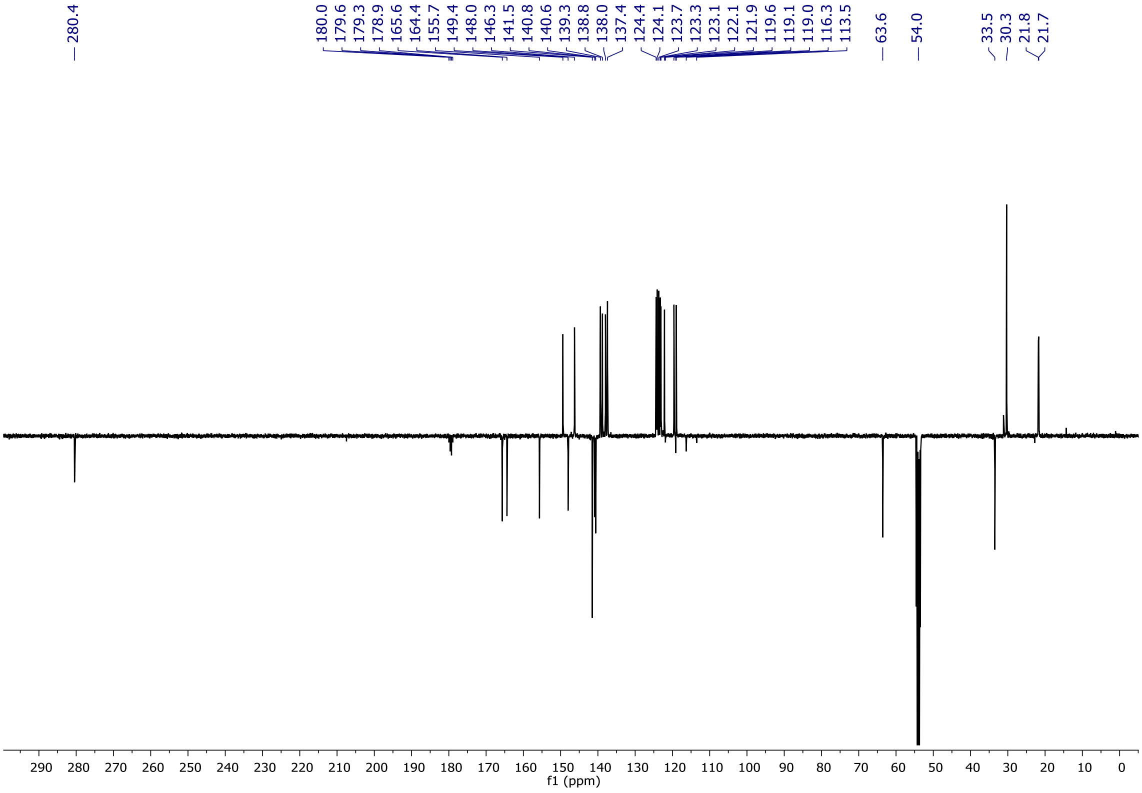

Figure S54. $^{13}C\{^{1}H\}$-APT NMR (101 MHz, CD_2Cl_2, 253 K) of complex **5**.

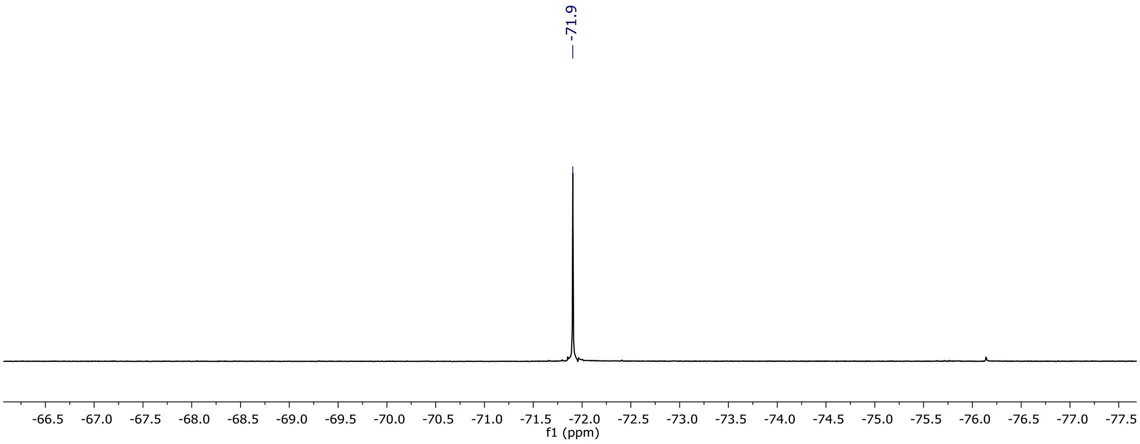

Figure S55. $^{19}F\{^1H\}$ NMR (376.5 MHz, CD_2Cl_2, 298 K) of complex **5**.

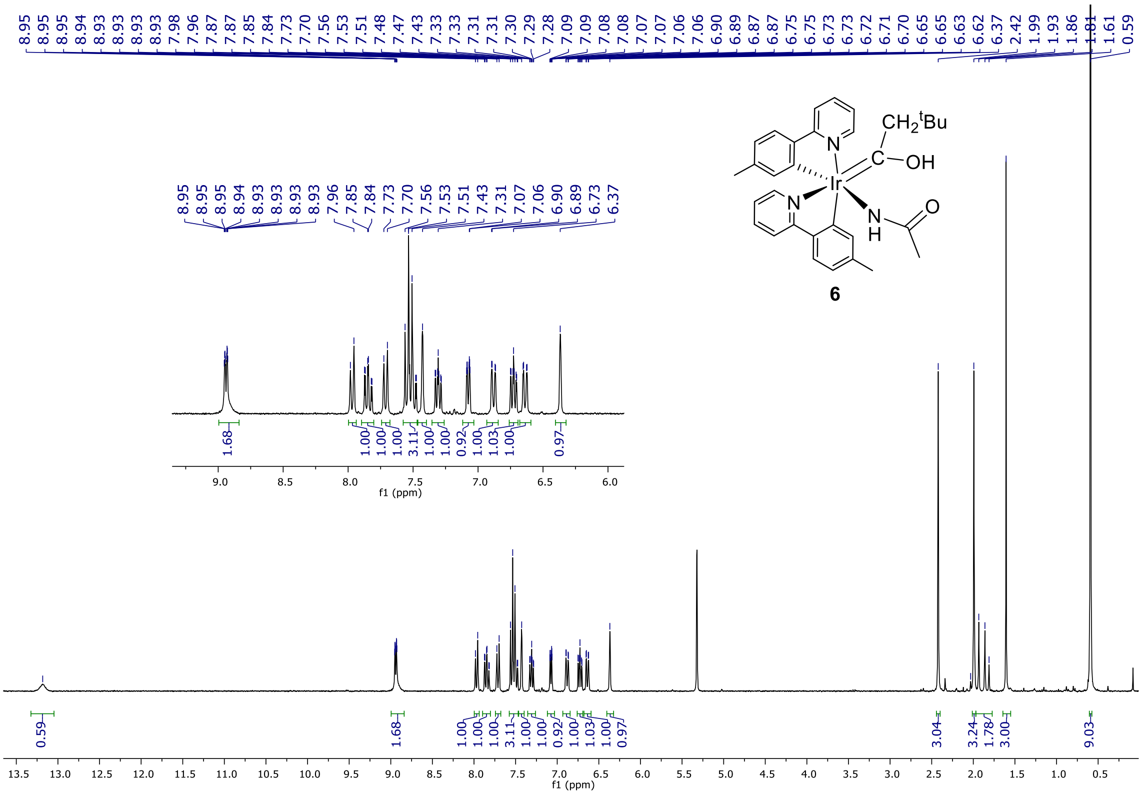

Figure S56. ^{1}H-NMR (300 MHz, CD_2Cl_2, 298 K) of complex **6**.

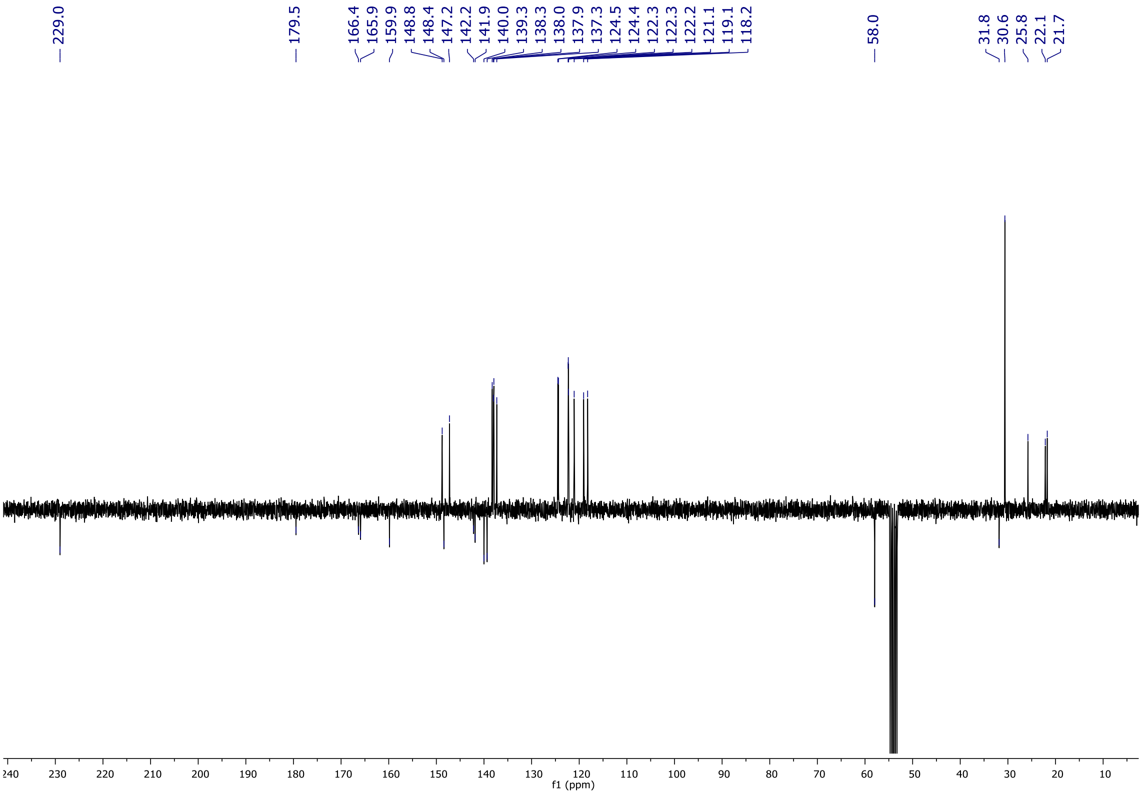

Figure S57. $^{13}C\{^{1}H\}$-APT NMR (75 MHz, CD_2Cl_2, 253 K) of complex **6**.

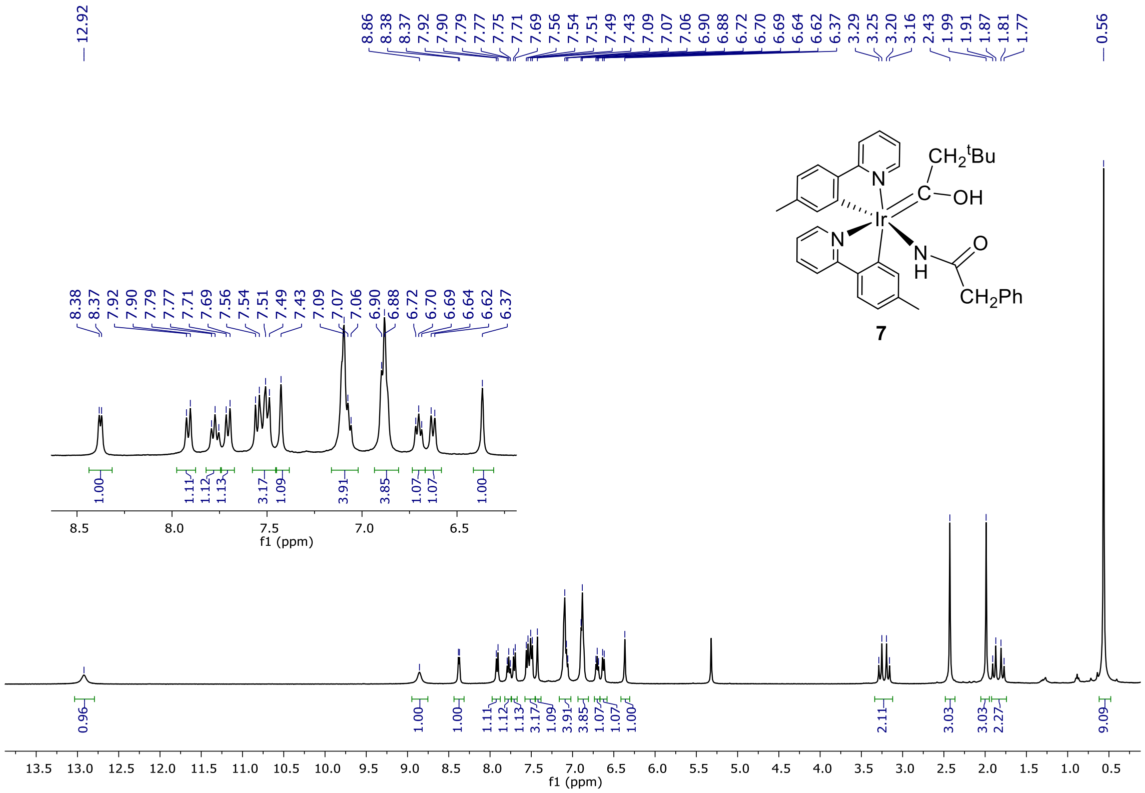

Figure S58. ^{1}H-NMR (300 MHz, CD_2Cl_2, 298 K) of complex **7**.

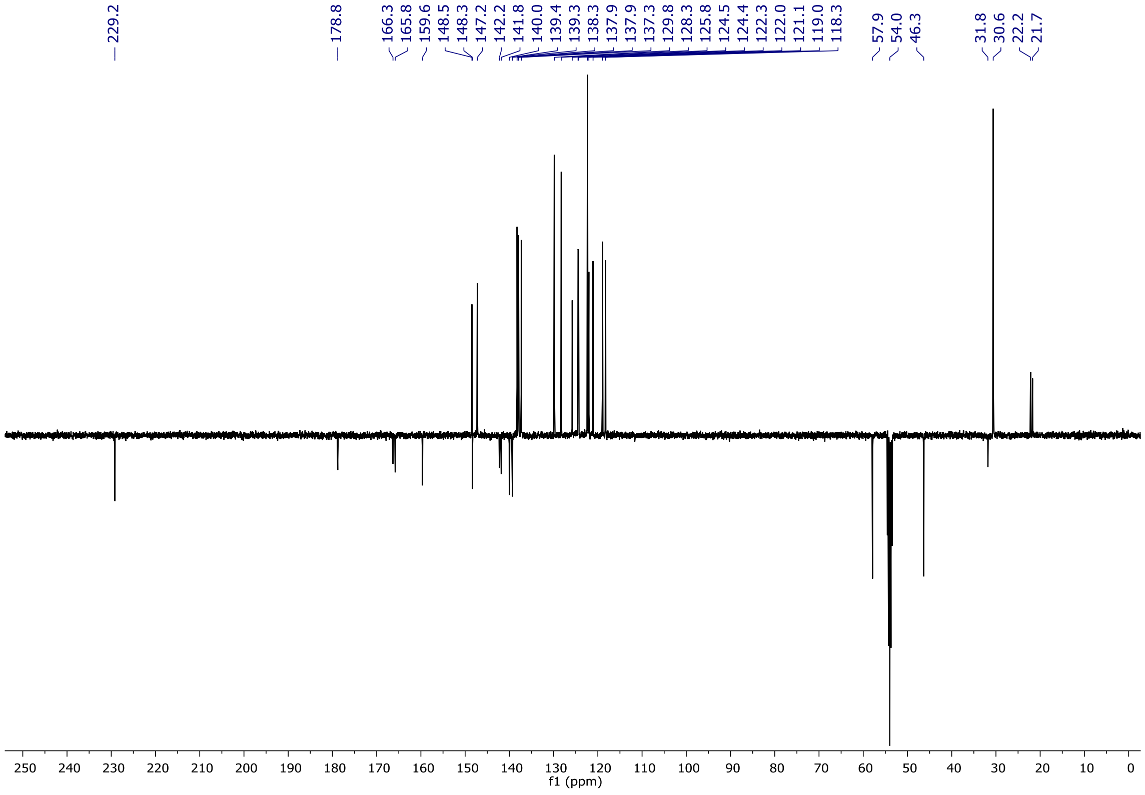

Figure S59. $^{13}C\{^{1}H\}$-APT NMR (75 MHz, CD_2Cl_2, 298 K) of complex **7**.

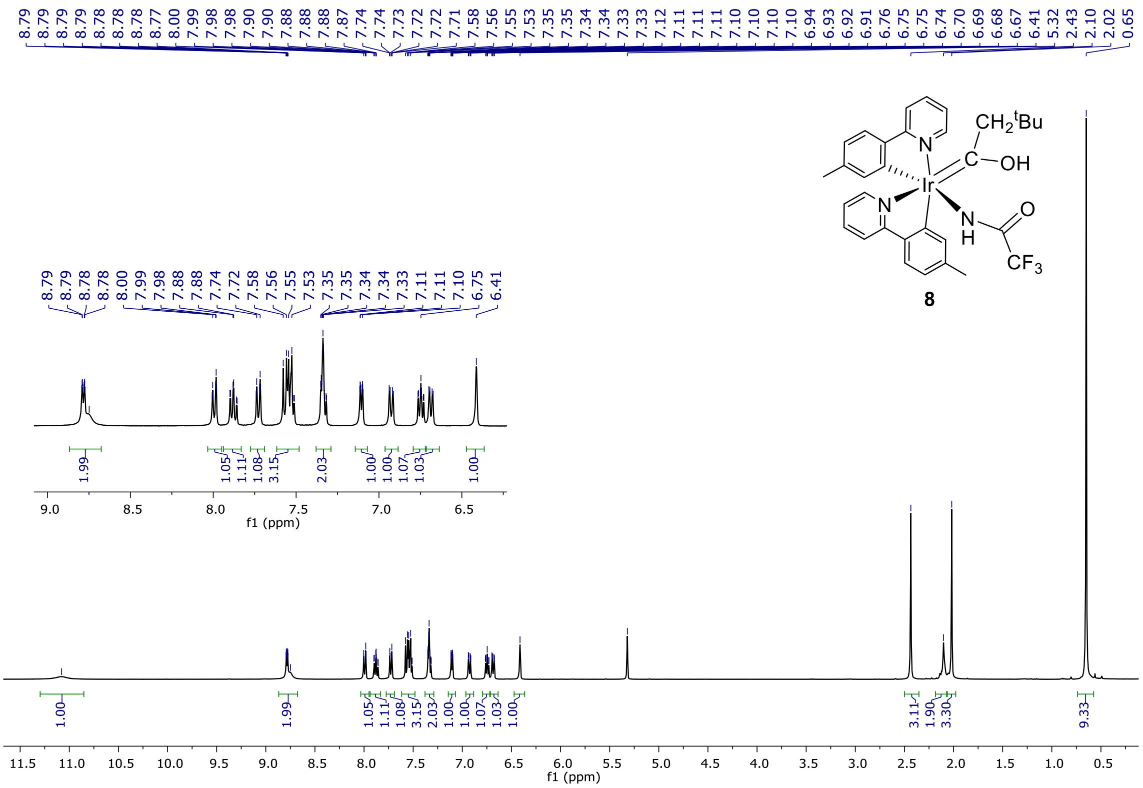

Figure S60. ^{1}H-NMR (300 MHz, CD_2Cl_2, 298 K) of complex **8**.

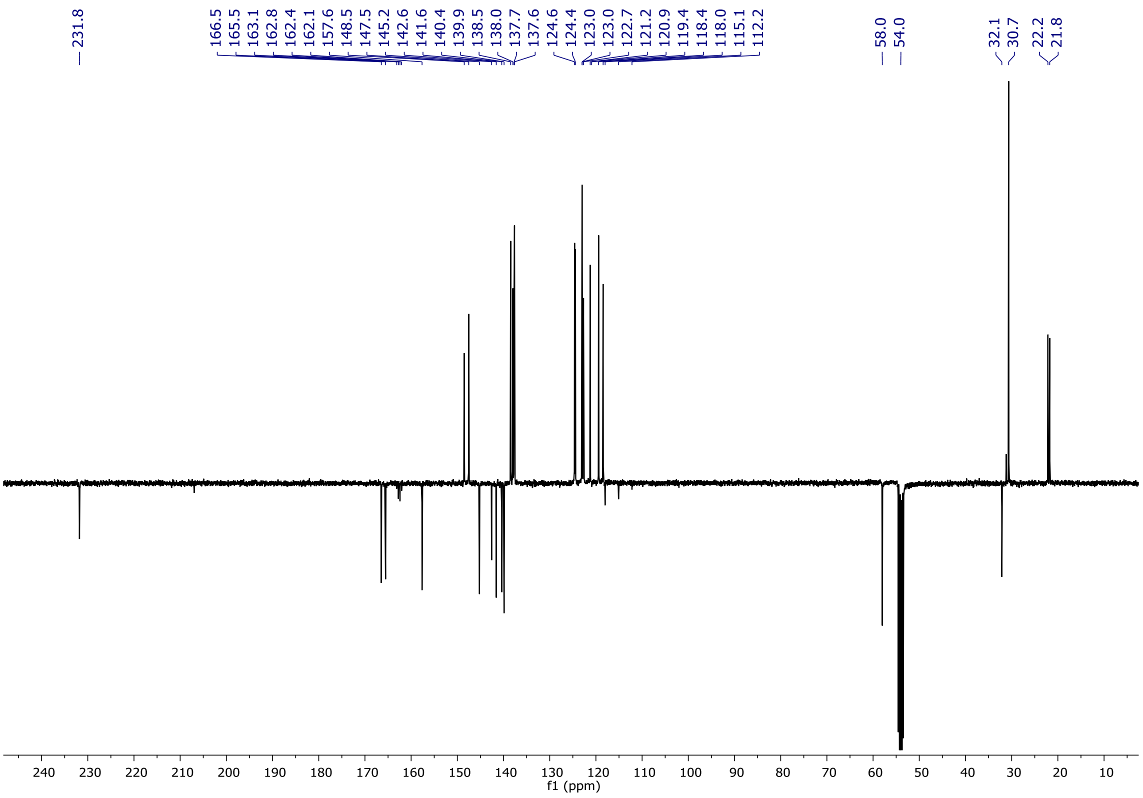

Figure S61. $^{13}C\{^{1}H\}$-APT NMR (101 MHz, CD_2Cl_2, 298 K) of complex **8**.

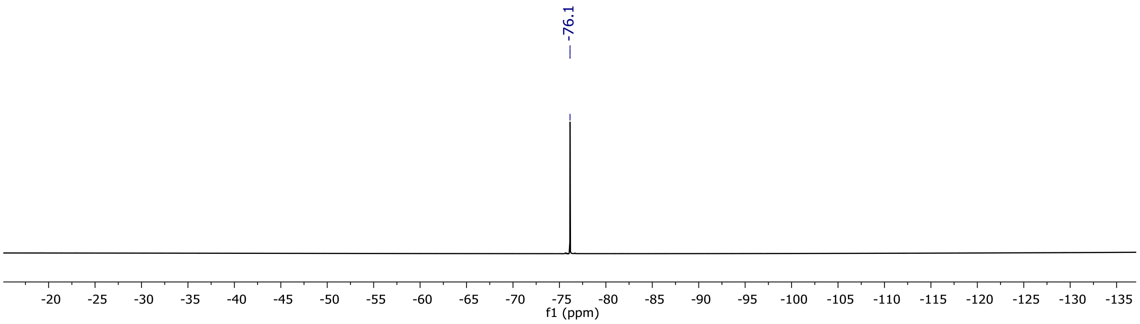

Figure S62. $^{19}F\{^{1}H\}$ NMR (376.5 MHz, CD_2Cl_2, 298 K) of complex **8**.

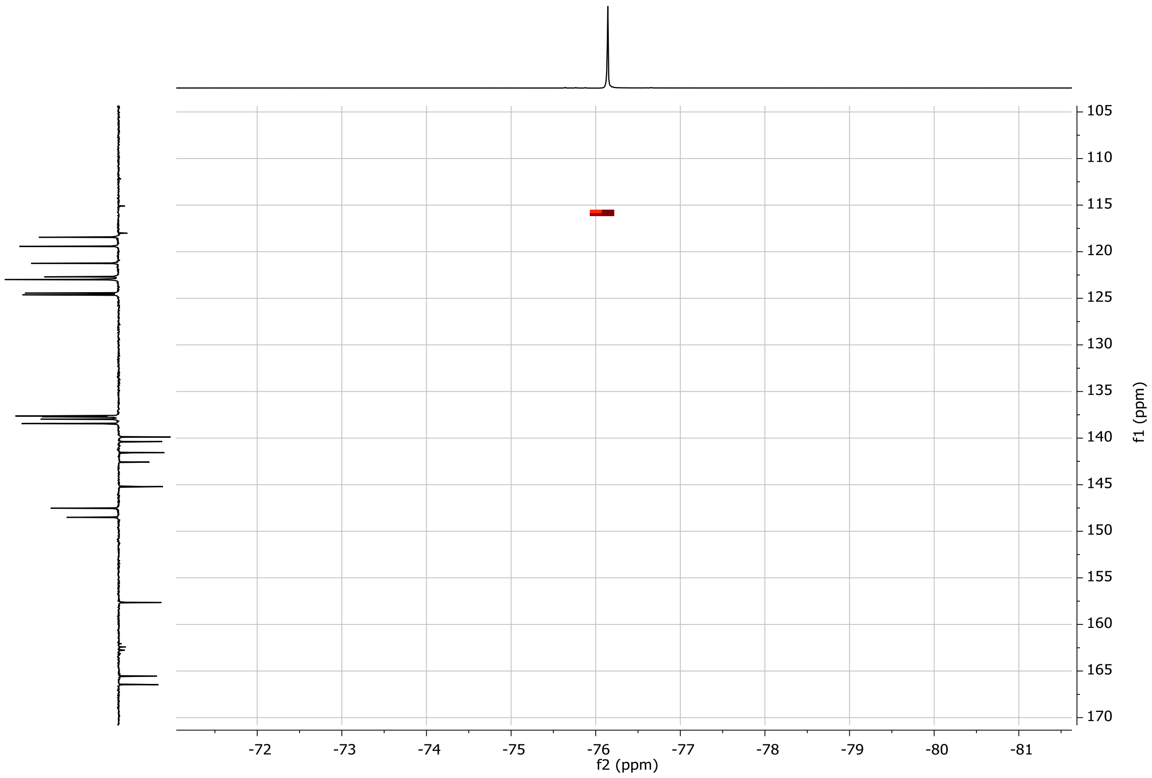

Figure S63. ^{19}F-^{13}C HMQC NMR (400 MHz, CD_2Cl_2, 298 K) of complex **8**.

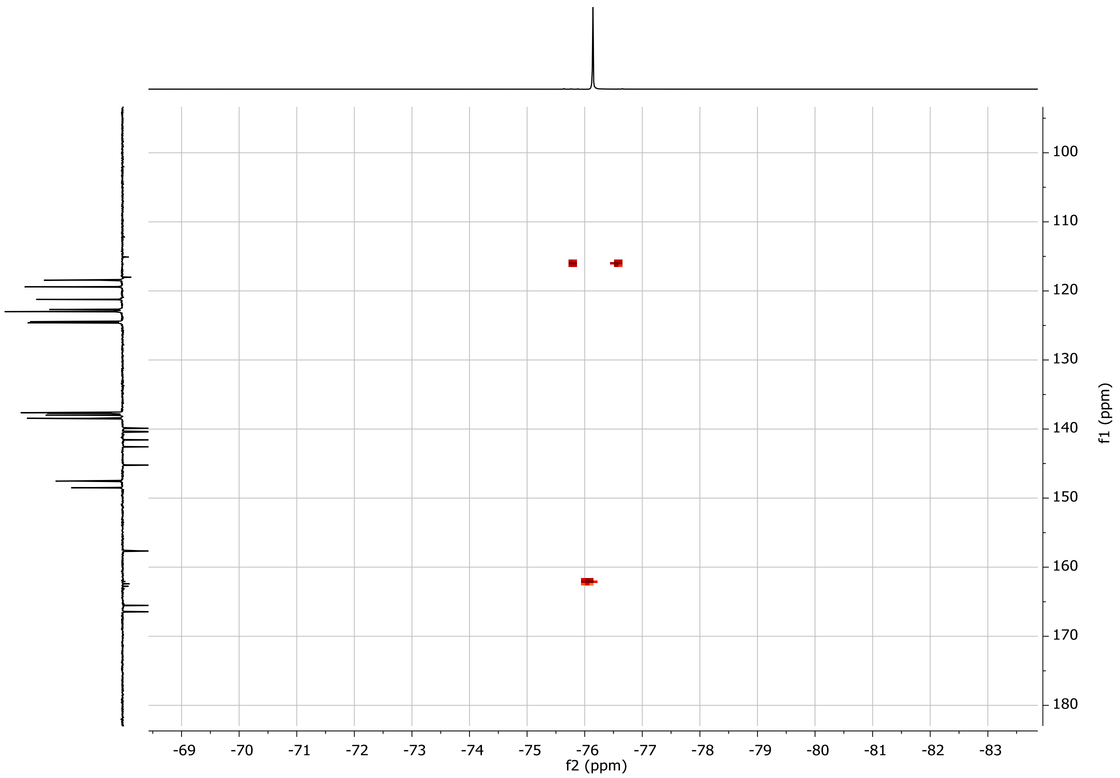

Figure S64. ^{19}F-^{13}C HMBC NMR (376.5 MHz, CD_2Cl_2, 298 K) of complex **8**.

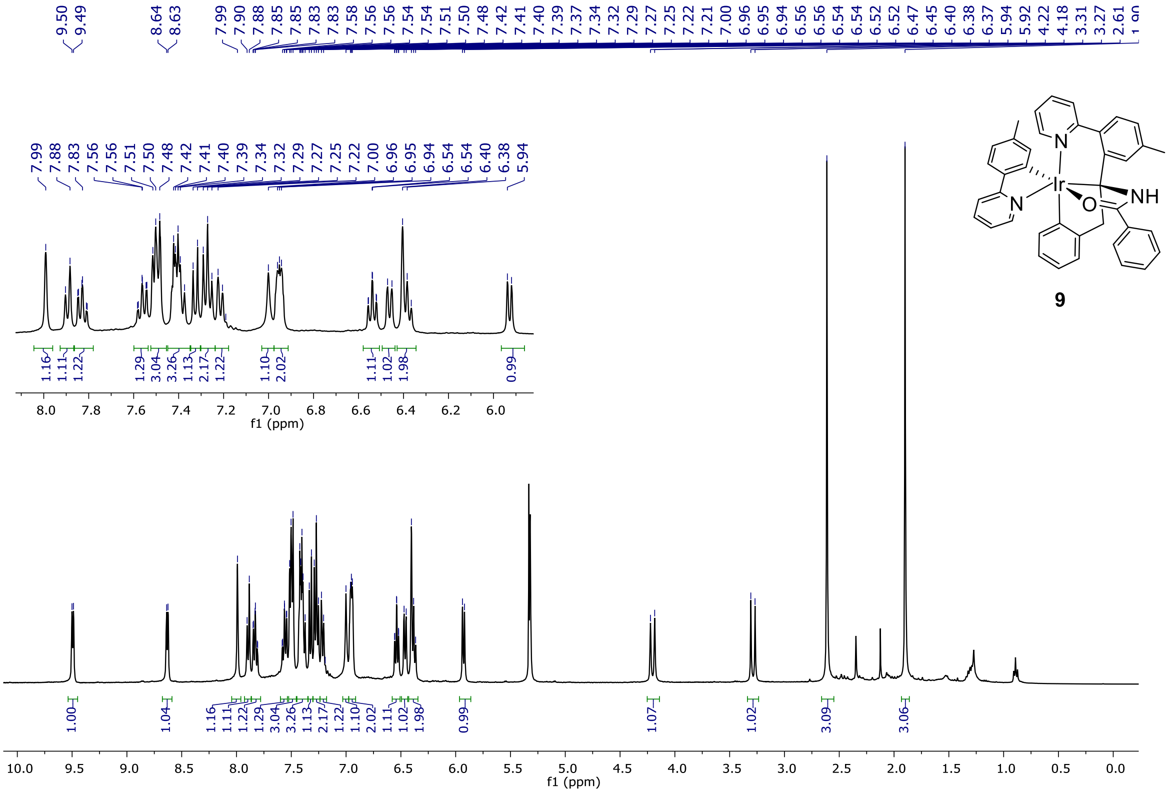

Figure S65. ^{1}H-NMR (400 MHz, CD_2Cl_2, 298 K) of complex **9**.

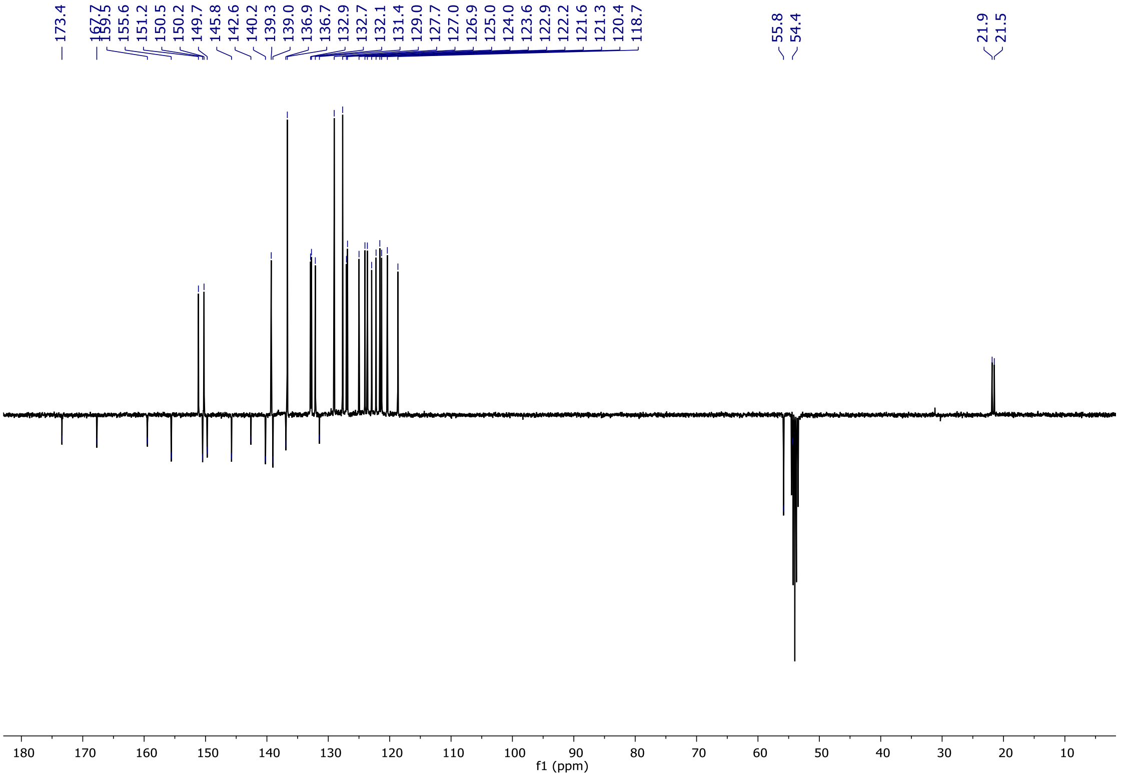

Figure S66.^{13}C{^{1}H}-APT NMR (101 MHz, CD_2Cl_2, 298 K) of complex **9**.

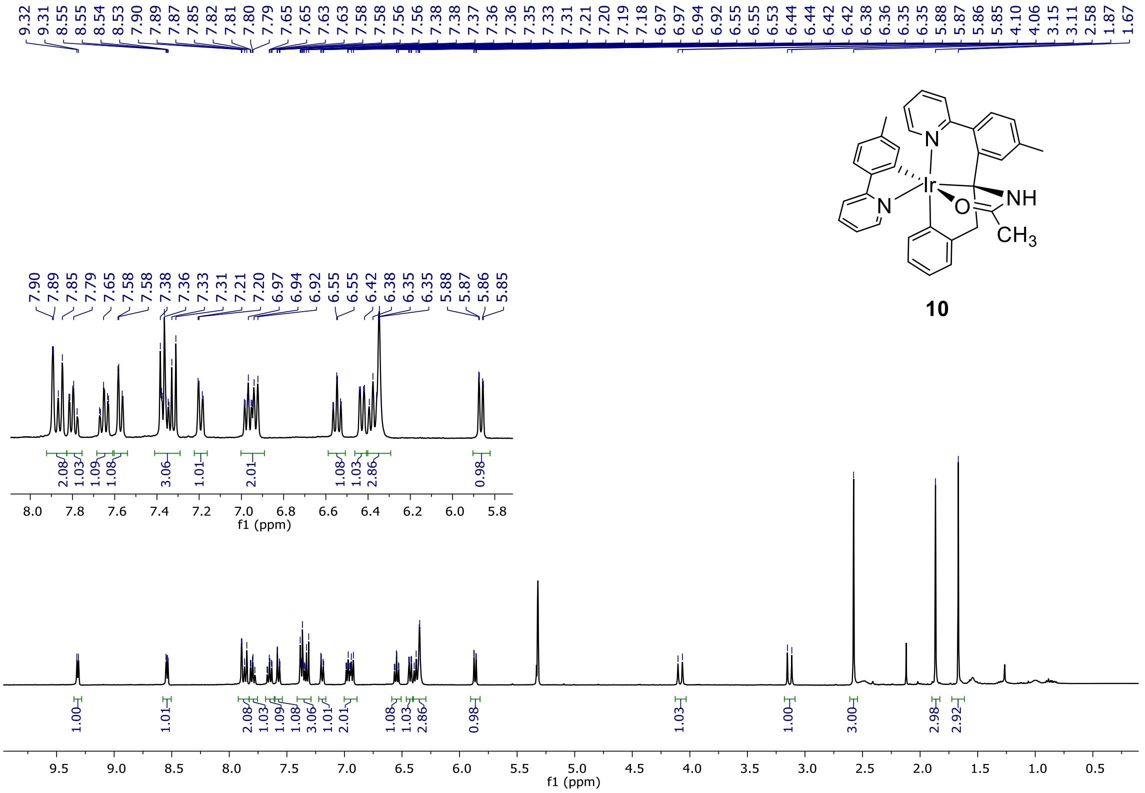

Figure S67. ^{1}H-NMR (400 MHz, CD_2Cl_2, 298 K) of complex **10**.

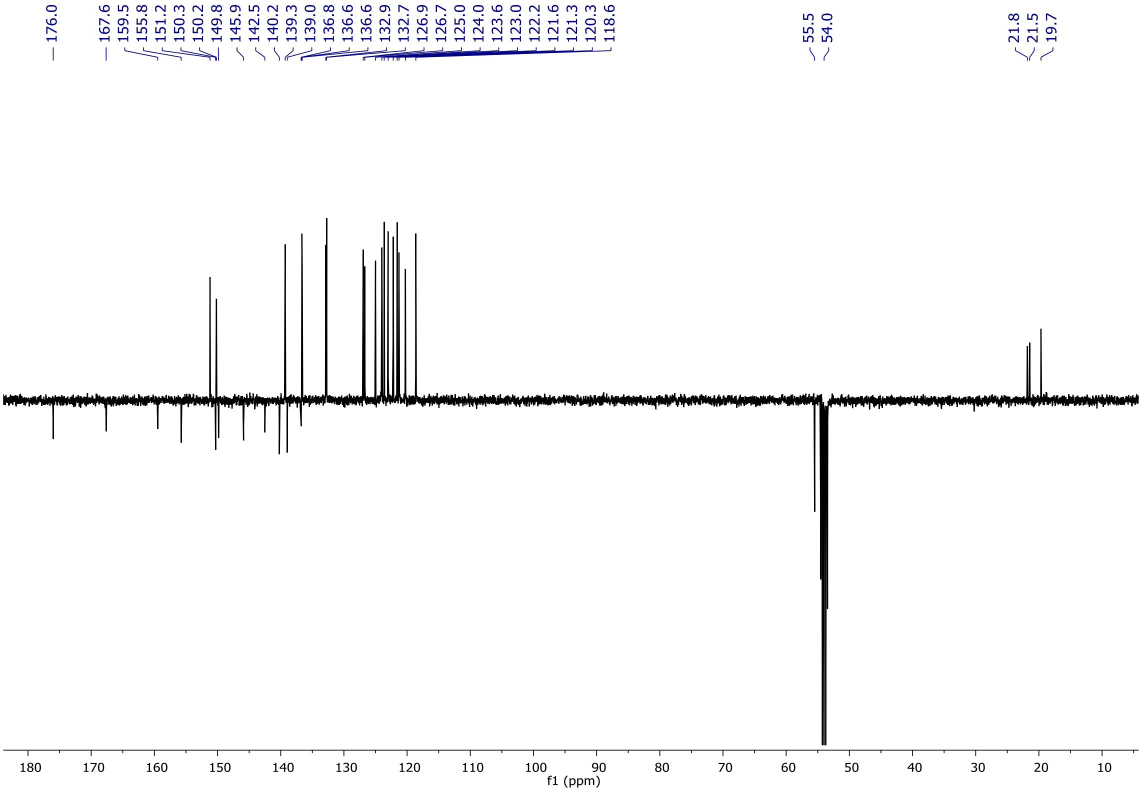

Figure S68. $^{13}C\{^{1}H\}$-APT NMR (101 MHz, CD_2Cl_2, 298 K) of complex **10**.

References

(1) Blessing, R. H. *Acta Crystallogr.* **1995**, *A51*, 33. SADABS: Area-detector absorption correction; Bruker- AXS, Madison, WI, 1996.

(2) SHELXL-2016/6. Sheldrick, G. M. *Acta Cryst.* **2008**, *A64*, 112-122.

(3) (a) Lee, C.; Yang, W.; Parr, R. G. Development of the Colle-Salvetti correlationenergy formula into a functional of the electron density. Phys. Rev. B 1988, 37, 785– 789. (b) Becke, A. D. Density-functional exchange-energy approximation with correct asymptotic behavior. J. Chem. Phys. 1993, 98, 5648–5652. (c) Stephens, P. J.; Devlin, F. J.; Chabalowski, C. F.; Frisch, M. J. Ab Initio Calculation of Vibrational Absorption and Circular Dichroism Spectra Using Density Functional Force Fields. J. Phys. Chem. 1994, 98, 11623–11627.

(4) Grimme, S.; Antony, J.; Ehrlich, S.; Krieg, H. A consistent and accurate ab initio parametrization of density functional dispersion correction (DFT-D) for the 94 elements H-Pu. J. Chem. Phys. 2010, 132, 154104.

(5) Gaussian 09, Revision D.01, Frisch, M. J.; Trucks, G. W.; Schlegel H. B.; Scuseria, G. E.; Robb, M. A.; Cheeseman, J. R.; Scalmani, G.; Barone, V.; Mennucci, B.; Petersson, G. A.; Nakatsuji, H.; Caricato, M.; Li, X.; Hratchian, H. P.; Izmaylov, A. F.; Bloino, J.; Zheng, G.; Sonnenberg, J. L.; Hada, M.; Ehara, M.; Toyota, K.; Fukuda, R.; Hasegawa, J.; Ishida, M.; Nakajima, T.; Honda, Y.; Kitao, O.; Nakai, H.; Vreven, T.; Montgomery, J. A.; Peralta, Jr., J. E.; Ogliaro, F.; Bearpark, M.; Heyd, J. J.; Brothers, E.; Kudin, K. N.; Staroverov, V. N.; Keith, T.; Kobayashi, R.; Normand, J.; Raghavachari, K.; Rendell, A.; Burant, J. C.; Iyengar, S. S.; Tomasi, J.; Cossi, M.;

Rega, N.; S43 Millam, J. M.; Klene, M.; Knox, J. E.; Cross, J. B.; Bakken, V.; Adamo, C.; Jaramillo, J.; Gomperts, R.; Stratmann, R. E.; Yazyev, O.; Austin, A. J.; Cammi, R.; Pomelli, C.; Ochterski, J. W.; Martin, R. L.; Morokuma, K.; Zakrzewski, V. G.; Voth, G. A.; Salvador, P.; Dannenberg, J. J.; Dapprich, S.; Daniels, A. D.; Farkas, O.; Foresman, J. B.; Ortiz, J. V.; Cioslowski, J.; Fox, D. J. Gaussian, Inc., Wallingford CT, 2013.

(6) Andrea, D.; Häußermann, U. M.; Dolg, M.; Stoll, H.; Preuss, H. Energyadjusted ab initio pseudopotentials for the second and third row transition elements. Theor. Chim. Acta 1990, 77, 123–141.

(7) Ehlers, A. W.; Bohme, M.; Dapprich, S.; Gobbi, A.; Hollwarth, A.; Jonas, V.; Kohler, K. F.; Stegmann, R.; Veldkamp, A.; Frenking, G. A set of f-polarization functions for pseudo-potential basis sets of the transition metals SC-Cu, Y-Ag and La-Au. Chem. Phys. Lett. 1993, 208, 111–114.

(8) (a) Hehre, W. J.; Ditchfield, R.; Pople, J. A. Self-Consistent Molecular Orbital Methods. XII. Further Extensions of Gaussian-Type Basis Sets for Use in Molecular Orbital Studies of Organic Molecules. J. Chem. Phys. 1972, 56, 2257–2261. (b) Francl, M. M.; Pietro, W. J.; Hehre, W. J.; Binkley, J. S.; Gordon, M. S.; DeFrees, D. J.; Pople, J. A. Self-consistent molecular orbital methods. XXIII. A polarization-type basis set for second-row elements. J. Chem. Phys. 1982, 77, 3654–3665.

(9) Marenich, A. V.; Cramer, C. J.; Truhlar, D. G. Universal Solvation Model Based on Solute Electron Density and on a Continuum Model of the Solvent Defined by the Bulk Dielectric Constant and Atomic Surface Tensions. J. Phys. Chem. B 2009, 113, 6378–6396.

(10) O'Boyle, N. M.; Tenderholt, A. L.; Langner, K. M. cclib: A Library for Package-Independent Computational Chemistry Algorithms. J. Comput. Chem. 2008, 29, 839−845.

(11) ***NBO 7.0.*** E. D. Glendening, J, K. Badenhoop, A. E. Reed, J. E. Carpenter, J. A. Bohmann, C. M. Morales, P. Karafiloglou, C. R. Landis, and F. Weinhold, Theoretical Chemistry Institute, University of Wisconsin, Madison (2018).
